# Copper-catalyzed transformation of alkyl nitriles to *N*-arylacetamide using diaryliodonium salts†

**DOI:** 10.1039/d1ra02305e

**Published:** 2021-04-28

**Authors:** Romain Sallio, Pierre-Adrien Payard, Paweł Pakulski, Iryna Diachenko, Indira Fabre, Sabine Berteina-Raboin, Cyril Colas, Ilaria Ciofini, Laurence Grimaud, Isabelle Gillaizeau

**Affiliations:** Institute of Organic and Analytical Chemistry, ICOA UMR 7311 CNRS, Université d'Orléans rue de Chartres 45100 Orléans France Isabelle.gillaizeau@univ-orleans.fr; Laboratoire des Biomolécules, LBM, Département de chimie, École normale supérieure, PSL University, Sorbonne Université, CNRS 75005 Paris France laurence.grimaud@ens.psl.eu; Institute of Chemistry for Health and Life Sciences, I-CLeHS, Chimie ParisTech, PSL University, CNRS 75005 Paris France

## Abstract

This work reports a simple and efficient method for the copper-catalyzed redox-neutral transformation of alkyl nitriles using eco-friendly diaryliodonium salts and leading to *N*-arylacetamides. The method features high efficiency, broad substrate scope and good functional group tolerance.

## Abstract 2

In this study, a simple and efficient method for the copper-catalyzed redox-neutral transformation of alkyl nitriles using eco-friendly diaryliodonium salts and leading to *N*-arylacetamides was investigated.

## Graphical abstract

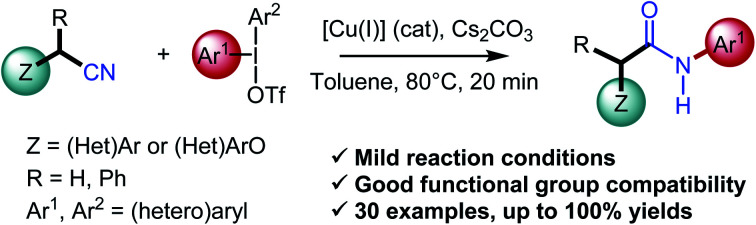

## Introduction

*N*-Arylacetamides constitute the core of many structural motifs found in biologically active compounds, drugs (*e.g.*, lidocaine, atorvastatin), and agrochemicals (*e.g.*, boscalid).^1^ Amide bond formation is a key step that has proved to be one of the most important processes in organic and bioorganic chemistry. For illustration, up to 25% of all current pharmaceuticals contain amide bonds, and polyamides are one of the most widely represented categories of synthetic polymers.^2^ However, in spite of the considerable efforts that have been made for their preparation, and taking into account their synthetic significance, the implementation of atom economical and environmentally-friendly processes is still highly desirable.^3^ Common approaches for the synthesis of *N*-arylacetamides involve the aminolysis of activated carboxylic acid derivatives, such as halides, anhydrides, azides, or activated esters, which are mostly generated in an extra step using hazardous, expensive or waste-intensive reagents.^4^ Alternatively, it may involve peptide coupling reagents, such as carbodiimides or phosphonium salts. However, such processes usually demonstrate a poor atom economy and generate toxic by-products. As a result, catalytic methods have recently been developed to access *N*-arylacetamides.^4*b*,5^ Pioneering work in this field was reported by Goldberg^6^ in the copper catalyzed synthesis of *N*-arylamide from amide through a C–N coupling process, albeit under harsh conditions limiting its broad application in organic synthesis. Buchwald^7^ described an enhanced version by using chelating nitrogen ligands and only 1 mol% of air-stable Cu^I^. More recently, Xiang and Wang studied a copper-catalyzed amination of aryl halides with nitriles, which are easily available, in presence of *N*,*N*′-dimethyl-1,2-ethanediamine as the ligand.^8*a*^ Arylboronic acids have also been investigated as coupling partner.^8*b*^ Major breakthroughs by Wang^9^ or Cui^10^ demonstrated an elegant *N*-arylation approach to respectively (aryl)methylamines or *N*-aryl-cyanamide using diaryliodonium salts. The arylation of secondary acyclic amides has also been achieved by Olofsson with diaryliodonium salts under mild and metal-free conditions.^11^ Chen^12^ has also described the copper-catalyzed selective arylation of arylnitriles, however restricted to the use of six-membered cyclic diaryl iodonium salts only.

**Scheme 1:**
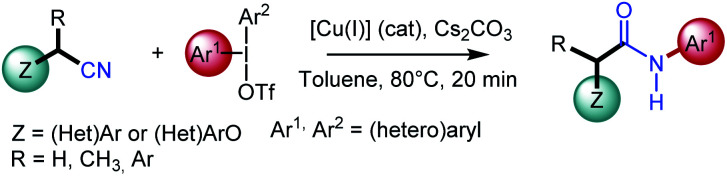
Present work.

Nonetheless, the scope of the aforementioned transformations is mainly limited to (hetero)aryl nitriles and the development of effective general protocols for the arylation of alkyl nitriles with diaryliodonium salts is still highly desirable; only the singular case of acetonitrile having being studied by several groups. In this context and relying on our earlier work in copper-catalyzed reactions,^13^ we envisioned performing *N*-arylamide synthesis by the copper-catalyzed oxidative transformation of readily available alkyl nitriles in presence of diaryliodonium salts. The later has recently aroused considerable interest as they advantageously replace iodoarenes due to their excellent reactivity and environmentally friendly nature.^14^ Herein, we report the first catalytic, single-step, and redox-neutral transformation of alkyl nitriles acting as amine surrogate into *N*-arylacetamides (Scheme 1). This method also provides a singular way of synthesizing these compounds directly from alkyl nitriles, compared to other well-known methods using the carbon of nitriles as a carbonyl source as in the Ritter reaction^15^ or in the hydration of nitriles.^16^

Our initial investigation started with the study of the copper-mediated arylation of the α-methylphenylacetonitrile 1a, with diphenyliodonium triflate 2a, chosen as model substrates, to identify the optimal reaction conditions (Table 1). At the outset, the reaction was carried out using 2.0 equiv. of Cu(OTf)_2_ in presence of Cs_2_CO_3_ (2 equiv.) as a base (entry 1). The desired target, *N*-arylacetamide 3aa, was formed in a yield of 85% within 2 h in CH_2_Cl_2_ at 80 °C (TLC monitoring). Encouraged by these results, a series of copper salts (*e.g.* Cu(OAc)_2_, Cu_2_O, Cu(CH_3_CN)_4_·PF_6_, CuI) were screened under similar conditions (entries 2–5). Thus, it was found that Cu(OTf)_2_ presented the highest activity and efficiency, and that the reaction could be mediated by a copper(i) salt, albeit with a lower yield (entry 6).^17*a*^ The effect of the solvents was investigated and toluene was found to be the most suitable solvent (entries 7–8). It is worth mentioning that a satisfying yield of 78% was obtained by using environmentally-benign dimethylcarbonate (DMC) (entry 8). Lowering the temperature of the reaction was unsatisfactory (entry 9). Finally, the best conditions were found using 0.3 equiv. of the (CuOTf)_2_.toluene complex leading to the quantitative formation of the *N*-arylacetamide 3aa within only 20 min (entries 10–11).^17*b*^ Control experiments showed no reactivity in the absence of catalyst or base. Moreover, in comparison with other inorganic bases (*i.e.* K_2_CO_3_, NaOH…), Cs_2_CO_3_ was the best base for the present transformation. Altering the iodonium salt counterion demonstrated that weakly coordinating anions BF_4_^−^, PF_6_^−^ gave poor results.^17*c*^ To prove the scalability of this transformation, the reaction was performed using 14 mmol of 1a (scaling factor: 28) yielding 3aa in good yield (95%) (entry 11). Furthermore, by applying Chen's conditions,^12^ the reaction of benzyl cyanide 1a and diphenyliodonium triflate 2a failed (entry 12).

**Table 1:** Optimization studies.^a^^,^^e^

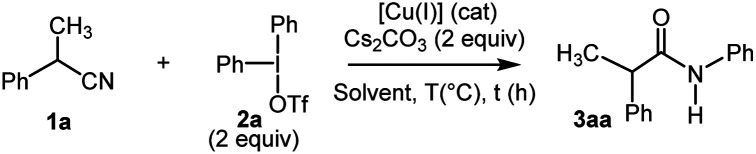					
Entry	Catalyst (equiv.)	Solvent	*T* °C	Time	Yield (%)
1	Cu(OTf)_2_ (2)	CH_2_Cl_2_	80	2 h	85
2	Cu(OAc)_2_ (2)	CH_2_Cl_2_	80	2 h	0
3	Cu_2_O (2)	CH_2_Cl_2_	80	2 h	0
4	Cu(CH_3_CN)_4_·PF_6_ (2)	CH_2_Cl_2_	80	2 h	9
5	CuI (2)	CH_2_Cl_2_	60	2 h	30
6	Cu(OTf)_2_ (2)	Toluene	80	2 h	99
7	Cu(OTf)_2_ (2)	DCE	80	2 h	61
8	Cu(OTf)_2_ (2)	DMC	80	2 h	78
9	Cu(OTf)_2_ (2)	CH_2_Cl_2_	60	24 h	11
10	(CuOTf)_2_·toluene (0.3)	Toluene	80	2 h	100
11^c^^,^^d^	(CuOTf)_2_·toluene (0.3)	Toluene	80	20 min	100 (95)^b^
12^f^	CuCl (0.1)	DCE	70	17 h	0

a Reaction conditions: in a sealed tube, 1a (0.5 mmol), Ph_2_I^+^, ^−^OTf (2 equiv.), Cs_2_CO_3_ (2 equiv.) in solvent (3 mL).

b Isolated yields.

c Monitored by GC-MS.

d Gram-scale conditions: 1a (14 mmol, 2.07 g), 2a (30.8 mmol, 13.12 g), Cs_2_CO_3_ (28 mmol, 9.12 g) in toluene (40 mL).

e Isolated yield after purification by column chromatography.

f 1a (1.2 mmol), 2a (1.0 mmol), CuCl (10 mol%), H_2_O (1.15 mmol), DCE (10.0 mL) under argon at 70 °C for 17 h in a sealed tube.^12^ DMC: dimethylcarbonate.

**Scheme 2:**
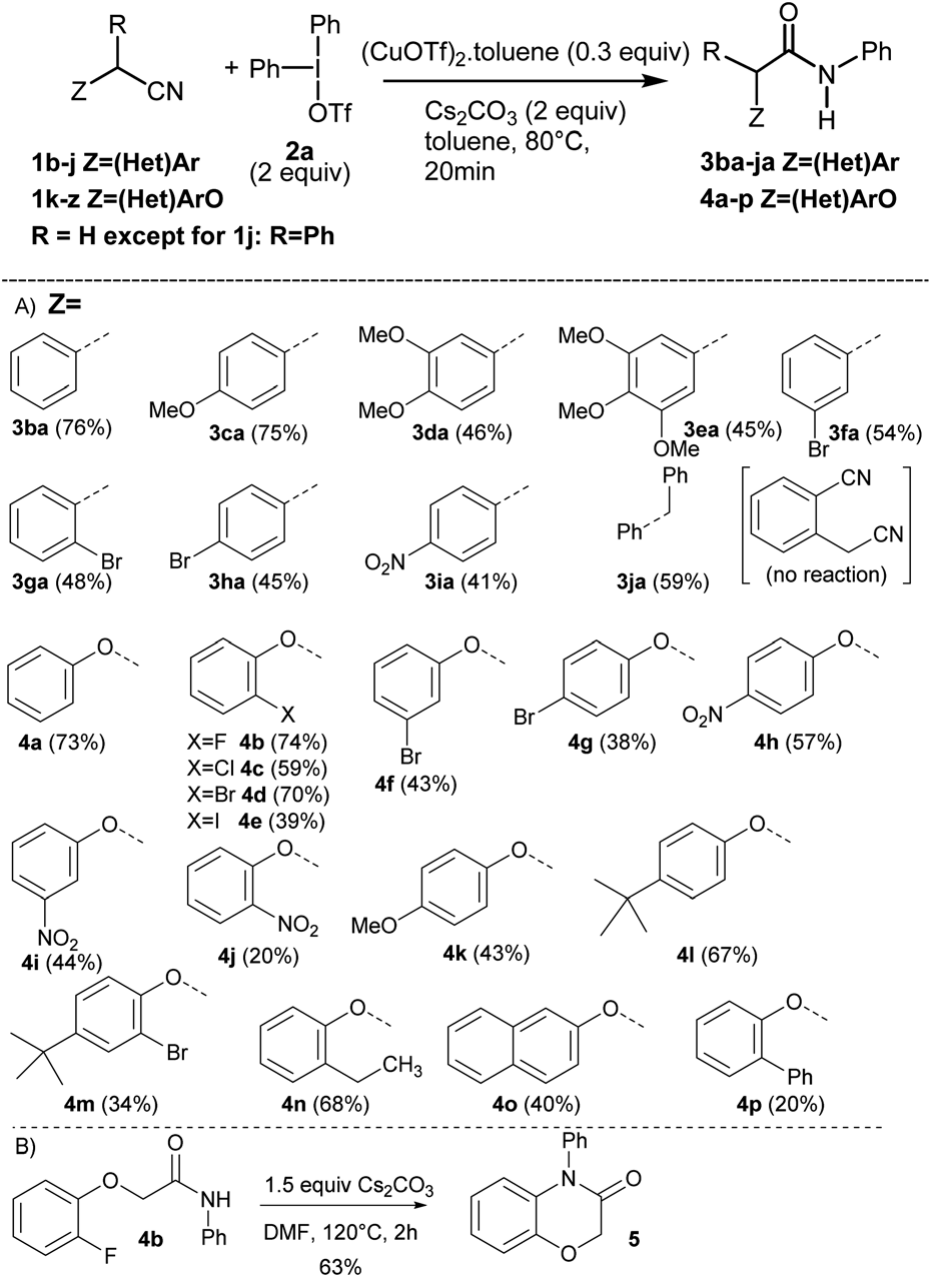
(A) Scope of the Cu-catalyzed *N*-arylation reaction using substituted alkyl- or benzyl nitriles 1b–z. (B) Smiles rearrangement from 4b.

Having identified the optimal reaction conditions (Table 1, entry 11), we next investigated the generality of this protocol. The reaction proceeded smoothly with a wide functional group tolerance. As shown in Scheme 2(A), various substituted benzyl nitriles 1b–j or alkyloxynitriles 1k-z participated in the reaction leading respectively to *N*-arylacetamides 3ba–ja or 4a–p in good to excellent yields. Benzylnitriles bearing either electron-donating groups (1c–e) or electron-withdrawing groups (1f–i), with different aryl substitution patterns were compatible with standard conditions, affording the desired products 3ca–ia in good yields. Halide substituents such as Br were particularly well tolerated, forming 3fa–3ha, which can be further functionalized *via* cross-coupling reactions. It is worth noting that the influence of the aryl substitution pattern was not evidenced. The reaction turned out to be compatible with tertiary alkyl nitriles such as diphenylacetonitrile 1j, but quaternary alkyl nitriles, such as the commercially available 2-methyl-2-phenylpropanenitrile, proved to be unsuccessful. Furthermore, it should be noted that this copper-catalyzed *N*-arylation reaction did not allow the transformation of 2-cyanophenylacetonitrile. Similarly, phenoxyacetonitriles 1k–z afforded the corresponding *N*-arylacetamides 4a–p with moderate to very good yields. The benefits of our approach lie both in the diversity offered by the initial choice of the functionalized phenol derivative, precursor of 1k–z or aryliodonium salt, and mild reaction conditions. Substrates bearing a pharmaceutically important fluorine atom (4b) and a naphthyl moiety (4o) were amenable in this reaction. In addition, conventional palladium cross-coupling reactions may be performed from the bromoaryl moiety in 4m. The hindered ([1,1′-biphenyl]-2-yloxy)acetonitrile 1z was also tolerated, yielding 4p albeit in a low yield.^18^ In order to demonstrate the value of the methodology developed, with the 2-fluoroaryloxymethylamide 4b in hand, the synthesis of 2*H*-1,4-benzoxazin-3-(4*H*)-ones, a privileged structure in the arena of pharmaceutical and agrochemical products was investigated (Scheme 2(B)).^19^ We sought to take advantage of the Smiles rearrangement^20^ in presence of Cs_2_CO_3_ as a base in DMF at 120 °C for 2 h. Consequently, the targeted 2*H*-1,4-benzoxazin-3-(4*H*)-one 5 was isolated in 63% yield. It is worth noting that diversity can be introduced on aryl substituents of 5.

**Scheme 3:**
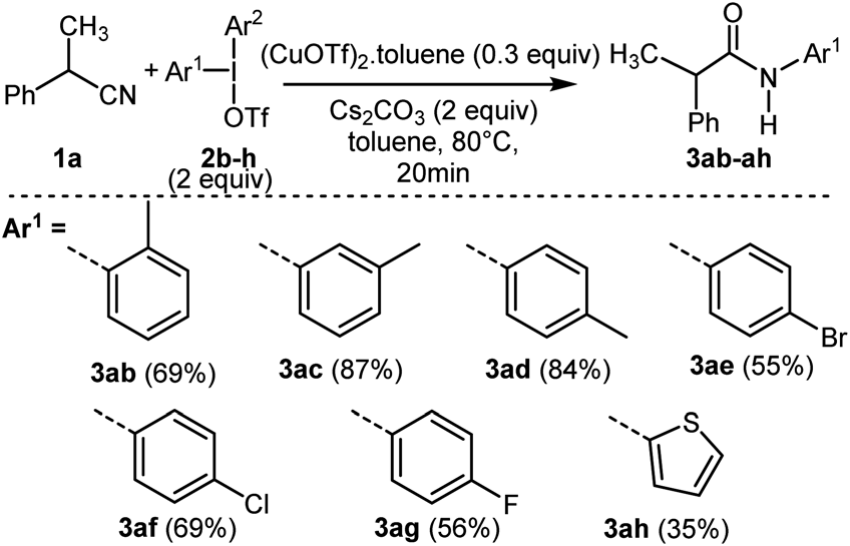
Scope of the reaction using symmetrical (2f–g) or unsymmetrical (2b–e, 2h) diaryliodonium salts.

Thereafter, the reactivity of various symmetrical (2f–g) and unsymmetrical diaryliodonium salts^13,21^ (2b–e with Ar^2^ = 2,4,6-trimethylphenyl, 2h with Ar^2^ = phenyl) was studied from 1a leading to new *N*-arylacetamides 3ab–ah with good yields (Scheme 3 and ESI†). As previously reported with unsymmetrical diaryliodonium salts in metal-catalyzed conditions, the less bulky aryl group was transferred more readily than the bulky one. Moderate yield was also obtained with a thienyl group allowing access to the heterocyclic product 3ah.

**Scheme 4:**
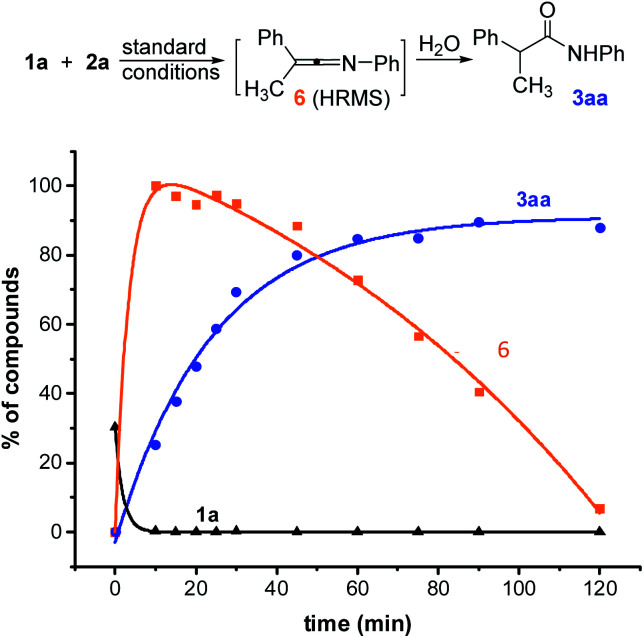
Time course experiments of Cu-catalyzed *N*-arylation of 1a (LC-HRMS). HPLC yield accounting for the response factor of 1a (black curve), 3aa (blue curve) and 6 (orange curve).

**Scheme 5:**
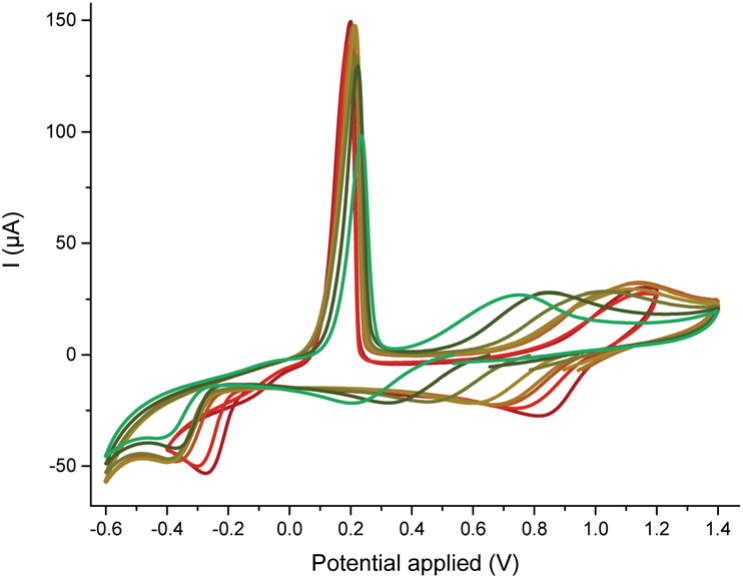
CV towards reduction (top) and oxidation (bottom) potentials of Cu^II^(OTf)_2_ (1 mM) in the presence of MeCN (158 equiv.) with increasing amounts of cyclohexylformamide (0, 1, 2, 5, 14, 50, 158 equiv. from red to green), recorded at a steady glassy carbon disk electrode (*d* = 3 mm) in nitromethane containing *n*-Bu_4_NBF_4_ (0.3 M) at 20 °C with a scan rate of 0.5 V s^−1^.

In order to better understand this reaction, we started mechanistic studies. HRMS analyses performed during the reaction of 1a with 2a under standard conditions revealed the formation of the ketenimine 6 within a few minutes (Scheme 4 and ESI, §VI†). Conversely, the *N*-arylacetamide 3aa probably formed due to a slow hydrolysis of the ketenimine intermediate 6 under basic conditions. When adding a stoichiometric amount of final acetamide at the beginning of the reaction, no evolution could be detected consistently with an inhibition of the catalyst by the product (see ESI, §VI†) (Scheme 5).

To investigate the competition between the substrate and the product with respect to copper, we resorted to cyclic voltammetry (CV) of a nitromethane solution of Cu^II^(OTf)_2_. The interaction of both Cu^II^ and electrogenerated Cu^I^ with a ligand could be assessed by this method, while the low coordinating ability of nitromethane avoided any binding competition issues.^22^ MeCN selected as a model substrate^23^ displayed only a weak affinity for Cu^II^ but proved to stabilize Cu^I^ due to the formation of a mixture of [Cu^I^(NCMe)_2_]^+^c2 and [Cu^I^(NCMe)_3_]^+^c3 – the latter being favoured at high MeCN concentration (see the ESI, §IV†).^24^ This result was confirmed by DFT calculations:^25^ while the formation of c3 is predicted to be the most exergonic process, both the formation of c2 (formation energy of 4.8 kcal mol^−1^ higher) and c4 (formation energy of only 1.1 kcal mol^−1^ higher) may be accessible as a function of the MeCN concentration (see ESI, §V†). A base is required for the reaction to proceed but the best one was Cs_2_CO_3_, which is poorly soluble in toluene. Thus, the concentration of hydroxides should be kept very low. The impact of hydroxides on the nature of the catalyst was tedious to evaluate experimentally, as copper salts tend to precipitate in the presence of hydroxides. To identify the possible species in the presence of hydroxides, we resorted to DFT calculations.

**Scheme 6:**
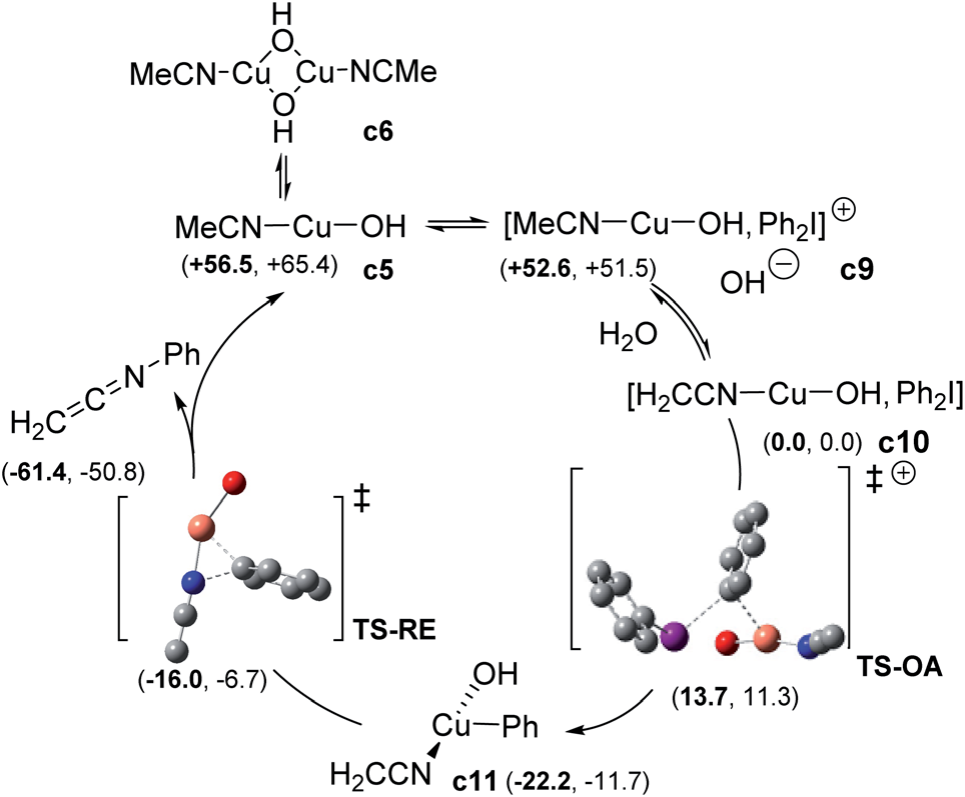
Computed oxidative addition-reductive elimination pathway (enthalpy and free energy (bold) are reported in kcal mol^−1^).

For the sake of completeness, we considered the possibility of forming either monomeric or dimeric Cu^I^ complexes with different ligand stoichiometries (See ESI, §V†). In all the cases, dimeric copper species were found to be more favourable than the corresponding hydroxo monometallic complex. The two hydroxo-bridged complexes [Cu^I^(OH)(MeCN)]_2_c6 and [Cu^I^(OH)(PhNHCOMe)]_2_c8 were the most relevant species. c8 turned out to be more stable than c6, due to the stabilizing effect of two intramolecular hydrogen bonds (see ESI, §V†). Consistently with the observed inhibition of the catalyst, ligand exchange to regenerate the active c6 complex was computed as not favorable. This finding could, at least partly, explain the high catalyst loading required for the reaction to proceed. Two possible mechanisms for the reaction of diaryliodonium with Cu^I^ complexes were next investigated: (i) the SET pathway and (ii) a two-electron transfer (oxidative addition, OA) from Cu^I^ to ArI_2_^+^.^26^ The addition of 3 equiv. of benzophenone or BHT did not affect the process, which ruled out a SET mechanism. The OA path was therefore calculated as the reaction can be promoted by different Cu^I^ sources (Scheme 6 and ESI†). The monomeric [Cu^I^(OH)(NCMe)] c5 can form an adduct (c9) with Ph_2_I^+^ and its formation is exergonic (−3.9 kcal mol^−1^). Deprotonation of c9 to form complex c10 can spontaneously occur (ΔG ≈ −50 kcal mol^−1^). Starting from c10, OA *via*TS-OA was accessible with a low barrier (+13.7 kcal mol^−1^) giving complex c11. The direct OA (TS-OA-bis) of c9 was less favourable, with an activation free energy of 24.4 kcal mol^−1^. Reductive elimination through a distorted T-shape transition state TS-RE takes place with an energy barrier of only 5.8 kcal mol^−1^ and coordination of a new nitrile allowed to regenerate c5 along with the experimentally observed ketenimine intermediate H_2_C=C=NPh.

## Conclusions

In summary, we have developed a new, simple and practical method for the copper-catalyzed synthesis of *N*-arylacetamide from easily accessible alkyl or benzyl nitrile. The protocol uses diaryliodonium salts as the electrophilic coupling partners. Mechanistic studies proved the formation of an intermediate ketenimine that is slowly hydrolyzed under the reaction conditions. CV experiments demonstrated the high affinity of the product for the catalyst, justifying the catalyst loading required. Finally, DFT calculations ascertained that a two-electron activation *i.e.* oxidative addition is energetically possible. Efforts to expand the utility of the method are in progress in our laboratory.

## Conflicts of interest

There are no conflicts to declare.

## Supplementary Material

RA-011-D1RA02305E-s001
