# Supplementary material for: Copper-catalyzed transformation of alkyl nitriles to N-arylacetamide using diaryliodonium salts
Source: RSC Adv. 2021 Apr 28;11(26):15885–9. doi: 10.1039/d1ra02305e (PMC9036411; doi:10.1039/d1ra02305e)

Electronic Supplementary Material (ESI) for RSC Advances.
This journal is © The Royal Society of Chemistry 2021

Supporting Information

Copper-Catalyzed Transformation of Alkyl Nitriles to *N*-Arylacetamide using Diaryliodonium Salts

Romain Sallio,[a] Pierre-Adrien Payard,[b] Paweł Pakulski,[a] Iryna Diachenko,[a] Indira Fabre,[b] Sabine Berteina-Raboin,[a] Cyril Colas, [a] Ilaria Ciofini,[c] Laurence Grimaud*[b] and Isabelle Gillaizeau*[a]

TABLE OF CONTENT.

GENERAL REMARKS. S3

EXPERIMENTAL PROCEDURES AND CHARACTERIZATION DATA. S3

I. General procedure for the preparation of aryloxyacetonitriles **1k-z** S3

II. General procedure for *N*-arylation of nitriles **1** (**GP1**) S4

-> Experimental procedures and characterization data for *N*-arylacetamides **3** and **4** S4

III. Synthesis of 4-phenyl-2H-benzo[b][1,4]oxazin-3(4H)-one (**5**) S13

IV. CV Experiments S14

V. DFT Calculations S16

VI. Kinetics S19

VII. Computational details and Cartesian coordinates S21

COPIES OF ^{1}H AND ^{13}C NMR SPECTRA OF COMPOUNDS **3**, **4**, **5, 6**, **7** S39

GENERAL REMARKS.

All reagents and solvents were purchased from commercial sources unless otherwise stated. Anhydrous solvents were dried according to standard procedures. Dry glassware for moisture-sensitive was obtained by oven-drying. Most of the reactions were carried out under argon as an inert atmosphere. The reactions were monitored by thin-layer chromatography (TLC) analysis using silica gel (60 F254) plates. Compounds were visualized by UV irradiation and/or spraying with a solution of potassium permanganate, followed by charring at 150 °C. Flash column chromatography was performed on silica gel 60 (230-400 mesh, 0.040-0.063 mm). ^{1}H and ^{13}C NMR spectra were recorded on a spectrometer at 250 MHz (^{13}C, 62.9 MHz) or 400 MHz (^{13}C, 101 MHz). Chemicals shifts are given in parts per million from tetramethylsilane (TMS) as an internal standard. The following abbreviations are used for the proton spectra multiplicities: s.: singlet, d.: doublet, t.: triplet, m.: multiplet, br.: broad. Coupling constants (J) are reported in hertz (Hz). High-resolution accurate mass measurements (HRAM) were performed in positive mode with an ESI source on a Q-TOF mass spectrometer with an accuracy tolerance of 2 ppm by the "Federation de Recherche" ICOA/CBM platform. The IUPAC name of the new compounds was generated automatically using the included structure-to-name generator from BIOVIA Draw 201.

EXPERIMENTAL PROCEDURES AND CHARACTERIZATION DATA.

Nitriles **1a-1j 1z**, **5**, copper sources and iodonium triflates **2a** are commercially available and were used as received. Nitriles 1j,[1] 1k,[2] 1l,[3] 1m,[4] 1n,[5] 1o,[6] 1p,[1] 1q,[2] 1r,[3] 1s,[7] 1t,[2] 1u,[3] 1v,[3] 1w,[3] 1x,[8] 1y,[9] 1z[10] (cf. the general procedure pS3) and iodonium triflate 2b-h[11] were prepared as previously reported.

I. General procedure for the preparation of aryloxyacetonitriles 1k-z. Bromoacetonitrile (0.4ml; 5.7 mmol) and phenol derivative (5 mmol) were placed in a 25ml round-bottomed flask equipped with magnetic stirrer and dissolved in 8 ml of acetone. Oven-dried K_2CO_3 (1.022g, 7.4 mmol) was added, and the mixture was stirred overnight at room temperature. Then it was filtrated through cotton pad, solids were washed with 10ml of ethyl acetate. The organic phase was washed with 10ml of brine, 10ml of 1N aqueous solution NaOH, again with brine and dried over anhydrous $MgSO_4$. Solvent was removed on rotary evaporator. Residue was passed through silica pad using eluent: petroleum ether/AcOEt (7:1) to afford pure aryloxyacetonitrile **1**. NMR data correspond to the reported values.

---

1. Garazd, M. M.; Garazd, Y. L.; Ogorodniichuk, A. S.; Shilin, V.V.; Turov, A. V.; Khilya, V. P. *Chem. Nat. Compd.* **1998**, *34*, 442–447.
2. Vasilyev, S. A.; Boyarchuk, V. L.; Lukyanchikov, M. S.; Khilya, V.P. *Khim.-Farm. Zh.* **1991**, *25*, 50–55.
3. Yu, G.; Kuo, D.; Shoham, M.; Viswanathan, R. *ACS Comb. Sci.* **2014,** 16, 85–91.
4. Deng, J.-H.; Tai, H.-M.; Yang, C.-C. *J. Chin. Chem. Soc.* **2000**, *47*, 327–341.
5. Manasieva, L. I.; Maria, B. U. ; Prandi, A.; Brasili, L.; Franchini, S. *Synthesis* **2015**, 3767-3775.
6. Portscheller, J. L.; Malinakova, H. C. *Org. Lett.* **2002**, *4*, 3679–3681.
7. Bergeron, P.; Koehler, M. F. T.; Blackwood, E. M.; Bowman, K.; Clark, K.; Firestein, R.; Kiefer, J. R.; Wu, J.; Beresini, M. *ACS Med. Chem. Lett.*, **2016**, *7*, 595-600.
8. Carter, D.S.; Alam, M.; Cai, H.; Dillon, M.P.; Ford, A.P.D.W.; Gever, J. R.; Jahangir, A.; Wagner, P. J.; Zhai, Y. *Bioorg. Med. Chem. Lett.*, **2009**, *19*, 1628-1631.
9. Barkin, J. L.; Faust Jr., M. D.; Trenkle, W. C. *Org. Lett.,* **2003**, *5*, 3333-3335.
10. Shin, H.-S.; Kim, S.; Myung, S.-W.; Park, J.-S. *Anal. Chem*. **1995**, 67, 1853 – 1859.
11. (a) Kuriyama, M. ; Hamaguchi, N. ; Onomura, O. *Chem. Eur. J.,* **2012***, 18,* 1591-1594. (b) Bielawski, M.; Aili, D.; Olofsson, B. *J. Org. Chem*. **2008,** 73, 4602-4607.

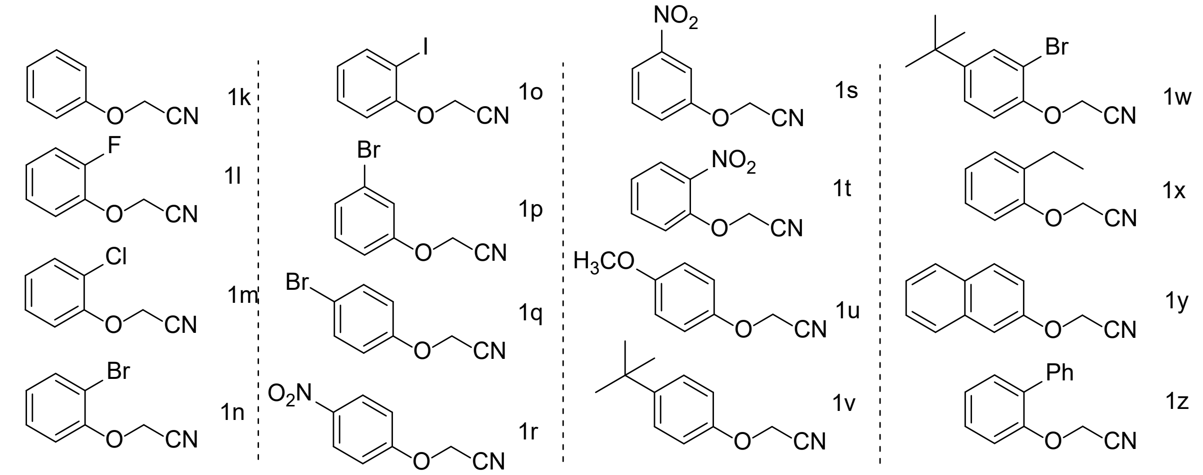

II. General procedure for N-arylation of nitriles 1 (GP1)

A sealed tube was charged with nitrile **1** (0.5 mmol, 1 equiv), aryliodonium salt **2** (1 mmol, 2 equiv), Cs_2CO_3 (1 mmol, 2 equiv) and copper (I) trifluoromethanesulfonate toluene complex (0.15 mmol, 0.3 equiv) in toluene (3 mL). The reaction tube was degassed and refilled with argon three times, and then the mixture was stirred at 80 °C and monitored by TLC. Upon completion of the reaction, saturated aqueous solution of $NaHCO_3$ (10 mL) was added and the aqueous layer was extracted with CH_2Cl_2 (3 x 10 mL). The organic layer was washed with brine, dried over $MgSO_4$ and evaporated to dryness. The resulting crude product was purified through silica gel column chromatography using petroleum ether (EP)/EtOAc as eluent affording *N*-aryl amides **3** or **4**.

Experimental procedures and characterization data for N-arylacetamides 3 and 4

N-2-Diphenylpropanamide (**3aa**)

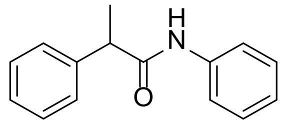

The compound **3aa** was prepared according to the general procedure (**GP1**) starting from α-methylphenylacetonitrile **1a** and diphenyliodonium triflate **2a**. Purification by flash chromatography using PE/EA (9:1, v/v) afforded **3aa** as a yellow solid (100% yield, 113 mg); m.p. 133-134 °C; ^{1}H (400 MHz, $CDCl_3$): δ 7.42-7.23 (m, 9H), 7.10-6.97 (m, 2H), 3.71 (q, *J* = 7.1 Hz, 1H), 1.59 (d, *J* = 7.2 Hz, 3H); ^{13}C (101 MHz, $CDCl_3$): δ 172.3, 141.0, 137.9, 129.3, 129.0, 127.8, 127.7, 124.3, 119.8, 48.3, 18.7; HRMS (ESI+): calcd for $C_{15}H_{16}NO^+$ $[M+H]^+$ 226.1226, found 226.1227. NMR data correspond to the reported values.[12]

N-2-Diphenylacetamide (**3ba**)

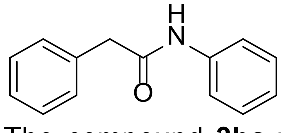

The compound **3ba** was prepared according to the general procedure (**GP1**) starting from phenylacetonitrile **1b** and diphenyliodonium triflate **2a**. Purification by flash chromatography using PE/EA (9:1, v/v) afforded **3ba** as a white solid in 76% yield, 80 mg; m.p. 114-115 °C; ^{1}H (400 MHz, $CDCl_3$): δ 7.46-7.24 (m, 9H), 7.11-7.05 (m, 2H), 3.77 (s, 2H); ^{13}C (101 MHz, $CDCl_3$): δ 165.3, 137.7, 134.5, 129.6 (2xC), 129.4, 129.0 (2xC), 127.8, 124.6, 119.9 (2xC), 45.0; HRMS

12. Ye, W. ; Mo, J. ; Zhaoa, T. ; Xu, B. *Chem. Commun.*, **2009**, 3246-3248.

(ESI+): calcd for $C_{14}H_{14}NO^+$ $[M+H]^+$ 212.1069, found 212.1069. NMR data correspond to the reported values.[13]

2-(4-Methoxyphenyl)-*N*-phenylacetamide (**3ca**)[14]

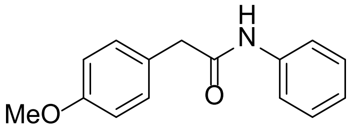

The compound **3ca** was prepared according to the general procedure (**GP1**) starting from 4-methoxyphenylacetonitrile **1c** and diphenyliodonium triflate **2a**. Purification by flash chromatography using PE/EA (9:1, v/v) afforded **3ca** as an orange solid in 75% yield, 90 mg; m.p. 93-95 °C; ^{1}H (400 MHz, $CDCl_3$): δ 7.43-7.39 (d, *J* = 7.6 Hz, 2H), 7.27-7.19 (m, 5H), 7.05 (t, *J* = 7.6 Hz, 1H), 6.90-6.86 (d, *J* = 8.4 Hz, 2H), 3.78 (s, 3H), 3.62 (s, 2H); ^{13}C (101 MHz, $CDCl_3$): δ 169.8, 159.1, 137.8, 130.6, 129.5, 128.9, 128.0, 127.2, 126.6, 124.3, 119.9, 114.6, 111.8, 55.7, 43.6; HRMS (ESI+): calcd for $C_{15}H_{16}NO_2^+$ $[M+H]^+$ 242.1175, found 242.1174.

2-(3,4-Dimethoxyphenyl)-*N*-phenylacetamide (**3da**)

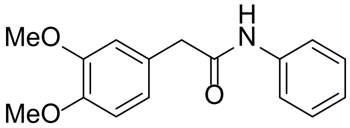

The compound **3da** was prepared according to the general procedure (**GP1**) starting from (3,4-dimethoxyphenyl)acetonitrile **1d** and diphenyliodonium triflate **2a**. Purification by flash chromatography using PE/EA (9:1, v/v) afforded **3da** as a brown solid in 46% yield, 56 mg; m.p. 92-94 °C; ^{1}H (400 MHz, $CDCl_3$): δ 7.49-7.44 (m, 3H), 7.31-7.27 (m, 2H), 7.11-7.07 (m, 1H), 6.88-6.85 (m, 3H), 3.90 (s, 3H), 3.87 (s, 3H), 3.67 (s, 2H); ^{13}C (101 MHz, $CDCl_3$): δ 169.6, 149.4, 148.5, 137.7, 128.9 (2xC), 126.9, 124.4, 121.7, 119.8 (2xC), 112.5, 111.6, 55.9 (2xCH_3), 44.4; HRMS (ESI+): calcd for $C_{16}H_{18}NO_3^+$ $[M+H]^+$ 272.1281, found 272.1280.

N-Phenyl-2-(3,4,5-trimethoxyphenyl)acetamide (**3ea**)

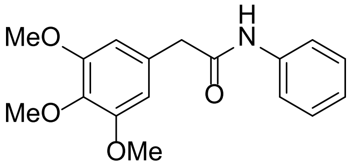

The compound **3ea** was prepared according to the general procedure (**GP1**) starting from (3,4,5-trimethoxyphenyl)acetonitrile **1d** and diphenyliodonium triflate **2a**. Purification by flash chromatography using PE/EA (9:1, v/v) afforded **3ea** as a grey solid in 45% yield, 65 mg; m.p. 152-155 °C; ^{1}H (400 MHz, $CDCl_3$): δ 7.63 (brs, 1H), 7.49-7.47 (m, 2H), 7.32-7.28 (m, 2H), 7.12-7.08 (m, 1H), 6.55 (brs, 2H), 3.86 (s, 3H), 3.85 (s, 6H), 3.66 (s, 2H); ^{13}C (101 MHz, $CDCl_3$): δ 169.3, 153.6 (2xC), 137.7, 137.3, 130.1, 128.9 (2xC), 124.5, 119.9 (2xC), 106.4 (2xC), 60.9, 56.1 (2xC), 44.9; HRMS (ESI+): calcd for $C_{17}H_{20}NO_4^+$ $[M+H]^+$ 302.1387, found 302.1386.

2-(3-Bromophenyl)-*N*-phenylacetamide (**3fa**)

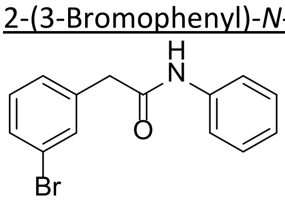

13. Nordstrøm, L. U.; Vogt, H.; Madsen, R. *J. Am. Chem. Soc.*, **2008**, *130,* 17672-17673.
14. (a) Chirila, A. ; van Vliet, K. M. ; Paul, N. D. ; de Bruin, B. *Eur. J. Inorg. Chem.*, **2018**, 20-21, 2251-2258. (b) Yu, W. Yang, S.; Xiong, F.; Fan, T.; Feng, Y. Huang, Y. Fu, J. Wang, T. *Org. Biomol. Chem.*, **2018**, *16*, 3099-3103.

The compound **3fa** was prepared according to the general procedure (**GP1**) starting from (3-bromophenyl)acetonitrile **1e** and diphenyliodonium triflate **2a**. Purification by flash chromatography using PE/EA (9:1, v/v) afforded **3fa** as a yellow solid in 54% yield, 78 mg; m.p. 139-140 °C; ^{1}H NMR (400 MHz, DMSO-d6) δ 10.18 (brs, 1H), 7.62-7.55 (m, 3H), 7.46-7.43 (m, 1H), 7.33-7.25 (m, 4H), 7.07-7.00 (m, 1H), 3.60 (s, 2H); ^{13}C (100 MHz, DMSO-d6): δ 169.9, 139.2, 139.1, 132.4, 131.2, 130.3, 129.6, 128.9, 124.6, 122.3, 120.2, 43.3; HRMS (ESI+): calcd for $C_{14}H_{13}BrNO^+$ $[M+H]^+$ 290.0175, found 290.0175.

2-(2-Bromophenyl)-*N*-phenylacetamide (**3ga**)

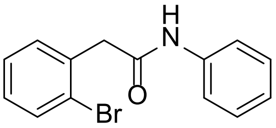

The compound **3ga** was prepared according to the general procedure (**GP1**) starting from (2-bromophenyl)acetonitrile **1g** and diphenyliodonium triflate **2a**. Purification by flash chromatography using PE/EA (9:1, v/v) afforded **3ga** as a yellow solid in 48% yield, 70 mg; m.p. 144-145 °C; ^{1}H (400 MHz, $CDCl_3$): δ 7.64-7.62 (m, 1H), 7.60-7.25 (m, 1H), 7.49-7.41 (m, 2H), 7.43-7.41 (m, 1H), 7.36-7.28 (m, 3H), 7.22-7.18 (m, 1H), 7.13-7.09 (m, 1H), 3.87 (s, 2H); ^{13}C (101 MHz, $CDCl_3$): δ 168.0, 137.7, 134.6, 133.2, 131.8, 129.4, 129.0, 128.1, 125.0, 124.5, 119.7, 77.4, 77.1, 76.8, 44.9; HRMS (ESI+): calcd for $C_{14}H_{13}BrNO^+$ $[M+H]^+$ 290.0175, found 290.0174.
NMR data correspond to the reported values.[15]

2-(4-Bromophenyl)-*N*-phenylacetamide (**3ha**)

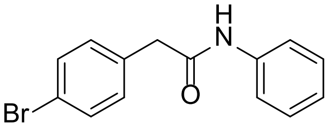

The compound **3ha** was prepared according to the general procedure (**GP1**) starting from (4-bromophenyl)acetonitrile **1h** and diphenyliodonium triflate **2a**. Purification by flash chromatography using PE/EA (9:1, v/v) afforded **3ha** as a yellow solid in 45% yield, 62 mg; m.p. 140-141 °C; ^{1}H NMR (250 MHz, DMSO) δ 10.16 (brs, 1H), 7.62-7.56 (m, 2H), 7.54-7.50 (m, 2H), 7.34-7.25 (m, 4H), 7.07-6.99 (m, 1H), 3.63 (s, 2H); HRMS (ESI+): calcd for $C_{14}H_{13}BrNO^+$ $[M+H]^+$ 290.0175, found 290.0173. NMR data correspond to the reported values.[16]

4-Nitrophenylacetanilide (**3ia**)

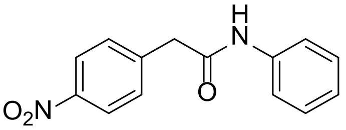

The compound **3ia** was prepared according to the general procedure (**GP1**) starting from (4-nitrophenyl)acetonitrile **1i** and diphenyliodonium triflate **2a**. Purification by flash chromatography using PE/EA (9:1, v/v) afforded **3ia** as a yellow solid in 41% yield, 53 mg; m.p. 206-208 °C; ^{1}H (400 MHz, $(CD_3)_2CO$): δ 9.45 (brs, 1H), 8.22 (d, *J* = 8 Hz, 2H), 7.67 (t, *J* = 8 Hz, 4H), 7.31 (t, *J* = 8 Hz, 2H), 7.07 (t, *J* = 6 Hz, 1H), 3.92 (s, 2H); ^{13}C (101 MHz, $(CD_3)_2CO$): δ 167.6, 143.8, 139.3, 130.5 (2xC), 128.7 (2xC), 126.8, 123.5, 123.2 (2xC), 119.3 (2xC), 43.2;

15. Sun, J.; Tan, Q.; Yang, W.; Liu, B.; Xu, B. *Adv. Synth. Catal.*,**2014**,*356*, 388–394.
16. (a) Ogiwara, Y.; Shimoda, W.; Ide, K.; Nakajima, T.; Sakai, N. *Eur. J. Org. Chem.*, **2017**, *20*, 2866-2870. (b) Ali Molla, R.; A. Iqubal,A; Ghosh, K.; Islam, M. *Dalton Trans.*, **2015**, *44*, 6546-6559. (c) X. Li, Z. Li, H. Deng, X. Zhou, *Tet. Lett.* **2013**, *54*, 2212-2216.

HRMS (ESI+): calcd for $C_{14}H_{13}N_2O_3^+$ [M+H]$^+$ 257.0921, found 257.0918. NMR data correspond to the reported values.[17]

N,2,2-Triphenylacetamide (**3ja**)

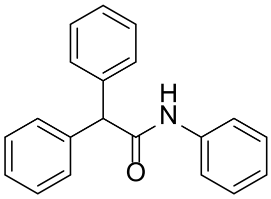

The compound **3ja** was prepared according to the general procedure (**GP1**) starting from diphenylacetonitrile **1j** and diphenyliodonium triflate **2a**. Purification by flash chromatography using PE/EA (9:1, v/v) afforded **3ja** as a yellow solid in 59% yield, 85 mg; m.p. 181-182 °C; ^{1}H (400 MHz, $CDCl_3$): δ 7.66-7.10 (m, 16H), 5.11 (s, 1H); ^{13}C (101 MHz, $CDCl_3$): δ 170.3, 139.1 (2xC), 137.7, 128.9 (6 x C), 127.5 (2xC), 124.6, 119.9 (2xC), 59.9; HRMS (ESI+): calcd for $C_{20}H_{18}NO^+$ [M+H]$^+$ 288.1383, found 288.1383. NMR data correspond to the reported values.[18]

2-Phenyl-*N*-*o*-tolylpropanamide (**3ab**)

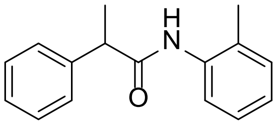

The compound **3ab** was prepared according to the general procedure (**GP1**) starting from nitrile **1a** and (2-methylphenyl)(2,4,6-trimethylphenyl)iodonium triflate **2b**. Purification by flash chromatography using PE/EA (9:1, v/v) afforded **3ab** as a yellow solid (69% yield, 83 mg); m.p. 82-83 °C; ^{1}H (400 MHz, $CDCl_3$): δ 7.81 (m, 1H), 7.43-6.98 (m, 9H), 3.80 (q, *J* = 7.2 Hz, 1H), 1.93 (s, 3H), 1.67 (d, *J* = 7.2 Hz, 3H); ^{13}C (101 MHz, $CDCl_3$): δ 172.4, 141.1, 136.7, 130.3, 129.2, 128.6, 127.8 (2xC), 127.7, 126.7 (2xC), 124.9, 122.5, 47.9, 18.1, 17.1; HRMS (ESI+): calcd for $C_{16}H_{18}NO^+$ [M+H]$^+$ 240.1382, found 240.1381. NMR data correspond to the reported values.[19]

2-Phenyl-*N*-*m*-tolylpropanamide (**3ac**)

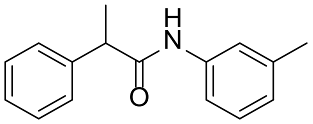

The compound **3ac** was prepared according to the general procedure (**GP1**) starting from nitrile **1a** and (3-methylphenyl)(2,4,6-trimethylphenyl)iodonium triflate **2c**. Purification by flash chromatography using PE/EA (9:1, v/v) afforded **3ac** as a yellow oil in 87% yield, 104 mg; ^{1}H (400 MHz, $CDCl_3$): δ 7.42-7.31 (m, 7H), 7.25 (d, *J* = 8 Hz, 1H), 7.17-7.09 (m,1H), 6.87-6.85 (m, 1H), 3.70 (q, *J* = 8 Hz, 1H), 2.25 (m, 3H), 1.58-1.55 (m, 3H); ^{13}C (101 MHz, $CDCl_3$): δ 172.5, 142.8, 142.4, 142.0, 139.1, 138.8, 129.1 (2xC), 128.7, 127.7 (2xC), 127.5, 125.1, 120.5, 116.9, 47.3, 21.4, 18.6; HRMS (ESI+): calcd for $C_{16}H_{18}NO^+$ [M+H]$^+$ 240.1383, found 240.1382. NMR data correspond to the reported values.[19]

2-Phenyl-*N*-*p*-tolylpropanamide (**3ad**)

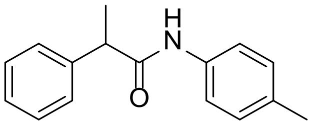

17. Dong, Y.; Liu, B.; Chen, P.; Liu, Q.; Wang, M. *Angew. Chem., Int. Ed.*, 2**014,** *53*, 3442-3446.
18. Zhu, Y.-P.; Sergeyev, S.; Franck, P.; Orru, R. V. A.; Maes, B.U. W. *Org. Lett.*, **2016**, *18*, 4602-4605.
19. Ye, W.; Mo, J.; Zhao, T.; Xu, B. *Chem. Commun.*, **2009**, *22*, 3246-3248.

The compound **3ad** was prepared according to the general procedure (**GP1**) starting from nitrile **1a** and (4-methylphenyl)(2,4,6-trimethylphenyl)iodonium triflate **2d**. Purification by flash chromatography using PE/EA (9:1, v/v) afforded **3ad** as a white solid in 84% yield, 101 mg; m.p. 116-117 °C; ^{1}H (400 MHz, $CDCl_3$): δ 7.40-7.35 (m, 4H), 7.32-7.26 (m, 3H), 7.07 (d, *J* = 8 Hz, 2H), 6.94 (brs, 1H), 3.72 (q, *J* = 8 Hz, 1H), 2.30 (s, 3H), 1.61-1.59 (d, *J* = 8 Hz, 3H); ^{13}C (101 MHz, $CDCl_3$): δ 172.1, 141.0, 135.3, 133.9, 129.4 (2xC), 129.1 (2xC), 127.7 (2xC), 127.5, 119.7 (2xC), 48.1, 20.8, 18.6; HRMS (ESI+): calcd for $C_{16}H_{18}NO^+$ $[M+H]^+$ 240.1383, found 240.1379. NMR data correspond to the reported values.[19]

N-(4-Bromophenyl)-2-phenylpropanamide (**3ae**)

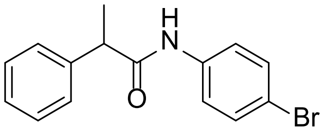

The compound **3ae** was prepared according to the general procedure (**GP1**) starting from nitrile **1a** and (4-bromophenyl)(2,4,6-trimethylphenyl)iodonium triflate **2e**. Purification by flash chromatography using PE/EA (9:1, v/v) afforded **3ae** as a yellow solid in 55% yield, 84 mg; m.p. 129-130 °C; ^{1}H (400 MHz, $CDCl_3$): δ 7.39-7.26 (m, 9H), 7.05 (brs, 1H), 3.72 (q, *J* = 7.1 Hz, 1H), 1.61 (d, *J* = 7.2 Hz, 3H); ^{13}C (101 MHz, $CDCl_3$): δ 172.4, 140.6, 136.9, 131.8 (2xC), 129.2 (2xC), 128.2 (2xC), 121.8, 117.7, 48.1, 17.6; HRMS (ESI+): calcd for $C_{15}H_{15}BrNO^+$ $[M+H]^+$ 304.0331, found 304.0334. NMR data correspond to the reported values.[19]

N-(4-Chlorophenyl)-2-phenylpropanamide (**3af**)

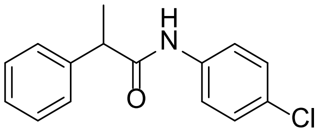

The compound **3af** was prepared according to the general procedure (**GP1**) starting from nitrile **1a** and bis(4-chlorophenyl)iodonium triflate **2f**. Purification by flash chromatography using PE/EA (9:1, v/v) afforded **3af** as a white solid in 69% yield, 90 mg; m.p. 132-133 °C; ^{1}H (400 MHz, $CDCl_3$): δ 7.38-7.19 (m, 10H), 3.69 (q, *J* = 8 Hz, 1H), 1.59 (d, *J* = 8 Hz, 3H); ^{13}C (101 MHz, $CDCl_3$): δ 172.5, 140.7, 136.4, 129.2 (2xC), 128.9 (2xC), 127.7, 127.6 (2xC), 121.0 (2xC), 116.8, 48.1, 18.6; HRMS (ESI+): calcd for $C_{15}H_{15}ClNO^+$ $[M+H]^+$ 260.0837, found 260.0838. NMR data correspond to the reported values[19]

N-(4-Fluorophenyl)-2-phenylpropanamide (**3ag**)

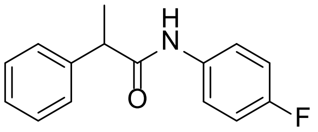

The compound **3ag** was prepared according to the general procedure (**GP1**) starting from nitrile **1a** and bis(4-fluorophenyl)iodonium triflate **2g**. Purification by flash chromatography using PE/EA (9:1, v/v) afforded **3ag** as a white solid in 56% yield, 68 mg; m.p. 95-96 °C; ^{1}H (400 MHz, $CDCl_3$): δ 7.63 (brs, 1H), 7.33-7.23 (m, 7H), 6.93-6.86 (m, 2H), 3.68 (q, *J* = 7.1 Hz, 1H), 1.54 (d, *J* = 7.1 Hz, 3H); ^{13}C (101 MHz, $CDCl_3$): δ 171.5, 159.3 (d, *J* = 243.6 Hz), 141.5, 133.5, 129.0 (2xC), 127.6, 127.5, 121.9 (d, *J* = 7.9 Hz), 115.4 (d, *J* = 22.4 Hz), 47.7, 18.5; HRMS (ESI+): calcd for $C_{15}H_{15}FNO^+$ $[M+H]^+$ 244.1132, found 244.1130. NMR data correspond to the reported values[19]

2-Phenyl-*N*-4-thien-2-yl-propanamide (**3ah**)

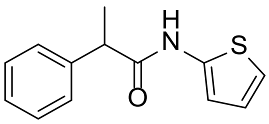

The compound **3ah** was prepared according to the general procedure (**GP1**) starting from nitrile **1a** and 2-thienyl-(phenyl)iodonium triflate **2h**. Purification by flash chromatography using PE/EA (9:1, v/v) afforded **3ah** as a white oil in 35% yield, 68 mg; ^{1}H (400 MHz, $CDCl_3$): δ 7.43-7.03 (m, 9H), 3.71 (m, 1H), 1.58 (d, *J* = 7.1 Hz, 3H); ^{13}C (101 MHz, $CDCl_3$): δ 172.3, 140.9, 137.8, 129.1, 128.9, 127.7, 127.5, 124.2, 119.7, 48.1, 18.5; HRMS (ESI+): calcd for $C_{13}H_{14}NOS$ $[M+H]^+$ 232,0796, found 232,0794. NMR data correspond to the reported values.[20]

2-Phenoxy-*N*-phenylacetamide (**4a**)

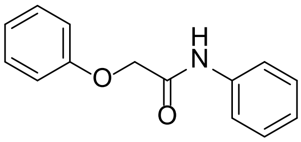

The compound **4a** was prepared according to the general procedure (**GP1**) starting from nitrile **1k** and diphenyliodonium triflate **2a.** Purification by flash chromatography using PE/EA (9:1, v/v) afforded **4a** as a white solid in 73% yield, 83 mg; m.p. 93-95 °C; ^{1}H (400 MHz, $CDCl_3$): δ 8.29 (brs, 1H), 7.57 (d, *J* = 7.5 Hz, 2H), 7.34 (t, *J* = 8.8 Hz, 4H) 7.17-6.96 (m, 4H), 4.58 (s, 2H); ^{13}C (101 MHz, $CDCl_3$): δ 166.2, 157.0, 136.8, 129.9 (2xC), 129.1 (2xC), 124.9, 122.5, 120.1 (2xC), 114.9 (2xC), 67.7; HRMS (ESI+): calcd for $C_{14}H_{14}NO_2^+$ $[M+H]^+$ 228.1019, found 228.1020. NMR data correspond to the reported values.[21]

2-(2-Fluorophenoxy)-*N*-phenylacetamide (**4b**)

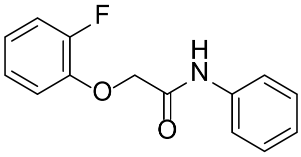

The compound **4b** was prepared according to the general procedure (**GP1**) starting from nitrile **1l** and diphenyliodonium triflate **2a.** Purification by flash chromatography using PE/EA (9:1, v/v) afforded **4b** as a white solid in 74% yield, 91 mg; m.p. 104-105 °C; ^{1}H (400 MHz, $CDCl_3$): δ 8.45 (brs, 1H), 7.59 (d, *J* = 7.5 Hz, 2H), 7.33 (t, *J* = 7.5 Hz, 2H), 7.16-6.96 (m, 5H), 4.61 (s, 2H); ^{13}C (101 MHz, $CDCl_3$): δ 165.7, 152.8 (d, *J* = 245.8 Hz), 145.3 (d, *J* = 10.9 Hz), 136.8, 129.1 (2xC), 124.9, 124.8 (d, *J* = 4 Hz), 123.3 (d, *J* = 7 Hz), 120.1 (2xC), 116.7 (d, *J* = 18.2 Hz), 116.2, 69.2; HRMS (ESI+): calcd for $C_{14}H_{13}FNO_2^+$ $[M+H]^+$ 246.0925, found 246.0923.

2-(2-Chlorophenoxy)-*N*-phenylacetamide (**4c**)

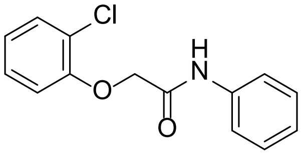

The compound **4c** was prepared according to the general procedure (**GP1**) starting from nitrile **1m** and diphenyliodonium triflate **2a.** Purification by flash chromatography using PE/EA (9:1, v/v) afforded **4c** as a white solid in 59% yield, 77 mg; m.p. 121-122 °C; ^{1}H (400 MHz, $CDCl_3$): δ 8.67 (brs, 1H), 7.62 (d, *J* = 7.6 Hz, 2H), 7.44 (dd, *J* = 7.9, 1.6 Hz, 1H), 7.37 (t, *J* = 8 Hz, 2H), 7.28 (td, *J* = 7.9, 1.6 Hz, 1H), 7.16 (t, *J* = 7.4 Hz, 1H), 7.02 (td, *J* = 7.7, 1.3 Hz, 1H), 6.97 (d, *J* = 8.2 Hz, 1H), 4.66 (s, 2H); ^{13}C (101 MHz, $CDCl_3$): δ 165.5, 152.6, 136.9, 130.5, 129.2 (2xC),

20. Li, F.; Shi, L.; Xia, C.; Yang, L. *Chin. J. Catal.*, **2020**, 41, 1152 - 1160
21. Nakatsuji, H.; M., Mami; M., Tomonori; T., Y. *Tetrahedron*, **2007**, *63*, 12071-12080.

128.3, 124.9, 123.2, 123.0, 119.9 (2xC), 114.2, 68.3; HRMS (ESI+): calcd for $C_{14}H_{13}ClNO_2^+$ $[M+H]^+$ 262.0629, found 262.0628.

2-(2-Bromophenoxy)-*N*-phenylacetamide (**4d**)

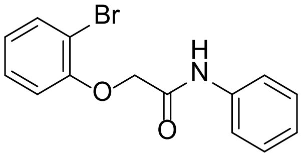

The compound **4d** was prepared according to the general procedure (**GP1**) starting from nitrile **1n** and diphenyliodonium triflate **2a.** Purification by flash chromatography using PE/EA (9:1, v/v) afforded **4d** as a white solid in 70% yield, 107 mg; m.p. 125-126 °C; ^{1}H (400 MHz, $CDCl_3$): δ 8.72 (brs, 1H), 7.64-7.56 (m, 3H), 7.37-7.26 (m, 3H), 7.13 (t, *J* = 7.5 Hz, 1H), 6.96-6.87 (m, 2H), 4.60 (s, 2H); ^{13}C (101 MHz, $CDCl_3$): δ 165.3, 153.4, 137.0, 133.5, 129.1 (2xC), 129.0, 124.8, 123.6, 119.9 (2xC), 113.8, 112.2, 68.1; HRMS (ESI+): calcd for $C_{14}H_{13}BrNO_2^+$ $[M+H]^+$ 306.0124, found 306.0124.

2-(2-Iodophenoxy)-*N*-phenylacetamide (**4e**)

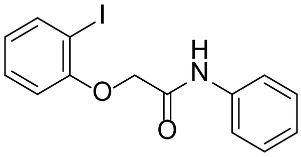

The compound **4e** was prepared according to the general procedure (**GP1**) starting from nitrile **1o** and diphenyliodonium triflate **2a.** Purification by flash chromatography using PE/EA (9:1, v/v) afforded **4e** as a white solid in 39% yield, 69 mg; m.p. 126-127 °C; ^{1}H (400 MHz, $CDCl_3$): δ 8.85 (brs, 1H), 7.83 (dd, *J* = 8.1, 1.5 Hz, 1H), 7.68 (d, *J* = 7.6 Hz, 2H), 7.40-7.35 (m, 3H), 7.17 (t, *J* = 7.4 Hz, 1H), 6.86-6.83 (m, 2H), 4.65 (s, 2H); ^{13}C (101 MHz, $CDCl_3$): δ 165.1, 155.4, 139.4, 137.1, 130.1, 129.2 (2xC), 124.8, 124.1, 119.8 (2xC), 112.5, 86.5, 68.0; HRMS (ESI+): calcd for $C_{14}H_{13}INO_2^+$ $[M+H]^+$ 353.9986, found 353.9982. NMR data correspond to the reported values.[22]

2-(3-Bromophenoxy)-*N*-phenylacetamide (**4f**)

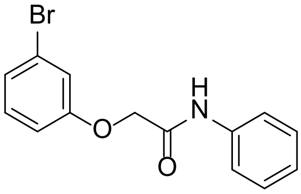

The compound **4f** was prepared according to the general procedure (**GP1**) starting from nitrile **1p** and diphenyliodonium triflate **2a.** Purification by flash chromatography using PE/EA (9:1, v/v) afforded **4f** as a white solid in 43% yield, 65 mg; m.p. 92-93 °C; ^{1}H (400 MHz, $CDCl_3$): δ 8.19 (brs, 1H), 7.59 (d, *J* = 7.7 Hz, 2H), 7.37 (t, *J* = 7.9 Hz, 2H), 7.26-7.15 (m, 4H), 6.96-6.92 (m, 1H), 4.60 (s, 2H); ^{13}C (101 MHz, $CDCl_3$): δ 165.3, 157.6, 136.7, 131.0, 129.1 (2xC), 125.7, 125.0, 123.2, 120.1 (2xC), 118.6, 113.4, 67.6; HRMS (ESI+): calcd for $C_{14}H_{13}BrNO_2^+$ $[M+H]^+$ 306.0124, found 306.0125.

22. Zhou, X.; Fu, W.; Jiang, H.; Wang, C.; Ju, C.; Chu, W.; Su, Z. *Org. Biomol. Chem.*, **2017**, 15, 1956-1960.

2-(4-Bromophenoxy)-*N*-phenylacetamide (**4g**)

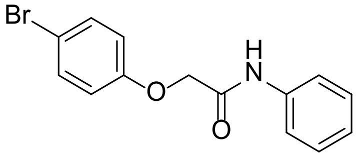

The compound **4g** was prepared according to the general procedure (**GP1**) starting from nitrile **1q** and diphenyliodonium triflate **2a.** Purification by flash chromatography using PE/EA (9:1, v/v) afforded **4g** as a white solid in 38% yield, 58 mg; m.p. 134-135 °C; ^{1}H (400 MHz, $CDCl_3$): δ 8.20 (brs, 1H), 7.58 (d, *J* = 7.6 Hz, 2H), 7.46-7.44 (m, 2H), 7.36 (t, *J* = 7.9 Hz, 2H), 7.16 (t, *J* = 7.4 Hz, 1H), 6.89-6.87 (m, 2H), 4.58 (s, 2H); ^{13}C (101 MHz, $CDCl_3$): δ 165.7, 156.1, 136.7, 132.8 (2xC), 129.1 (2xC), 125.0, 120.2 (2xC), 116.6 (2xC), 114.9, 67.8; HRMS (ESI+): calcd for $C_{14}H_{13}BrNO_2^+$ [M+H]$^+$ 306.0124, found 306.0124.

2-(4-Nitrophenoxy)-*N*-phenylacetamide (**4h**)

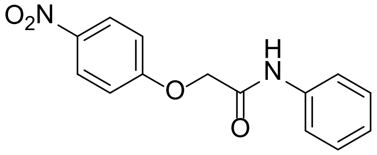

The compound **4h** was prepared according to the general procedure (**GP1**) starting from nitrile **1r** and diphenyliodonium triflate **2a.** Purification by flash chromatography using PE/EA (9:1, v/v) afforded **4h** as a yellow solid in 57% yield, 78 mg; m.p. 170-171 °C; ^{1}H (400 MHz, $CDCl_3$): δ 8.30 (d, *J* = 9.2 Hz, 2H), 8.15 (brs, 1H), 7.60 (d, *J* = 7.6 Hz, 2H), 7.40 (t, *J* = 8 Hz, 2H), 7.21 (t, *J* = 7.4 Hz, 1H), 7.12 (d, *J* = 9.2 Hz, 2H), 4.73 (s, 2H); ^{13}C (101 MHz, $CDCl_3$): δ 164.6, 161.5, 142.8, 136.4, 129.2 (2xC), 126.2 (2xC), 125.3, 120.3 (2xC), 114.9 (2xC), 67.8; HRMS (ESI+): calcd for $C_{14}H_{13}N_2O_4^+$ [M+H]$^+$ 273.0870, found 273.0870. NMR data correspond to the reported values.[23]

2-(3-Nitrophenoxy)-*N*-phenylacetamide (**4i**)

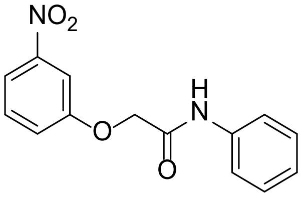

The compound **4i** was prepared according to the general procedure (**GP1**) starting from nitrile **1s** and diphenyliodonium triflate **2a.** Purification by flash chromatography using PE/EA (9:1, v/v) afforded **4i** as a white solid in 44% yield, 60 mg; m.p. 124-125 °C; ^{1}H (400 MHz, $CDCl_3$): δ 8.24 (brs, 1H), 7.97-7.88 (m, 2H), 7.63-7.53 (m, 3H), 7.41-7.35 (m, 3H), 7.20 (t, *J* = 7.3 Hz, 1H), 4.71 (s, 2H); ^{13}C (101 MHz, $CDCl_3$): δ 164.9, 157.4, 149.3, 136.6, 130.6, 129.2 (2xC), 125.2, 121.0, 120.3 (2xC), 117.4, 110.1, 67.9; HRMS (ESI+): calcd for $C_{14}H_{13}N_2O_4^+$ [M+H]$^+$ 273.0870, found 273.0868.

2-(2-Nitrophenoxy)-*N*-phenylacetamide (**4j**)

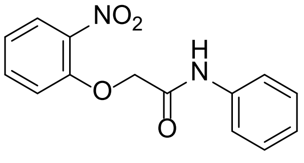

23. Baraldi, P. G.; Preti, D.; Tabrizi, M. A.; Fruttarolo, F.; Saponaro, G.; Baraldi, S.; Romagnoli, R.; Moorman,A. R.; Gessi, S.; Varaniband, K.; Borea, P. A. *Bioorg. Med. Chem.*, **2007**, *15*, 2514-2527.

The compound **4j** was prepared according to the general procedure (**GP1**) starting from nitrile **1t** and diphenyliodonium triflate **2a.** Purification by flash chromatography using PE/EA (9:1, v/v) afforded **4j** as a brown solid in 20% yield, 27 mg; m.p. 118-119 °C; ^{1}H (400 MHz, $CDCl_3$): δ 9.19 (brs, 1H), 8.13 (dd, *J* = 8.2, 1.6 Hz, 1H), 7.75 (d, *J* = 7.6 Hz, 2H), 7.71-7.67 (m, 1H), 7.40 (t, *J* = 8 Hz, 2H), 7.23-7.13 (m, 3H), 4.79 (s, 2H); ^{13}C (101 MHz, $CDCl_3$): δ 164.5, 150.9, 137.2, 135.6, 129.1 (2xC), 126.9, 124.8, 122.1, 119.7 (2xC), 114.8, 100.0, 67.9; HRMS (ESI+): calcd for $C_{14}H_{13}N_2O_4^+$ [M+H]$^+$ 273.0870, found 273.0870.

2-(4-Methoxyphenoxy)-*N*-phenylacetamide (**4k**)

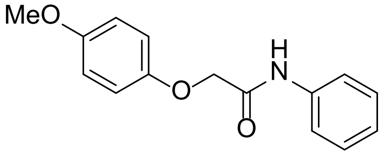

The compound **4k** was prepared according to the general procedure (**GP1**) starting from nitrile **1u** and diphenyliodonium triflate **2a.** Purification by flash chromatography using PE/EA (9:1, v/v) afforded **4k** as a white solid in 43% yield, 55 mg; m.p. 80-81 °C; ^{1}H (400 MHz, $CDCl_3$): δ 8.29 (brs, 1H), 7.59 (d, *J* = 7.6 Hz, 2H), 7.36 (t, *J* = 7.9 Hz, 2H), 7.16 (t, *J* = 7.4 Hz, 1H), 6.95-6.87 (m, 4H), 4.56 (s, 2H), 3.79 (s, 3H); ^{13}C (101 MHz, $CDCl_3$): δ 166.5, 155.0, 151.1, 136.9, 129.1 (2xC), 124.9, 120.1 (2xC), 115.9 (2xC), 114.9 (2xC), 68.5, 55.7; HRMS (ESI+): calcd for $C_{15}H_{16}NO_3^+$ [M+H]$^+$ 258.1125, found 258.1124.

2-(4-(*Tert*-butyl)phenoxy)-*N*-phenylacetamide (**4l**)

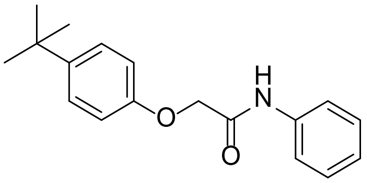

The compound **4l** was prepared according to the general procedure (**GP1**) starting from nitrile **1v** and diphenyliodonium triflate **2a.** Purification by flash chromatography using PE/EA (9:1, v/v) afforded **4l** as a white solid in 67% yield, 92 mg; m.p. 95-96 °C; ^{1}H (400 MHz, $CDCl_3$): δ 8.30 (brs, 1H), 7.63-7.61 (m, 2H), 7.40-7.36 (m, 4H), 7.18 (t, *J* = 7.4 Hz, 1H), 6.98-6.94 (m, 2H), 4.63 (s, 2H), 1.34 (s, 9H); ^{13}C (101 MHz, $CDCl_3$): δ 166.5, 154.8, 145.4, 136.9, 129.1 (2xC), 126.7 (2xC), 124.8, 120.1 (2xC), 114.4 (2xC), 67.8, 34.2, 31.5 (3 x C); HRMS (ESI+): calcd for $C_{18}H_{22}NO_2^+$ [M+H]$^+$ 258.1645, found 258.1645.

2-(2-Bromo-4-(*tert*-butyl)phenoxy)-*N*-phenylacetamide (**4m**)

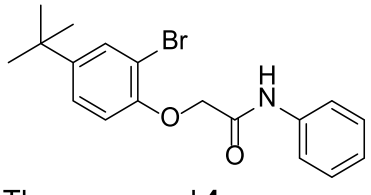

The compound **4m** was prepared according to the general procedure (**GP1**) starting from nitrile **1w** and diphenyliodonium triflate **2a.** Purification by flash chromatography using PE/EA (9:1, v/v) afforded **4m** as a white solid in 34% yield, 62 mg; m.p. 91-92 °C; ^{1}H (400 MHz, $CDCl_3$): δ 8.78 (brs, 1H), 7.68-7.62 (m, 3H), 7.41-7.34 (m, 3H), 7.18 (t, *J* = 7.4 Hz, 1H), 6.89 (d, *J* = 8.6 Hz, 1H), 4.66 (s, 2H), 1.33 (s, 9H); ^{13}C (101 MHz, $CDCl_3$): δ 165.6, 151.2, 147.1, 137.1, 130.6, 129.1 (2xC), 125.8, 124.8, 119.9 (2xC), 113.5, 111.9, 68.4, 34.4, 31.3 (3 x C); HRMS (ESI+): calcd for $C_{18}H_{21}BrNO_2^+$ [M+H]$^+$ 362.0750, found 362.0748.

2-(2-Ethylphenoxy)-*N*-phenylacetamide (**4n**)

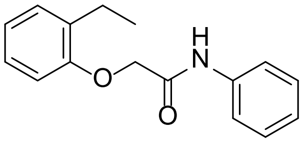

The compound **4n** was prepared according to the general procedure (**GP1**) starting from nitrile **1x** and diphenyliodonium triflate **2a.** Purification by flash chromatography using PE/EA (9:1, v/v) afforded **4n** as a white solid in 68% yield, 87 mg; m.p. 66-67 °C; ^{1}H (400 MHz, $CDCl_3$): δ 8.37 (brs, 1H), 7.61 (d, *J* = 7.6 Hz, 2H), 7.40 (t, *J* = 7.9 Hz, 2H), 7.28-7.17 (m, 3H), 7.04 (t, *J* = 7.8 Hz, 1H), 6.89 (d, *J* = 8.1 Hz, 1H), 4.65 (s, 2H), 2.81 (q, *J* = 7.5 Hz, 2H), 1.35 (t, *J* = 7.6 Hz, 3H); ^{13}C (101 MHz, $CDCl_3$): δ 166.4, 154.8, 136.9, 132.5, 129.6, 129.2 (2xC), 127.4, 124.9, 122.4, 119.9 (2xC), 112.0, 67.8, 23.6, 14.3; HRMS (ESI+): calcd for $C_{16}H_{18}NO_2^+$ $[M+H]^+$ 256.1332, found 256.1331.

2-(2-Naphthyloxy)-*N*-phenylacetamide (**4o**)

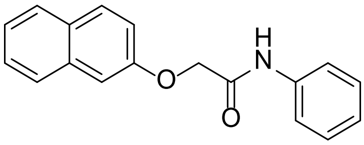

The compound **4o** was prepared according to the general procedure (**GP1**) starting from nitrile **1y** and diphenyliodonium triflate **2a.** Purification by flash chromatography using PE/EA (9:1, v/v) afforded **4o** as a white solid in 40% yield, 56 mg; m.p. 143-144 °C; ^{1}H (400 MHz, $CDCl_3$): δ 8.35 (brs, 1H), 7.87-7.78 (m, 3H), 7.64 (d, *J* = 8.6 Hz, 2H), 7.53-7.49 (m, 1H), 7.45-7.37 (m, 3H), 7.31-7.25 (m, 2H), 7.19 (t, *J* = 7.4 Hz, 1H), 4.77 (s, 2H); ^{13}C (101 MHz, $CDCl_3$): δ 166.1, 154.8, 136.8, 134.3, 130.1, 129.6, 129.1 (2xC), 127.7, 127.1, 126.9, 124.9, 124.6, 120.2 (2xC), 118.0, 107.9, 67.6; HRMS (ESI+): calcd for $C_{18}H_{16}NO_2^+$ $[M+H]^+$ 278.1176, found 278.1175. NMR data correspond to the reported values.

2-([1,1'-biphenyl]-2-yloxy)-*N*-phenylacetamide (**4p**)

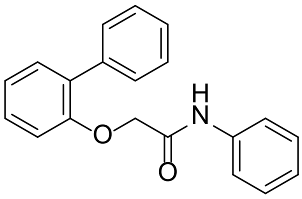

The compound **4o** was prepared according to the general procedure (**GP1**) starting from nitrile **1z** and diphenyliodonium triflate **2a.** Purification by flash chromatography using PE/EA (9:1, v/v) afforded **4o** as a white solid in 20% yield, 31 mg; m.p. 109-110 °C; ^{1}H (400 MHz, $CDCl_3$): δ 8.07 (brs, 1H), 7.58-7.45 (m, 5H), 7.40-7.36 (m, 2H), 7.29-7.25 (m, 4H), 7.16-7.07 (m, 2H), 7.00-6.97 (m, 1H), 4.60 (s, 2H); ^{13}C (101 MHz, $CDCl_3$): δ 165.8, 153.8, 138.4, 137.0, 131.2, 131.0, 129.5 (2xC), 129.1, 128.9 (2xC), 128.6 (2xC), 127.5, 124.5, 122.5, 119.5 (2xC), 112.7, 67.7; HRMS (ESI+): calcd for $C_{20}H_{18}NO_2^+$ $[M+H]^+$ 304.1332, found 304.1332.

III. Synthesis of 4-phenyl-2H-benzo[b][1,4]oxazin-3(4H)-one (5)

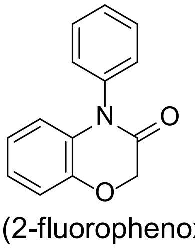

(2-fluorophenoxy)-N-phenylacetamide **4b** (80mg, 0.3 mmol, 1 equiv.) and Cs_2CO_3 (160 mg, 0.45 mmol, 1.5 equiv.) were placed in an oven dried sealable tube with 3.5ml of toluene. Mixture was stirred at 120°C for 1.5h, progress was monitored by NMR. Then it was diluted

with 5 ml of 1N HCl solution and extracted 3 x 10ml of DCM. Combined organic phase was dried over anhydrous MgSO4. Solvent was removed on rotary evaporator. Colum chromatography was performed (SiO_2, eluent: petroleum ether/AcOEt, 5:1) to afford **5** as a pure compound. NMR data correspond to the reported values.[24]

White solid in 63% yield, 46 mg; m.p. 91-92 °C; ^{1}H (400 MHz, $CDCl_3$): δ 7.55 (m, 2H) 7.48 (m, 1H) 7.30 (m, 2H) 7.02 (m, 2H) 6.87 (m, 1H) 6.45 (m, 1H) 4.79 (s, 2H); ^{13}C (101 MHz, $CDCl_3$): δ 164.4, 145.0, 135.9, 130.7, 130.1, 129.0, 128.9, 124.2, 122.7, 117.1, 117.0, 68.3; HRMS (ESI+): calcd for $C_{14}H_{12}NO_2^+$ [M+H]$^+$ 226.0863, found 226.0859.

IV. CV Experiments

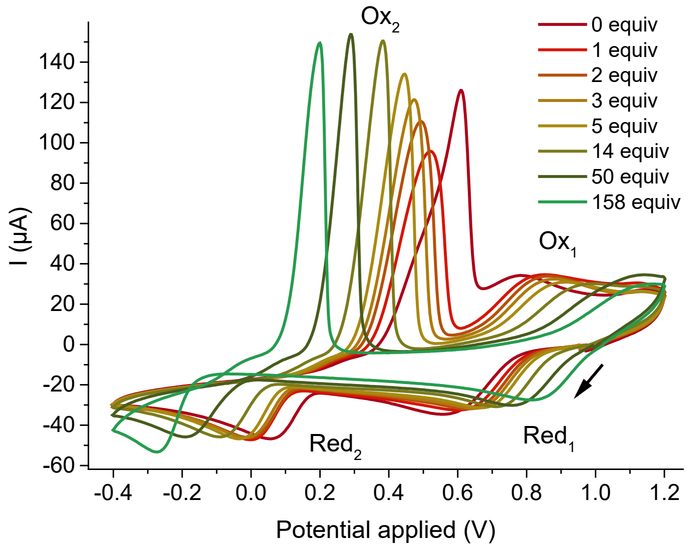

Figure S1. CV towards reduction potentials of $Cu^{II}(OTf)_2$ (2 mM) with increasing amounts of MeCN (from red to green) recorded at a steady glassy carbon disk electrode (d = 3 mm) in nitromethane containing *n*-Bu_4NBF_4 (0.3 M) at 20 °C with a scan rate of 0.5 V s^{-1}.

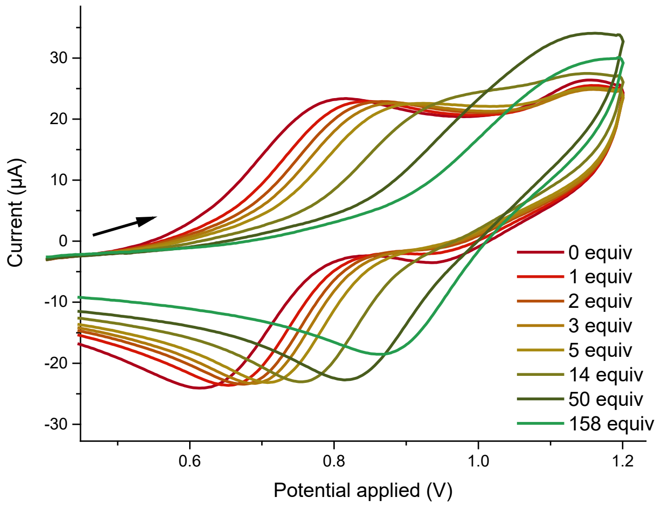

Figure S2. CV towards oxidation potentials of electrogenerated $Cu^I(OTf)$ (2 mM) with increasing amounts of MeCN (from red to green) recorded at a steady glassy carbon disk electrode (d = 3 mm) in nitromethane containing *n*-Bu_4NBF_4 (0.3 M) at 20 °C with a scan rate of 0.5 V s^{-1}.

---

24. Feng, G.; Wang, S.; Li, W.; Chen, F.; Qi, C. *Synthesis,* **2013**, *45*, 2711-2718.

More in detail, it was possible to obtain quantitative data about the stoichiometry of the $[Cu(I)(MeCN)_n]^+$ complexes by plotting the half peak potential as a function of MeCN concentration (Figure S3). The variation of the half-wave potential ($E_{1/2}$) associated to Cu(I)/Cu(0) *vs* pMeCN = -log([MeCN]) (slope comprised between 0.10 and 0.18 mV) is consistent with the formation of several complexes featuring between 2 and 3 MeCN per Cu(I).

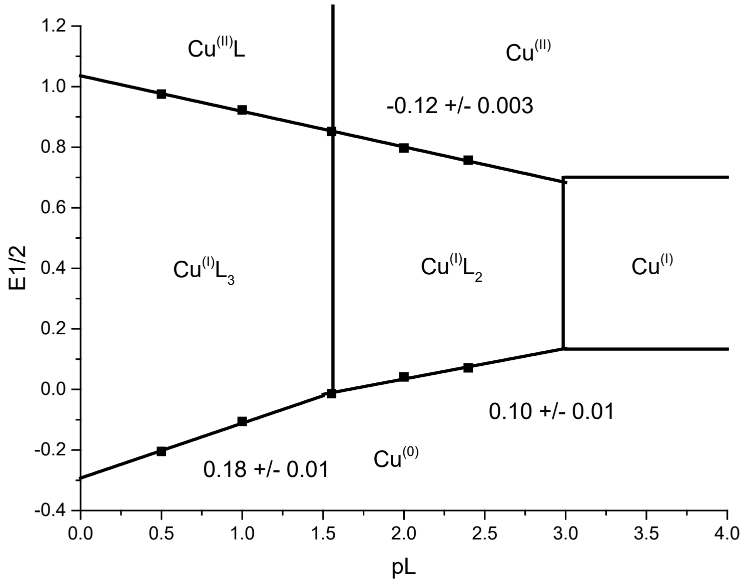

Figure S3. Potential-pL (L = MeCN) plot constructed using the $E_{1/2}$=E(Ipeak/2) values extracted from the CV plots.

The following equilibria are considered,

$$\mathrm{Cu^0 + n\,MeCN = [Cu^I(MeCN)_n] + e^-}$$

The associated Nernst equation can be written as (C° = 1 mol L^{-1} is the standard concentration):

$$E = E°\left(\mathrm{Cu^0, [Cu^I(MeCN)}_n]\right) + \frac{RT}{F}\ln\left(\frac{[\mathrm{Cu^I(MeCN)}_n]}{(C°)^{1-n}\,[\mathrm{MeCN}]^n}\right)$$

$$E = E°\left(\mathrm{Cu^0, [Cu^I(MeCN)]}\right) - \frac{\alpha RT}{F}\ln\left(\frac{[\mathrm{MeCN}]}{C°}\right) + \frac{RT}{F}\ln\left(\frac{[\mathrm{Cu^I(MeCN)}_\alpha]}{C°}\right)$$

$$E = E°\left(\mathrm{Cu^0, [Cu^I(MeCN)]}\right) + \frac{nRT}{F}\ln(10)\,\mathrm{pMeCN} + \frac{RT}{F}\ln\left(\frac{[\mathrm{Cu^I(MeCN)}_\alpha]}{C°}\right)$$

At 298 K, $\frac{RT}{F}\ln(10) = 0.059$ V and consequently n = 2 (slope 0.12 V) or n = 3 (slope n = 0.18). Conversely Cu(II) displays only a weak affinity toward acetonitrile. The negative slope (-0.12 V *per* decade) obtained for the oxidation peak of Cu(I) to Cu(II) is consistent with no interaction between Cu(II) and acetonitrile (see the supporting information Figures S3). The slope of frontier associated with the Cu(I) oxidation have a negative slope -0.12 V. This is consistent with no interaction between Cu(I) and MeCN. The following equilibrium is then considered,

$$[\mathrm{Cu^I(MeCN)}_n] = [\mathrm{Cu^{II}(MeCN)_m}] + \mathrm{e^-} + (\mathrm{n-m})\,\mathrm{MeCN}$$

The Nernst equation can be written as:

$$E = E°\left([\mathrm{Cu^{II}(MeCN)}_m], [\mathrm{Cu^I(MeCN)}_n]\right) + \frac{RT}{F}\ln\left(\frac{[\mathrm{Cu^{II}(MeCN)}_m](C°)^{n-m}}{[\mathrm{Cu^I(MeCN)}_n][\mathrm{MeCN}]^{(n-m)}}\right)$$

$$E = E°\left(\mathrm{Cu^0, [Cu^I(MeCN)}_n]\right) + \frac{(m-n)RT}{F}\ln(10)\,\mathrm{pL} + \frac{RT}{F}\ln\left(\frac{[\mathrm{Cu^{II}(MeCN)}_{1+\beta}]}{[\mathrm{Cu^I(MeCN)}]}\right)$$

Assuming that n = -2 (*vide supra*), the only possible solution is m = 0.

Cyclohexylformamide (CyNHCHO) was chosen as a model secondary amide to study the possible complexation of copper by the product. Upon addition of cyclohexylformamide on a solution of $Cu^{II}(OTf)_2$ in the presence of a large excess of MeCN, the reduction peak of Cu^{II} was shifted to lower potentials due to the formation of tetracoordinated $[Cu^{II}(CyNHCHO)_4]^{2+}$, while the reduction peak of Cu^{I} remained almost unchanged consistently with a lack of interaction of the product with Cu^{I} without a base. The amide is thus not responsible for the observed inhibition of the copper catalyst but the ketenimine most probably is as confirmed by the DFT calculations.

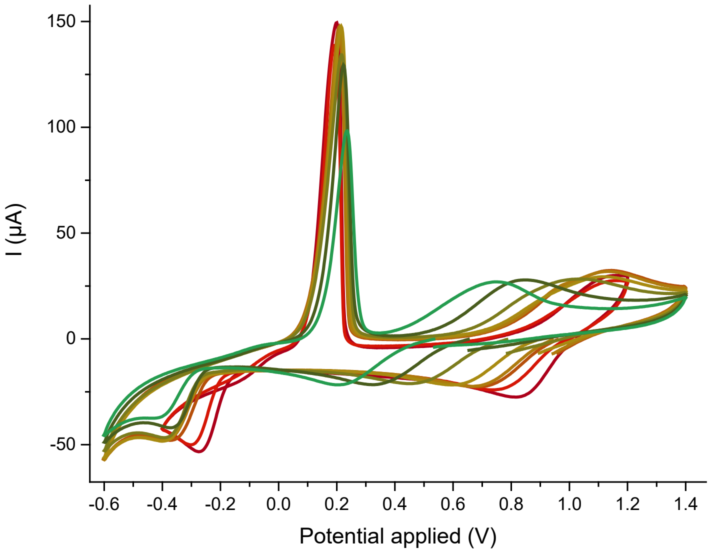

Figure S4. CV towards reduction (top) and oxidation (bottom) potentials of $Cu^{II}(OTf)_2$ (1 mM) in the presence of MeCN (158 equiv) with increasing amounts of cyclohexylformamide (from red to green), recorded at a steady glassy carbon disk electrode (d = 3 mm) in nitromethane containing *n*-Bu_4NBF_4 (0.3 M) at 20 °C with a scan rate of 0.5 V s^{-1}. (0, 1, 2, 5, 14, 50, 158 equiv).

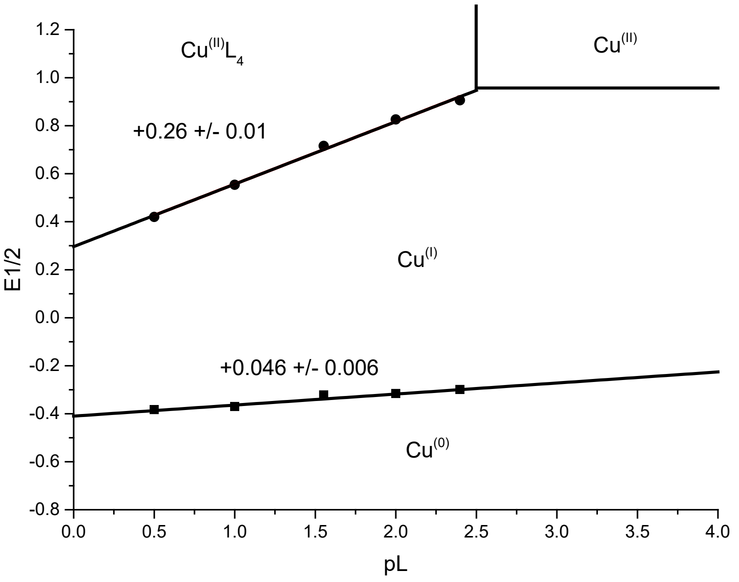

Figure S5. Potential-pL (L = cyclohexylformamide) plot in the presence of excess MeCN (pMeCN = 0.5) using the E(Ipeak/2) values extracted from the CV plots.

V. DFT calculations

DFT calculations of formation energies of nitrile-ligated copper(I) complexes

The formation of $[Cu(I)(MeCN)_3]^+$ **c3** is predicted as the most exergonic process (Table S1) but formation of $[Cu(I)(MeCN)_2]^+$ **c2** is only 4.8 kcal mol^{-1} higher in energy. If the formation of monoligated $[Cu(I)(MeCN)]^+$ **c1** seems to be highly unlikely (+35.8 kcal mol^{-1}), the tetracoordinated $[Cu(I)(MeCN)_4]^+$ **c4** may be accessible in concentrated solution (+ 1.1 kcal mol^{-1}).

Table S1 DFT calculated free energy and enthalpy of complexation of MeCN to Cu(I).

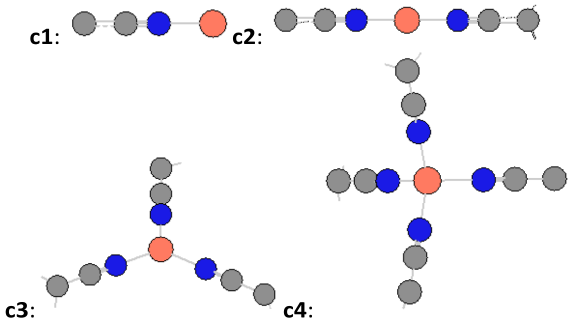

Complex	Δ*H*° (kcal mol^{-1})	Δ*G*° (kcal mol^{-1})	ΔΔ*G*° (kcal mol^{-1})
$[Cu(I)(MeCN)_1]^+$ (**c1**)	-40.5	-33.5	+35.8
$[Cu(I)(MeCN)_2]^+$ (**c2**)	-82.4	-64.5	+4.8
$[Cu(I)(MeCN)_3]^+$ (**c3**)	-94.5	-69.3	0
$[Cu(I)(MeCN)_4]^+$ (**c4**)	-102.6	-68.2	1.1

DFT calculations of hydroxo-ligated copper(I) complexes

To identify the possible species formed in the presence of hydroxides and the two other ligands - acetonitrile and secondary amide - DFT calculations were performed. Both monomeric and dimeric copper(I) complexes were considered. A monomeric copper complex [Cu(OH)(MeCN)] **c5** could be found featuring one acetonitrile complexed by the nitrogen atom, and one hydroxide (Figure S6). Addition of more acetonitrile or more hydroxide ligands to this species resulted in their decomplexation. A more favourable dimeric complex $[Cu(OH)(MeCN)]_2$ **c6** (Figure S6) could be formed with two bridging hydroxyl groups, and two copper centres, each bearing one acetonitrile ligand.

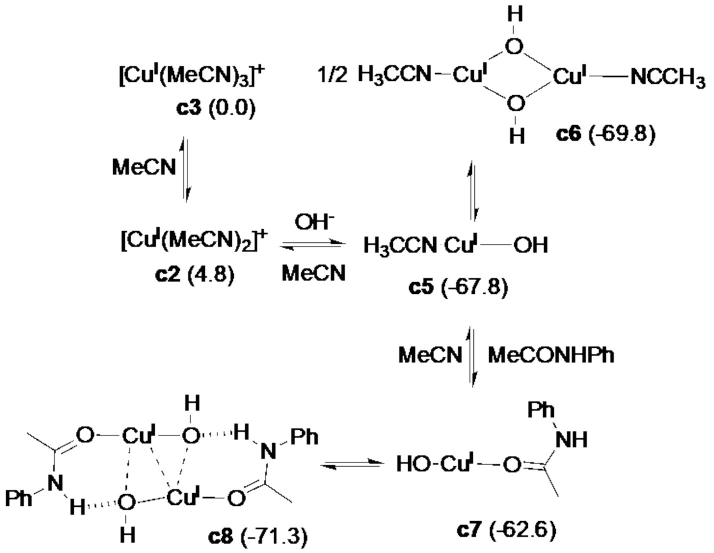

Figure S6. Different possible copper-ligated species, DFT estimated free energy in kcal mol^{-1} are indicated between brackets.

The formation of mixed species between the product *N*-phenylacetamide (PhNHCOMe) and hydroxides was considered (Figure S6). A stable monomeric copper complex [Cu(OH)(PhNHCOMe)] **c7** could be found featuring one *O*-ligated *N*-phenylacetamide and one hydroxide. A more favorable dimeric complex $[Cu(OH)(PhNHCOMe)]_2$ **c8** could be formed with two hydroxides bridging the PhNHCOMe and a Cu site. to the present of two hydrogen bonds in this dimeric species, results in a very stable copper complex bearing the product of the reaction, and thus potentially poisoning the catalyst. Indeed, ligand exchange to form the initial Cu(I) complexes **c5-6** bearing acetonitrile is not favorable and the catalyst cannot easily be regenerated in the reaction medium.

Finally, it is also possible to consider the formation of anionic complexes (Figure S7) featuring either two hydroxides ($[Cu(OH)_2]^-$ complex **c12**) or one hydroxide and one deprotonated acetonitrile ($[Cu(OH)(CH_2CN)]^-$ complex **c13**). Such species should not however be relevant for catalysis are they are likely to be scarcely soluble in non-polar solvents such as toluene.

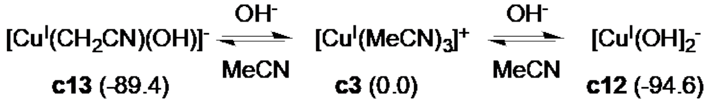

Figure S7. Possibly formed anionic copper-ligated species, DFT estimated free energy in kcal mol^{-1} are indicated between brackets.

For the sake of completeness, below we report an alternative pathway of reaction that can be envisaged starting from anionic complexes **c12**.

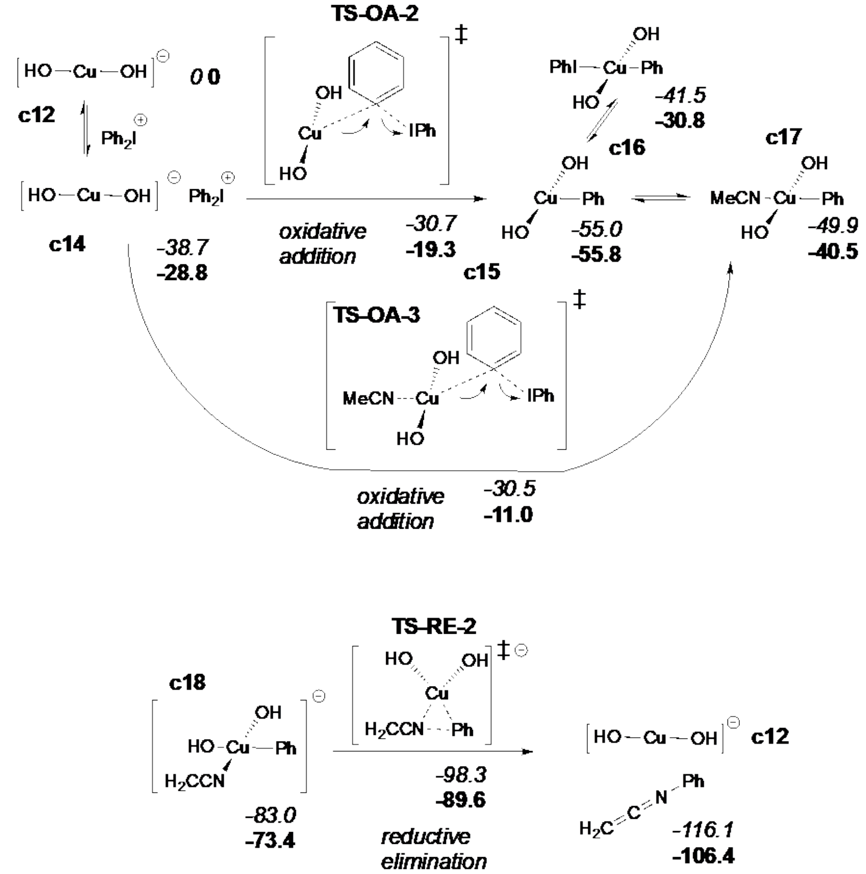

Figure S8. Alternative pathway I for OA and RE leading to the ketenimide product, enthalpy and free energy (bold characters) in kcal mol^{-1}.

VI. Kinetics

Mass spectrometry analysis were performed by the "Fédération de Recherche" ICOA/CBM (FR2708) platform of Orléans in France. High-resolution ESI mass spectra (HRMS) were recorded on a maXis Q-TOF (Bruker, Bremen, Germany) in the positive ion mode. The samples were diluted 2500 times in CH_3CN. The diluted solutions (0.3 µL) were delivered to the ESI source by a Dionex Ultimate 3000 RSLC chain used in FIA (Flow Injection Analysis) mode at a flow rate of 200 µL/min with a mixture of CH3CN/H2O+0.1% of HCO2H (65/35). ESI conditions were as follows: capillary voltage was set at 4.5 kV; dry nitrogen was used as nebulizing gas at 0.6 bar and as drying gas set at 200°C and 7.0 L/min. ESI-MS spectra were recorded at 1 Hz in the range of 50-2500 m/z. Calibration was performed with ESI-TOF Tuning mix from Agilent and corrected using lock masses at m/z 299.294457 (methyl stearate) and 1221.990638 (HP-1221). Data were processed using Bruker DataAnalysis 4.4 software. Measured area were normalized to 100% for the maximum area in order to plot evolution curves. Preparation of **3aa** was conducted according to the GP1. A dilution factor of 2500 in CH_3CN was adopted before the injection.

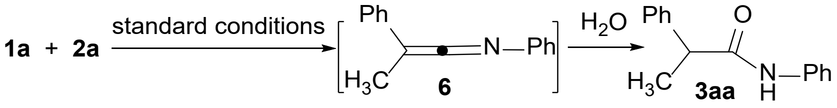

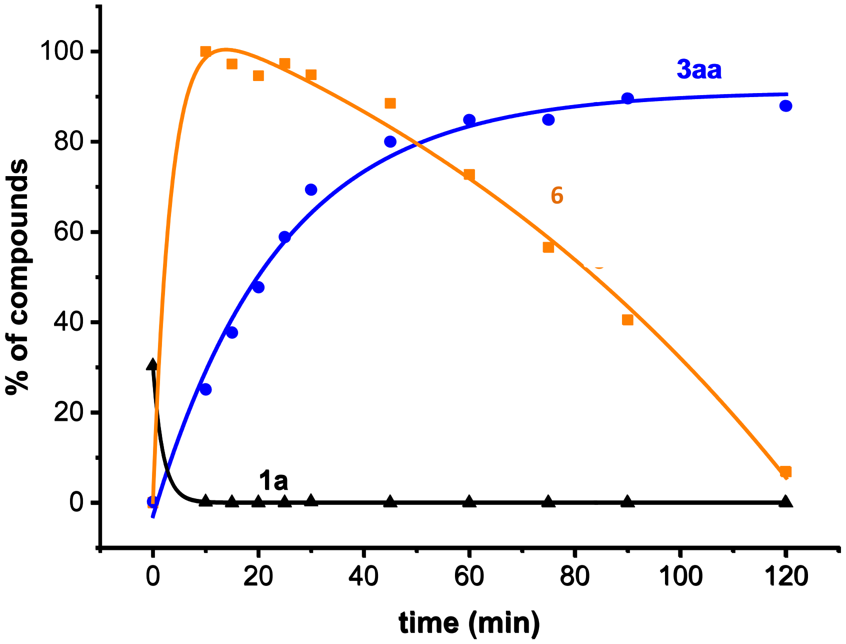

Figure S9. Time course experiments of Cu-catalyzed *N*-arylation of **1a** (LC-HRMS). HPLC yield accounting for the response factor of **1a** (black curve), **3aa** (blue curve) and **6** (orange curve).

When adding a stoichiometric amount of final acetamide **3aa** at the beginning of the reaction, no evolution could be detected consistently with an inhibition of the catalyst by the product (see Figure 10).

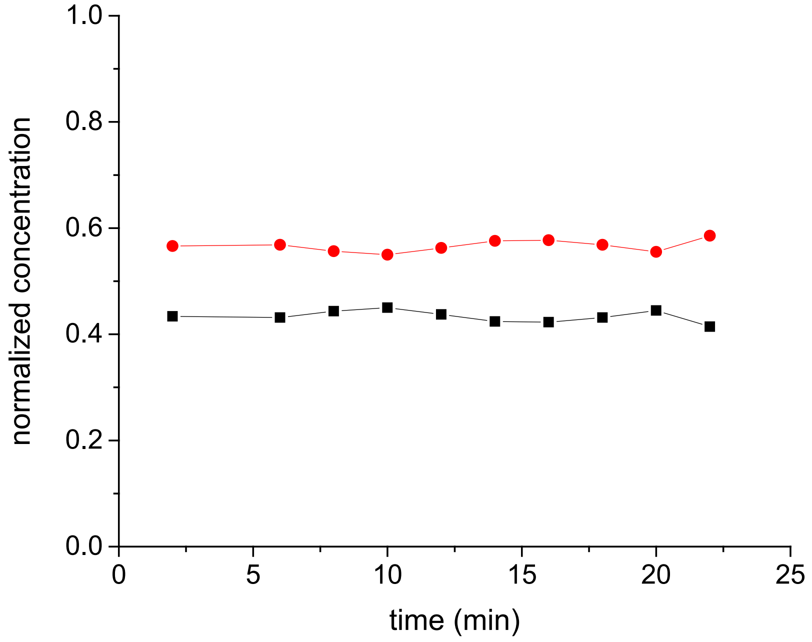

Figure S10. Time course experiments of Cu-catalyzed N-arylation of **1a** (LC-HRMS). HPLC yield accounting for the response factor of **6** (black curve), **3aa** (red curve).

VII. Computational details and Cartesian coordinates

All calculations were performed using the Gaussian 09 program (Revision A.02).[25] The structures were optimized using 6-311+G(d,p) basis set on the main group elements (H, C, N, O) while Copper and iodine were described using the Los-Alamos double zeta basis set and associated pseudo-potential.[26] The B3LYP functional was used.[27] Bulk solvent effects were introduced using a Polarizable Continuum Model to model toluene.[28] All stationary points were fully characterized via a subsequent analytical frequency calculation as minima or first order transition states. Enthalpies and free energies were calculated for standard conditions at 298.15 K and 1 Atm.

MeCN

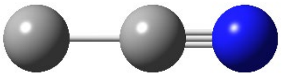

E(B3LYP)= -132.800378261 Hartree

N	-0.91420500	-1.16102300	0.00150100
C	-2.06732000	-1.15915500	0.00079200
C	-3.52292100	-1.15662500	-0.00009900
H	-3.89919600	-2.10749100	0.38163700
H	-3.89630700	-0.34946200	0.63276300
H	-3.89645900	-1.01100600	-1.01529800

PhNHCOMe

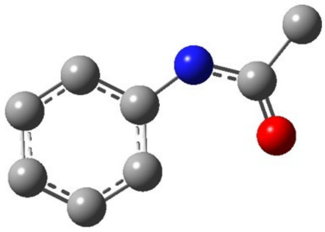

E(B3LYP) = -440.396193132 Hartree

O	1.32007700	-0.15741100	0.20374900
C	0.90929300	0.99025500	0.11594500
C	1.86077700	2.17290100	0.09428600
H	2.46515300	2.14880100	1.00334100
H	2.53840900	2.05726100	-0.75421800
H	1.36467800	3.14227200	0.02263900
N	-0.42156000	1.32239800	0.02860900
H	-0.62418300	2.30851000	-0.03804500
C	-1.55916800	0.48462000	0.01713800
C	-2.81041100	1.11051900	-0.09012900
C	-1.49487000	-0.91213300	0.10642800

[25] M. J. Frisch, G. W. Trucks, H. B. Schlegel, G. E. Scuseria, M. A. Robb, J. R. Cheeseman, G. Scalmani, V. Barone, B. Mennucci, G. A. Petersson, H. Nakatsuji, M. Caricato, X. Li, H. P. Hratchian, A. F. Izmaylov, J. Bloino, G. Zheng, J. L. Sonnenberg, M. Hada, M. Ehara, K. Toyota, R. Fukuda, J. Hasegawa, M. Ishida, T. Nakajima, Y. Honda, O. Kitao, H. Nakai, T. Vreven, J. A. Montgomery, Jr., J. E. Peralta, F. Ogliaro, M. Bearpark, J. J. Heyd, E. Brothers, K. N. Kudin, V. N. Staroverov, R. Kobayashi, J. Normand, K. Raghavachari, A. Rendell, J. C. Burant, S. S. Iyengar, J. Tomasi, M. Cossi, N. Rega, N. J. Millam, M. Klene, J. E. Knox, J. B. Cross, V. Bakken, C. Adamo, J. Jaramillo, R. Gomperts, R. E. Stratmann, O. Yazyev, A. J. Austin, R. Cammi, C. Pomelli, J. W. Ochterski, R. L. Martin, K. Morokuma, V. G. Zakrzewski, G. A. Voth, P. Salvador, J. J. Dannenberg, S. Dapprich, A. D. Daniels, Ö. Farkas, J. B. Foresman, J. V. Ortiz, J. Cioslowski, D. J. Fox, Gaussian, Inc., Wallingford CT., **2009**.

[26] (a) Hay, P. J.; Wadt, W. R. *J. Chem. Phys.* **1985,** *82*, 270-283; (b) Wadt, W. R.; Hay, P. J. *J. Chem. Phys.* **1985,** *82*, 284-298; (c) Hay, P. J.; Wadt, W. R. *J. Chem. Phys.* **1985,** *82*, 299-310.

[27] (a) Becke, A. D. *J. Chem. Phys.* **1993,** 98, 1372-1377; (b) Lee, C.; Yang, W.; Parr, R. G. *Phys. Rev. B* **1988,** 37, 785-789; (c) Vosko, S. H.; Wilk, L.; Nusair, M. *Can. J. Phys.* **1980,** 58, 1200-1211; (d) Stephens, P. J.; Devlin, F. J.; Chabalowski, C. F.; Frisch, M. J. *J. Phys. Chem*. **1994,** 98, 11623-11627.

[28] (a) Tomasi, J.; Mennucci, B.; Cammi, R. *Chem. Rev.* **2005,** *105*, 2999-3094; (b) Cossi, M.; Scalmani, G.; Rega, N.; Barone, V. *J. Chem. Phys.* **2002,** *117*, 43-54.

C	-3.97789300	0.35686800	-0.10724600
H	-2.86544800	2.19255600	-0.16061700
C	-2.67547500	-1.65415900	0.08766900
H	-0.53578600	-1.39869600	0.18863200
C	-3.91827400	-1.03410100	-0.01818100
H	-4.93498300	0.85922700	-0.19049000
H	-2.61430100	-2.73453300	0.15747600
H	-4.82749400	-1.62349300	-0.03128800

OH^-

E(RB3LYP) = -75.9034987330 Hartree

O	0.06497700	0.85918000	0.00000000
H	-0.25689700	1.76812100	0.00000000

Ph_2I^+

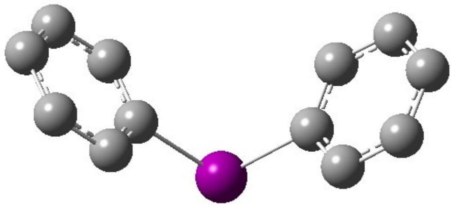

E(B3LYP) = -474.526186882 Hartree

I	0.00345300	-1.35551700	-0.15813200
C	-1.65800400	0.02146100	-0.01353900
C	-2.11447600	0.36929500	1.25007400
C	-2.21982900	0.48010300	-1.19708900
C	-3.19951600	1.24381600	1.31980500
H	-1.65912200	-0.01509200	2.15260500
C	-3.30375700	1.35305600	-1.09571600
H	-1.84266200	0.17987400	-2.16525000
C	-3.78823300	1.73242400	0.15466100
H	-3.57976400	1.53560300	2.29124900
H	-3.76400900	1.72996300	-2.00095300
H	-4.63200900	2.40825100	0.22170300
C	1.66032300	0.03079300	-0.03414900
C	2.06477800	0.67322200	-1.19599800
C	2.26985200	0.20053400	1.20128000
C	3.14341100	1.55279000	-1.09835000
H	1.57546200	0.50656400	-2.14595000
C	3.34824300	1.08401300	1.26715900
H	1.93384800	-0.32552400	2.08451600
C	3.77886800	1.75694000	0.12545500
H	3.48243700	2.07195700	-1.98654000
H	3.84566800	1.23896800	2.21684900
H	4.61706000	2.44013400	0.18813500

PhI (iodobenzene)

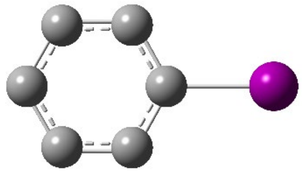

E(RB3LYP) = -243.088889690 Hartree

C	-3.34375800	0.00007100	-0.00000400
C	-2.64402100	1.20532600	-0.00000300

C	-1.24848600	1.21358300	0.00000000
C	-0.56624100	-0.00005100	0.00000400
C	-1.24859100	-1.21362100	0.00000400
C	-2.64413000	-1.20524100	0.00000000
H	-4.42746400	0.00012300	-0.00000600
H	-3.17942000	2.14806900	-0.00000600
H	-0.70943300	2.15199600	0.00000100
H	-0.70962700	-2.15208500	0.00000600
H	-3.17960600	-2.14794000	0.00000000
I	1.57244300	-0.00013800	0.00001000

Complex c1 $[Cu(MeCN)]^+$

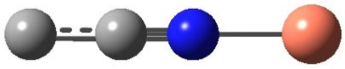

E(B3LYP) = -328.775584259 Hartree

Cu	0.96384100	-1.15753400	0.00021100
N	-0.92994500	-1.15967200	0.00099900
C	-2.07974800	-1.15921900	0.00086200
C	-3.52542600	-1.15677200	-0.00006100
H	-3.89338300	-2.11086900	0.38297600
H	-3.89031300	-0.34682900	0.63503500
H	-3.89027700	-1.01067700	-1.01874000

Complex c2 $[Cu(MeCN)_2]^+$

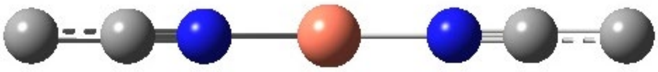

E(B3LYP) = -461.644716813 Hartree

Cu	0.95115700	-1.15681200	-0.00001400
N	2.82950500	-1.15332700	-0.00122900
N	-0.92719200	-1.16030600	0.00120500
C	3.97896300	-1.15390800	-0.00101400
C	-2.07664900	-1.15972400	0.00100500
C	-3.52314000	-1.15680900	-0.00005200
H	-3.89235800	-2.11038600	0.38281100
H	-3.88849600	-0.34696300	0.63474800
H	-3.88857200	-1.01057300	-1.01842000
C	5.42545400	-1.15681400	0.00006000
H	5.79466900	-0.20321800	-0.38275600
H	5.79087400	-1.30309200	1.01842700
H	5.79082500	-1.96663000	-0.63477100

Complex c3 $[Cu(MeCN)_3]^+$

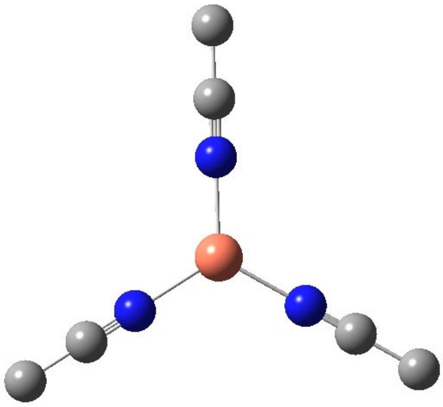

E(B3LYP) = -594.465526297 Hartree

Cu	0.94872400	-0.21205200	0.93814000
N	2.66428400	-0.92561700	0.22744900
N	-0.76680500	-0.92356000	0.22613000
C	3.65517500	-1.34203300	-0.18265700
C	-1.75817100	-1.33668200	-0.18615700
C	-3.00659700	-1.85808800	-0.70639100
H	-3.06139700	-2.93298000	-0.52373800
H	-3.84767000	-1.36682100	-0.21367000
H	-3.06299700	-1.67469600	-1.78107000
C	4.90353700	-1.86713400	-0.69949600
H	5.31548200	-1.17654100	-1.43778800
H	5.61957700	-1.98817500	0.11563200
H	4.73052200	-2.83561100	-1.17253000
N	0.95083700	1.19002100	2.34225400
C	0.95429500	2.00224100	3.15694900
C	0.95822600	3.02612300	4.18322800
H	0.54508500	3.95275600	3.78012600
H	0.35019800	2.70245600	5.03015500
H	1.97967300	3.20664400	4.52312100

Complex c4 $[Cu(MeCN)_4]^+$

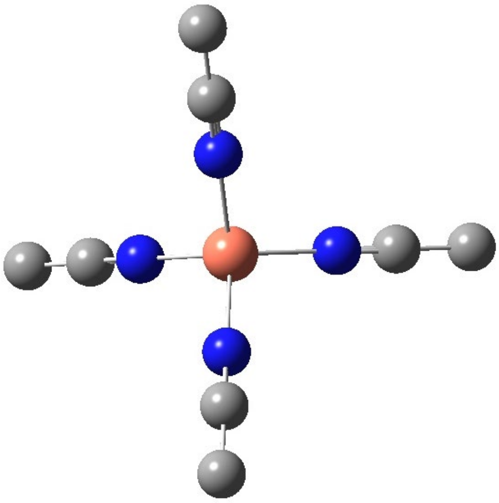

E(B3LYP) = -727.279545678 Hartree

Cu	0.94373400	-1.14747500	-0.00873100
N	2.64066200	-0.32775400	-0.85345600
N	-0.73213900	-0.30896700	-0.87480000
C	3.59151700	0.12073400	-1.32137900
C	-1.66080800	0.16218200	-1.36450300
C	-2.83067100	0.75752800	-1.98408200
H	-3.72474000	0.19649200	-1.70590700
H	-2.94048500	1.79151000	-1.65189100
H	-2.72353700	0.74179900	-3.07023800
C	4.79221100	0.68436300	-1.91090800
H	4.56546800	1.65091700	-2.36455800
H	5.55417500	0.82170900	-1.14144100
H	5.17873900	0.01220500	-2.67918100
N	0.93422700	-0.77361800	2.02274300
C	0.92609900	-0.56866500	3.15506400
C	0.91500600	-0.31062600	4.58350500
H	0.44292500	0.65324000	4.78261300
H	0.35576000	-1.09436600	5.09796000
H	1.93731000	-0.29268700	4.96562600
N	0.93888100	-3.18933400	-0.32203800
C	0.93701300	-4.32895800	-0.48167200
C	0.93476300	-5.76687200	-0.68027800

H	1.48206800	-6.01688800	-1.59099000
H	1.41222600	-6.25920100	0.16901500
H	-0.09148500	-6.12757700	-0.77047300

Complex c5 [Cu(OH)(MeCN)]

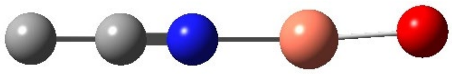

E(B3LYP) = -404.852782366 Hartree

Cu	0.92974100	-0.00716300	-0.03153800
N	-0.94029400	-0.00811600	-0.02258800
C	-2.09068900	-0.00408200	-0.01425300
C	-3.53978200	0.00193900	-0.00288700
H	-3.91493600	0.47879800	-0.91050200
H	-3.91327400	-1.02284300	0.04337500
H	-3.90064500	0.55407200	0.86708200
O	2.75301200	0.11574700	-0.05326900
H	3.19156400	-0.73177600	0.06328500

Complex c6 $[Cu(OH)(MeCN)]_2$

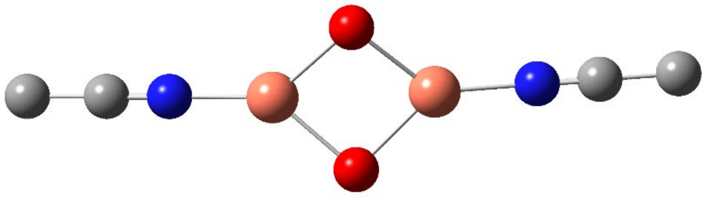

E(B3LYP) = -809.728211233 Hartree

Cu	1.47731800	0.02026700	-0.15228200
N	3.34996100	0.02145700	0.14745200
C	4.49424000	0.01985400	0.29137200
C	5.93468200	0.01724400	0.46822800
H	6.41369700	-0.44147900	-0.39928000
H	6.30320800	1.03937200	0.57734400
H	6.20013800	-0.55338400	1.36057700
O	0.00060800	1.36446100	-0.47305700
H	0.00101100	2.24340600	-0.09009900
O	-0.00053500	-1.33938800	-0.40389000
H	-0.00087800	-2.19795500	0.02281500
Cu	-1.47720200	0.02148600	-0.15209000
N	-3.34981400	0.02442900	0.14782900
C	-4.49408200	0.02395200	0.29184100
C	-5.93451300	0.02275700	0.46880500
H	-6.30196900	1.04521900	0.57839900
H	-6.41406500	-0.43508500	-0.39887000
H	-6.20049700	-0.54798900	1.36092200

Complex c7 [Cu(OH)(PhNHCOMe)]

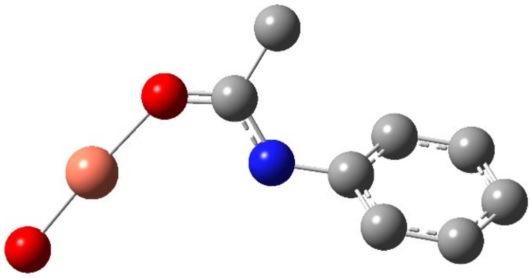

E(B3LYP) = -712.441149962 Hartree

C	0.41891200	2.39694700	0.62728000
H	1.40484800	2.19383800	1.04197000
H	0.54074100	2.99207000	-0.28198800
H	-0.17606800	2.97968100	1.32922600
C	-0.34242700	1.14331100	0.30171200
O	-1.59193900	1.18254900	0.28861500
N	0.31719100	-0.00017200	0.03412500
H	-0.27712900	-0.81106600	-0.11443000
C	1.73301700	-0.21118500	-0.03102500
C	2.31819500	-1.14321400	0.82791000
C	2.51088800	0.46726200	-0.96990000
C	3.68727500	-1.38782900	0.75090800
H	1.70315800	-1.66747100	1.55030000
C	3.88263500	0.23020000	-1.02922200
H	2.04450000	1.16267900	-1.65727900
C	4.47252100	-0.69718600	-0.17167700
H	4.13969700	-2.11301900	1.41698500
H	4.48581300	0.75979300	-1.75724900
H	5.53819900	-0.88588600	-0.22642500
Cu	-2.88673800	-0.17744600	-0.08456600
O	-4.03010500	-1.54402200	-0.51009100
H	-4.87152700	-1.45606000	-0.05195000

Complex c8 [Cu(OH)(PhNHCOMe)]$_2$

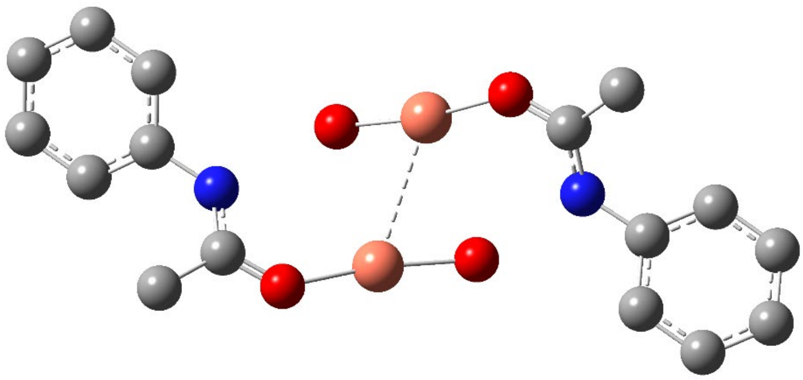

E(RB3LYP) = -1424.93120930 Hartree

Cu	-0.17407300	-1.41905500	-0.23584700
O	-1.51149000	0.94366300	-0.92854700
H	-1.54722100	1.04377100	-1.88485400
O	1.51172600	-0.94347100	-0.92793200
H	1.54755500	-1.04413300	-1.88417700
Cu	0.17427600	1.41953000	-0.23656600
C	3.85623000	2.60283000	1.55407200
H	4.21428300	3.43499900	0.94180500
H	3.28073800	3.02535900	2.37749400
H	4.71734400	2.05848000	1.93746700
C	2.92701400	1.73798000	0.73967000
O	1.73260600	2.12648100	0.63741100
N	3.34317200	0.60301400	0.17341100
H	2.59621100	-0.00976600	-0.29060900
C	4.66760200	0.08382900	0.15981200
C	4.86011200	-1.25556300	0.51572100
C	5.75878100	0.84781300	-0.26534400
C	6.13496700	-1.81316900	0.47082700
H	4.00708200	-1.84892400	0.82200100
C	7.03415100	0.28692500	-0.29558900
H	5.60871100	1.87045700	-0.58908700
C	7.22813800	-1.04320100	0.07328200
H	6.27362700	-2.85138400	0.75034500

H	7.87392000	0.88882400	-0.62402000
H	8.22015300	-1.47861100	0.04198600
C	-3.85616600	-2.60140600	1.55510500
H	-4.21424300	-3.43383100	0.94320000
H	-3.28072800	-3.02359400	2.37873900
H	-4.71726500	-2.05683700	1.93822100
C	-2.92687700	-1.73697500	0.74034100
O	-1.73248000	-2.12557600	0.63832000
N	-3.34296800	-0.60226400	0.17352600
H	-2.59597900	0.01026600	-0.29078500
C	-4.66737400	-0.08301900	0.15968200
C	-4.85982400	1.25652700	0.51504100
C	-5.75858000	-0.84712500	-0.26518200
C	-6.13465200	1.81417600	0.46989700
H	-4.00677000	1.84996800	0.82109800
C	-7.03392200	-0.28618900	-0.29568000
H	-5.60855200	-1.86991000	-0.58850000
C	-7.22785200	1.04409700	0.07264500
H	-6.27326800	2.85251100	0.74898900
H	-7.87371500	-0.88818100	-0.62388000
H	-8.21984600	1.47954100	0.04115400

Complex c9 [(MeCN)Cu(OH), $Ar_2I]^+$

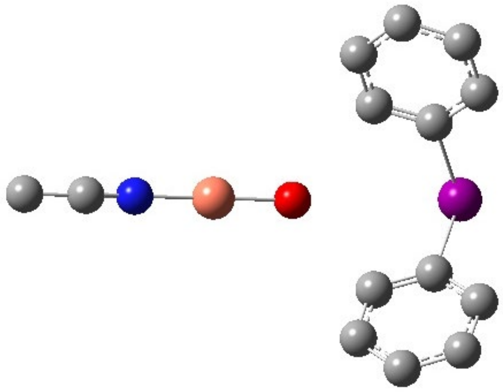

E(B3LYP) = -879.402753433 Hartree

Cu	0.23024500	-0.74564500	2.05986500
I	2.75087300	3.62629400	-0.06430100
C	4.15855600	2.68696600	1.28909000
C	5.31772000	3.38322900	1.60380200
C	3.85010000	1.41592800	1.74650400
C	6.22964400	2.74987900	2.44884200
H	5.51746200	4.37373300	1.21812700
C	4.78424600	0.81299200	2.59334700
H	2.92006500	0.92218800	1.46387300
C	5.96217800	1.47262700	2.94021700
H	7.14564400	3.26224400	2.71754300
H	4.58246500	-0.18112000	2.97477000
H	6.67828200	0.98923600	3.59407500
C	1.26289000	4.35707600	1.33127100
C	0.31098500	3.45466200	1.77837900
C	1.30768800	5.69994500	1.68155700
C	-0.66173700	3.95259200	2.64962200
H	0.33357300	2.40887100	1.47220100
C	0.31937000	6.16361600	2.55041100
H	2.06948000	6.36900800	1.30489900
C	-0.65867100	5.29336800	3.03047100
H	-1.42530100	3.28049600	3.02343600
H	0.32188600	7.20577200	2.84634300

H	-1.42331100	5.66452300	3.70249300
O	0.96881800	0.50512300	0.91921700
H	0.82607300	0.25206300	0.00104600
N	-0.48614600	-1.97054500	3.28075000
C	-0.92830200	-2.73634100	4.01627100
C	-1.48439400	-3.70011500	4.94339000
H	-1.95745000	-3.17895700	5.77810600
H	-2.23054600	-4.31323100	4.43406600
H	-0.69073300	-4.34517600	5.32559500

Complex c10 [(MeCN)Cu(OH), Ar_2I]$^+$

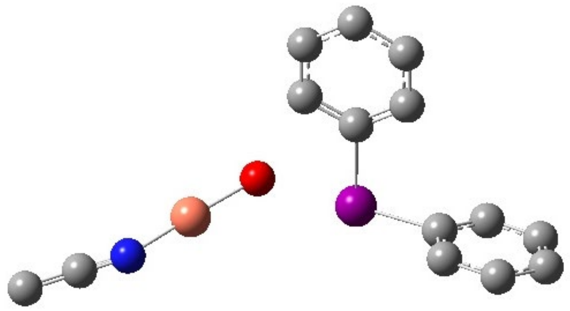

E(B3LYP) = -878.925174258 Hartree

Cu	-1.00876800	-0.79221800	1.88741200
I	2.20984300	1.35781100	1.46202700
C	4.21110800	2.00899300	1.97153600
C	5.02763000	2.54501900	0.97807400
C	4.67904600	1.81929400	3.26983800
C	6.33722300	2.90576700	1.29820100
H	4.65918100	2.68160500	-0.03136400
C	5.98840900	2.18546600	3.57951800
H	4.03968000	1.39415400	4.03383500
C	6.81491400	2.72738800	2.59541200
H	6.97941300	3.32369200	0.53167000
H	6.35951600	2.04157800	4.58749000
H	7.83306300	3.00762000	2.83909100
C	1.28342300	3.22922600	2.14566900
C	-0.08904600	3.23430000	2.31864600
C	2.09299900	4.32859700	2.37723300
C	-0.67853900	4.42465300	2.75188300
H	-0.67286000	2.34845800	2.10298500
C	1.47774800	5.50629900	2.80877300
H	3.16424900	4.29393600	2.23721900
C	0.09829900	5.55467100	2.99669200
H	-1.75205200	4.45424700	2.89747400
H	2.08974900	6.38134000	2.99379100
H	-0.36995900	6.47233300	3.33250200
O	-0.12725300	0.59202700	0.97299200
H	-0.41940000	0.71255000	0.06358500
N	-1.79692900	-2.10649500	2.90897700
C	-2.59802600	-2.93400400	3.21048200
C	-3.48404700	-3.87468100	3.58288300
H	-4.36364700	-3.60306700	4.15021400
H	-3.32620600	-4.91080300	3.31705300

TS-OA

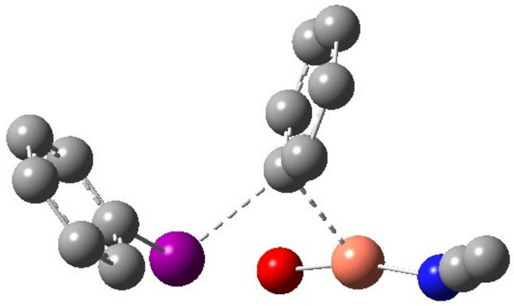

E(B3LYP) = -878.905752293 Hartree

Cu	0.91724000	0.26195100	0.97032000
I	0.56604400	3.13259300	-0.51550200
C	1.19511500	2.36152900	1.80196800
C	2.56939700	2.41754400	2.00648500
C	0.25773000	2.68859700	2.76770000
C	3.01316200	2.72070100	3.29502700
H	3.25916100	2.18986100	1.20522300
C	0.73213100	2.98629900	4.04791000
H	-0.80756400	2.67066600	2.57324000
C	2.10037700	3.00538300	4.31239400
H	4.07931900	2.73332400	3.49357400
H	0.01390700	3.19709400	4.83286500
H	2.45617800	3.24939200	5.30638100
C	-0.41693400	4.93322700	0.12216700
C	-1.80399600	4.94246700	0.26467500
C	0.33939500	6.07405600	0.38751500
C	-2.44041400	6.11551100	0.67042200
H	-2.38663300	4.05293500	0.05903400
C	-0.30691300	7.24055300	0.79492800
H	1.41647600	6.06144500	0.27677000
C	-1.69394200	7.26229200	0.93685700
H	-3.51893900	6.12811400	0.77837500
H	0.27693300	8.13050600	1.00030300
H	-2.19187600	8.17157300	1.25294200
O	1.89285100	0.85062900	-0.54753700
H	1.91820100	0.23881500	-1.28954900
N	-0.17496500	-0.64603500	2.18254600
C	-0.97621800	-0.47515200	3.04579000
C	-1.87997600	-0.28599000	4.01990000
H	-1.57635400	-0.31020600	5.05730400
H	-2.92870700	-0.18120900	3.77807400

TS-OA-bis (cationic)

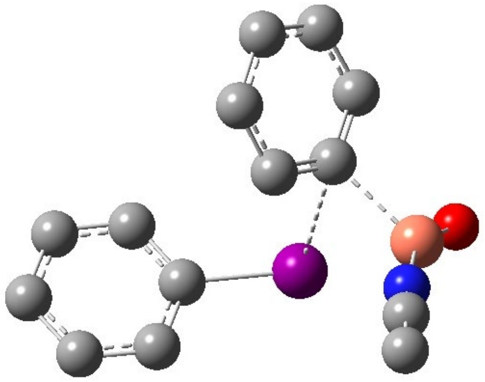

E(B3LYP) = -879.363835776 Hartree

Cu	0.69703600	0.56120400	1.17895200

I	0.53502700	3.24889700	-0.66735400
C	1.53718400	2.29755200	1.92801500
C	2.91525300	2.34943700	1.95431900
C	0.67921200	2.87869400	2.83894500
C	3.47926600	2.96209000	3.08002300
H	3.52142900	1.92895300	1.16666900
C	1.28281100	3.47276800	3.95678400
H	-0.39570100	2.88485700	2.72231200
C	2.66967900	3.51507300	4.07176700
H	4.55946400	2.99922300	3.16327800
H	0.64779400	3.91820100	4.71401200
H	3.12294600	3.99795700	4.92908400
C	-0.50266400	4.93631900	0.10744000
C	-1.88718400	4.87186300	0.26433100
C	0.21639500	6.08473800	0.43591500
C	-2.56125600	5.98813000	0.75816300
H	-2.43677600	3.97927500	-0.00550500
C	-0.47303400	7.19108500	0.92981200
H	1.28983300	6.12521200	0.30498400
C	-1.85731200	7.14425600	1.09223800
H	-3.63811300	5.95131000	0.87426200
H	0.07641500	8.09023500	1.18266100
H	-2.38705000	8.00948200	1.47261200
O	1.81150600	0.61396200	-0.28990400
H	2.45010300	-0.10920300	-0.32354400
N	-0.75074200	-0.07418500	2.34023100
C	-1.57934400	-0.53601100	2.98970100
C	-2.62113600	-1.11720400	3.80825600
H	-2.21331200	-1.95239000	4.38102300
H	-3.01140500	-0.36475500	4.49644500
H	-3.43008000	-1.47842500	3.17014200

Complex c11 [Ph-Cu(OH)(CH_2CN)]

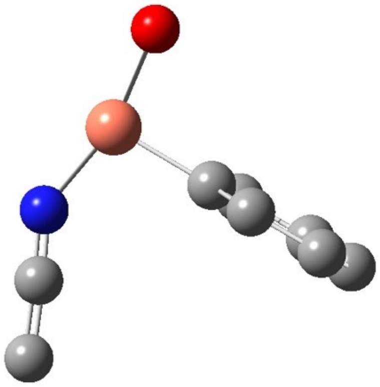

E(B3LYP) = -635.853659128 Hartree

Cu	-0.67681700	2.28610200	0.19212900
N	-1.85480300	0.88909600	0.26433100
C	-1.94463900	-0.30395000	0.24195300
C	0.98406300	1.30274900	-0.03446000
C	1.45342900	1.09552300	-1.32057700
C	1.64523600	0.85498400	1.09683300
C	2.65350200	0.39119300	-1.47508100
H	0.92296200	1.46398500	-2.18983900
C	2.84468200	0.15436500	0.92121600
H	1.26121500	1.03346200	2.09372000
C	3.34432500	-0.07709200	-0.35895100
H	3.03987600	0.21597800	-2.47320200
H	3.37984900	-0.20628800	1.79287000
H	4.27337600	-0.62055900	-0.48677000
C	-2.11249000	-1.62168800	0.22446800

H	-2.09225700	-2.18631700	1.14669500
H	-2.27779600	-2.13809000	-0.71133600
O	0.02887700	3.95018800	0.17208600
H	0.98822600	4.01313200	0.15105800

TS-RE

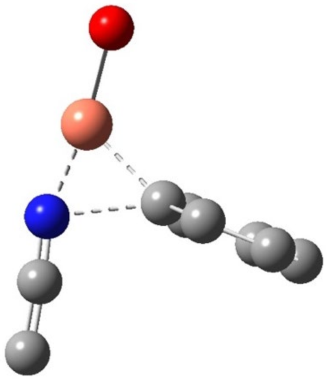

E(B3LYP) = -635.845186328 Hartree

Cu	-0.72476300	2.30696200	0.21532000
N	-1.44945600	0.62097900	0.22784200
C	-1.58558000	-0.57271400	0.25317600
C	0.67826700	0.86727900	-0.00647800
C	1.12371600	0.64181300	-1.29774200
C	1.40353000	0.53952700	1.12642200
C	2.40902300	0.11498800	-1.45552400
H	0.51201900	0.87548600	-2.15981300
C	2.68658100	0.01435600	0.94436700
H	1.00514900	0.69094200	2.12171500
C	3.18668200	-0.19583900	-0.34022800
H	2.79311000	-0.05208200	-2.45564100
H	3.28614900	-0.23169500	1.81368500
H	4.17837800	-0.61214200	-0.47242400
C	-1.80918300	-1.87363400	0.28339300
H	-1.84578800	-2.39886200	1.22837400
H	-1.97504100	-2.41990600	-0.63548600
O	-0.27315400	4.07061600	0.19300100
H	0.60248600	4.27796400	0.52978200

Ketenimide (H_2CCNPh)

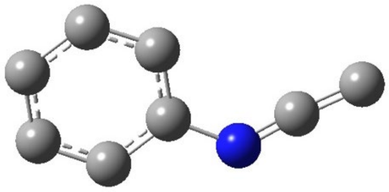

E(B3LYP) = -363.866000505 Hartree

C	-2.60281100	0.26512300	0.12007400
C	-2.23187400	-1.05820800	-0.11262700
C	-0.88450800	-1.40923700	-0.16216700
C	0.09836300	-0.43422800	0.02049000
C	-0.27189400	0.89563900	0.25454400
C	-1.61883600	1.23921900	0.30332000
H	-3.65099200	0.53825300	0.15903300
H	-2.99047800	-1.81915700	-0.25550900
H	-0.57792400	-2.43270300	-0.34152000
H	0.49389700	1.65005700	0.39656800
H	-1.90261100	2.26975700	0.48488200
N	1.45267600	-0.86174300	-0.04235800

C	2.44371500	-0.15741700	0.07645900
C	3.56615700	0.50777400	0.18905400
H	4.03233900	0.64100800	1.15759700
H	4.04166300	0.93700100	-0.68433200

Complex c12 $[Cu(OH)_2]^-$

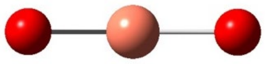

E(B3LYP) = -348.005466163 Hartree

Cu	-0.61696700	0.88688900	0.00000000
O	1.24827000	0.88196100	-0.00198000
H	1.56507400	0.03902700	-0.34064800
O	-2.48220300	0.89181800	0.00198000
H	-2.79900700	1.73475200	0.34064800

Complex c13 $[Cu(OH)(CH_2CN)]^-$

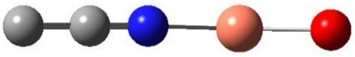

E(B3LYP) = -404.332356672 Hartree

Cu	0.90058400	-0.09818200	-0.20926800
N	-0.95249200	-0.04371900	-0.19011100
C	-2.13265800	-0.04305600	-0.07598800
C	-3.47961000	-0.04420300	0.04944000
H	-4.00397600	0.85888600	0.33169000
H	-4.04540200	-0.94243700	-0.15924300
O	2.75036200	-0.12274700	-0.32325100
H	3.13001700	-0.36826900	0.52553800

Complex 14 $[Cu(OH)_2, Ar_2I]$

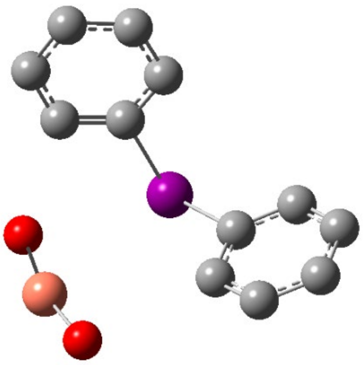

E(B3LYP) = -822.594861217 Hartree

Cu	-0.68534000	0.17620000	1.14573600
I	1.95142700	2.26985700	0.26237200
C	3.34708600	1.52948100	1.73576100
C	4.63226100	2.06228400	1.74470700
C	2.91142800	0.52568300	2.58893500
C	5.53095000	1.56707400	2.68893700
H	4.93654300	2.82889300	1.04415000
C	3.83817900	0.05318100	3.52375300
H	1.90689900	0.08997100	2.54855200
C	5.13133400	0.56889200	3.57745000
H	6.53916600	1.96289300	2.72182100
H	3.53063300	-0.72899200	4.20793100
H	5.83474500	0.18767500	4.30864800
C	1.01214800	3.84799400	1.42928800

C	-0.37019500	3.85000800	1.45771900
C	1.81847800	4.78619700	2.05595000
C	-0.98022200	4.88119400	2.17974100
H	-0.93801500	3.07687500	0.93348400
C	1.17901300	5.79394600	2.77825800
H	2.89813900	4.74802700	2.00285900
C	-0.21428500	5.84057000	2.83741300
H	-2.06239000	4.91683100	2.22852000
H	1.77687400	6.54042600	3.28777300
H	-0.70157500	6.62972300	3.39806600
O	0.11003800	-0.79515200	2.52064700
H	-0.10001000	-1.73249300	2.47418700
O	-1.14066400	1.42219700	-0.17847200
H	-1.99798300	1.26134100	-0.58357000

Complex TS-OA-2

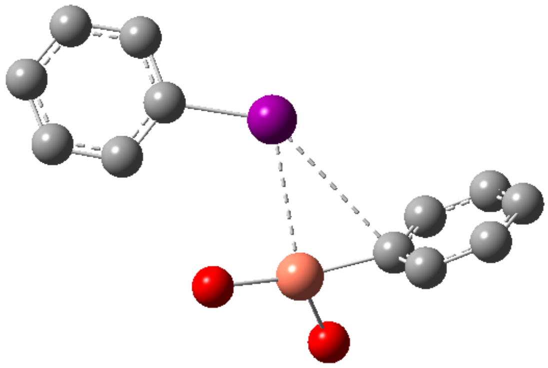

E(B3LYP) = -822.600061754 Hartree

Cu	0.32216000	1.68241400	1.30051600
I	1.15349800	4.71195600	1.29019400
C	2.03869600	1.36440700	2.10269200
C	3.23985600	1.65627000	1.47439500
C	1.95275400	0.95882600	3.42660700
C	4.40772300	1.61483900	2.24290300
H	3.28067100	1.92026400	0.42477400
C	3.13237600	0.92587500	4.17961900
H	1.00793000	0.68400000	3.88123000
C	4.35137100	1.25340900	3.58881700
H	5.35689400	1.86141900	1.78047300
H	3.08779000	0.63207300	5.22228300
H	5.26224200	1.21295000	4.17478900
C	-0.49040600	5.88275800	0.54588800
C	-1.73593800	5.28420600	0.40084400
C	-0.25848100	7.21790300	0.23147900
C	-2.78521000	6.06981400	-0.08356600
H	-1.88053400	4.23663600	0.65290300
C	-1.32249900	7.98190300	-0.25021300
H	0.72164000	7.66125800	0.35226900
C	-2.58416000	7.41032200	-0.40788200
H	-3.76455700	5.62059900	-0.20587500
H	-1.15679600	9.02352900	-0.50128100
H	-3.40669300	8.00835500	-0.78310900
O	0.72945300	0.15675900	0.36483300
H	1.55902000	-0.27302400	0.60048100
O	-1.44264300	2.26109000	1.22673600
H	-2.10756600	1.57286700	1.32526100

Complex TS-OA-3

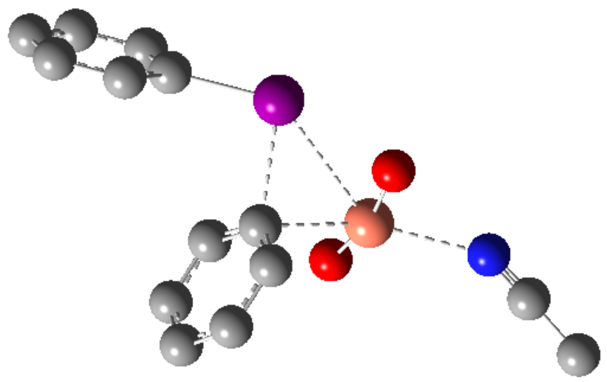

E(B3LYP) = -955.382473300 Hartree

Cu	0.61796800	0.50602300	0.60136900
I	0.60426400	3.50599900	-0.63143500
C	1.06687600	2.54999600	1.58864800
C	2.42893200	2.49920600	1.85959800
C	0.09798800	2.81463300	2.54179000
C	2.82250200	2.63575300	3.19197700
H	3.14321600	2.31946200	1.06834800
C	0.52352800	2.94875200	3.86473200
H	-0.95313300	2.84702300	2.29440800
C	1.87596600	2.86328900	4.19187300
H	3.87684100	2.56754600	3.43744500
H	-0.21952300	3.11159600	4.63754400
H	2.19256100	2.97747900	5.22205900
C	-0.28381400	5.31163900	0.13818900
C	-1.66846100	5.38672500	0.28236900
C	0.52996400	6.39209600	0.47518300
C	-2.24316400	6.56292600	0.76463700
H	-2.29663800	4.54322600	0.02327000
C	-0.05366300	7.56299500	0.95832200
H	1.60531400	6.32897800	0.36318000
C	-1.43786900	7.64927200	1.10306400
H	-3.31971600	6.62499900	0.87621500
H	0.57624300	8.40549200	1.22054900
H	-1.88769100	8.56124200	1.47835200
O	1.75770000	1.20744900	-0.78192100
H	1.77464400	0.65659100	-1.57124400
O	-0.71601400	0.14285800	1.89119800
H	-1.08179200	-0.73373900	1.73356300
N	0.91061600	-1.79872800	-0.07152000
C	1.22452600	-2.86716400	0.22605300
C	1.62105700	-4.21004200	0.61610100
H	2.60615100	-4.44217700	0.20747800
H	1.66210500	-4.28136700	1.70476400
H	0.89940200	-4.93746400	0.23995100

Complex c16 [Cu(OH)$_2$Ph, PhI]

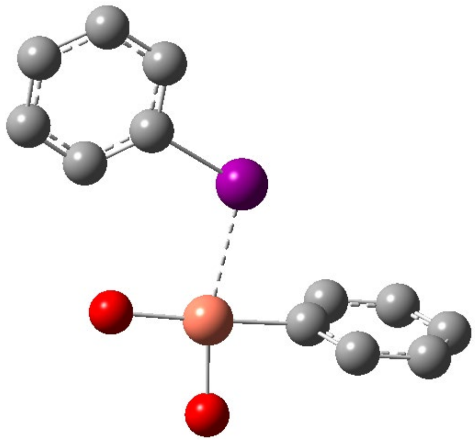

E(B3LYP) = -822.600326891 Hartree

Cu	0.35952900	0.77426100	0.75099000
C	1.85736100	1.09435000	1.91183800
C	2.90330800	1.85357600	1.42185700
C	1.81661600	0.54825800	3.18089800
C	3.99062100	2.07302400	2.27640700
H	2.88730600	2.27992600	0.42801700
C	2.91212500	0.79129700	4.01834300
H	0.98690900	-0.05752300	3.51901700
C	3.99247700	1.54673200	3.56669800
H	4.82612400	2.66717600	1.92360800
H	2.91260200	0.37418000	5.01912000
H	4.83737000	1.72554200	4.22143900
O	0.62790700	-0.98886700	0.58140400
H	1.54047700	-1.25617200	0.43197000
O	-0.20074400	2.47490600	0.69432800
H	-0.21008900	2.93633300	1.53902900

Complex c15 $[Cu(OH)_2Ph]$

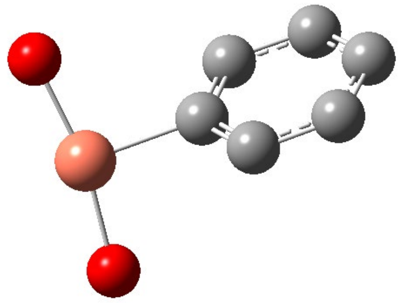

E(B3LYP) = -579.531921423 Hartree

Cu	0.35952900	0.77426100	0.75099000
C	1.85736100	1.09435000	1.91183800
C	2.90330800	1.85357600	1.42185700
C	1.81661600	0.54825800	3.18089800
C	3.99062100	2.07302400	2.27640700
H	2.88730600	2.27992600	0.42801700
C	2.91212500	0.79129700	4.01834300
H	0.98690900	-0.05752300	3.51901700
C	3.99247700	1.54673200	3.56669800
H	4.82612400	2.66717600	1.92360800
H	2.91260200	0.37418000	5.01912000
H	4.83737000	1.72554200	4.22143900
O	0.62790700	-0.98886700	0.58140400
H	1.54047700	-1.25617200	0.43197000
O	-0.20074400	2.47490600	0.69432800

H	-0.21008900	2.93633300	1.53902900

Complex 17 [$Cu(OH)_2(MeCN)Ph$]

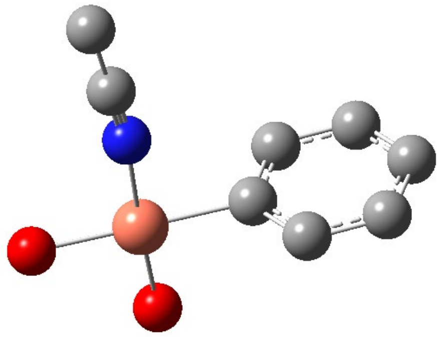

E(B3LYP) = -712.331755997 Hartree

Cu	0.25345400	1.68989200	1.50328000
C	2.01451800	1.36817200	2.27913800
C	3.13821600	1.87354500	1.63917000
C	2.10522200	0.68412800	3.48391700
C	4.39283700	1.69102000	2.23123100
H	3.05907500	2.39612100	0.69227700
C	3.36437000	0.50707300	4.06790600
H	1.22320500	0.28200700	3.96998400
C	4.50458300	1.01074500	3.44318000
H	5.27789100	2.07977200	1.73902900
H	3.44721900	-0.02761200	5.00808600
H	5.47891600	0.86776700	3.89656800
O	0.45985500	0.10642200	0.61651900
H	1.30915900	-0.29612000	0.82685300
O	-1.45271400	2.01684900	0.76592600
H	-1.62804400	1.26139400	0.19329200
N	0.10696700	3.36107500	2.48018200
C	-0.10093300	4.36090200	3.00118600
C	-0.37313400	5.62387800	3.65598600
H	-0.88226100	6.29257100	2.95953000
H	-1.01553400	5.45398600	4.52215200
H	0.56104300	6.08310300	3.98377000

Complex 18 [$Cu(OH)_2Ph(CH_2CN)$]

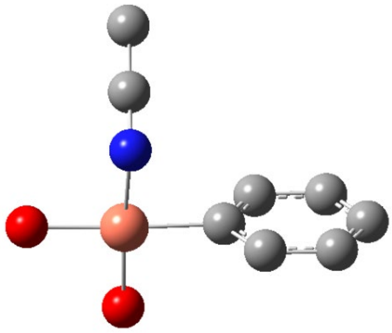

E(B3LYP) = -711.824844710 Hartree

Cu	0.07921500	1.67136800	1.67110000
C	1.85228500	1.45972800	2.45227400
C	2.94317100	2.13312500	1.90453900
C	2.04080300	0.61248000	3.54331700
C	4.22162200	1.95607800	2.44446600
H	2.81088900	2.79735900	1.05633000
C	3.31925200	0.43107600	4.07887100

H	1.19790100	0.09300500	3.98692300
C	4.41283000	1.10278800	3.53108300
H	5.06581100	2.48515000	2.01259100
H	3.45770400	-0.23174400	4.92778200
H	5.40446200	0.96325200	3.94851400
O	0.51252100	0.40601900	0.35341700
H	1.39785300	0.06999600	0.52319800
O	-1.62058000	1.97508700	0.87141900
H	-1.59320600	1.38278900	0.11076000
N	-0.34160300	2.84973400	3.14026400
C	-0.12954200	4.02930100	3.10189100
C	0.07560800	5.34867000	3.14103900
H	-0.63856700	6.02612300	2.69267800
H	0.96333600	5.75082000	3.61118500

Complex I-7-trans

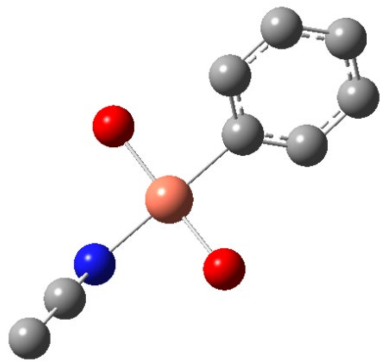

Cu	-0.13989100	1.83207100	1.71178600
C	0.67292800	3.49082000	1.06289000
C	1.86418600	3.47278300	0.34002800
C	0.01895200	4.69715500	1.30003000
C	2.40667000	4.66983300	-0.14281200
H	2.37755200	2.53693100	0.14909700
C	0.57339800	5.89259100	0.83482400
H	-0.93075100	4.70436400	1.82104100
C	1.76686400	5.88188500	0.11066200
H	3.32988900	4.64898700	-0.71408200
H	0.06436200	6.83193400	1.02924800
H	2.19078900	6.81059200	-0.25700900
O	1.46074000	1.35876200	2.53368400
H	2.03407200	2.13090000	2.55530300
O	-1.76700700	2.42815800	0.99394300
H	-2.30353900	1.62834400	1.05069500
N	-0.98344300	0.06995200	2.16204800
C	-1.17930700	-0.31397400	3.27684000
C	-1.42427300	-0.80108700	4.50367500
H	-2.36369200	-0.59454300	4.99847700
H	-0.66642900	-1.37103100	5.02361700

TS-RE-2

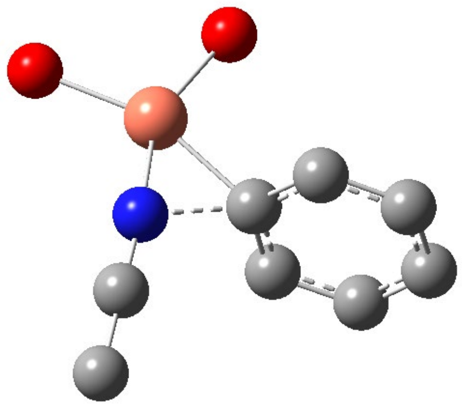

E(B3LYP) = -711.799015331 Hartree

Cu	-0.80145700	2.18824800	0.32546400
N	-1.09228700	0.30438300	0.65015900
C	-1.37075600	-0.81377700	0.30074700
C	0.65340900	0.80627000	0.15996200
C	1.04200100	0.48103900	-1.14206600
C	1.55588600	0.72936800	1.22174700
C	2.37125400	0.13928600	-1.38472500
H	0.32420400	0.50756400	-1.95329200
C	2.87905400	0.37894300	0.96293200
H	1.23251900	0.96052800	2.22917600
C	3.29445000	0.08589400	-0.33818400
H	2.68494300	-0.08750100	-2.39862300
H	3.58917000	0.34242000	1.78286100
H	4.32592300	-0.18484600	-0.53375500
C	-1.66972200	-2.06475800	-0.02289100
H	-1.19724300	-2.88997400	0.49456700
H	-2.40610000	-2.27306400	-0.78756000
O	0.33335000	3.72723300	0.24948800
H	-0.30925200	4.44539900	0.22308100
O	-2.57387600	3.02547200	0.20957800
H	-3.23939300	2.41644400	0.54577400

COPIES OF ^{1}H AND ^{13}C NMR SPECTRA OF NEW COMPOUNDS.

^{1}H NMR (250 MHz, $CDCl_3$) Analysis of 3aa.

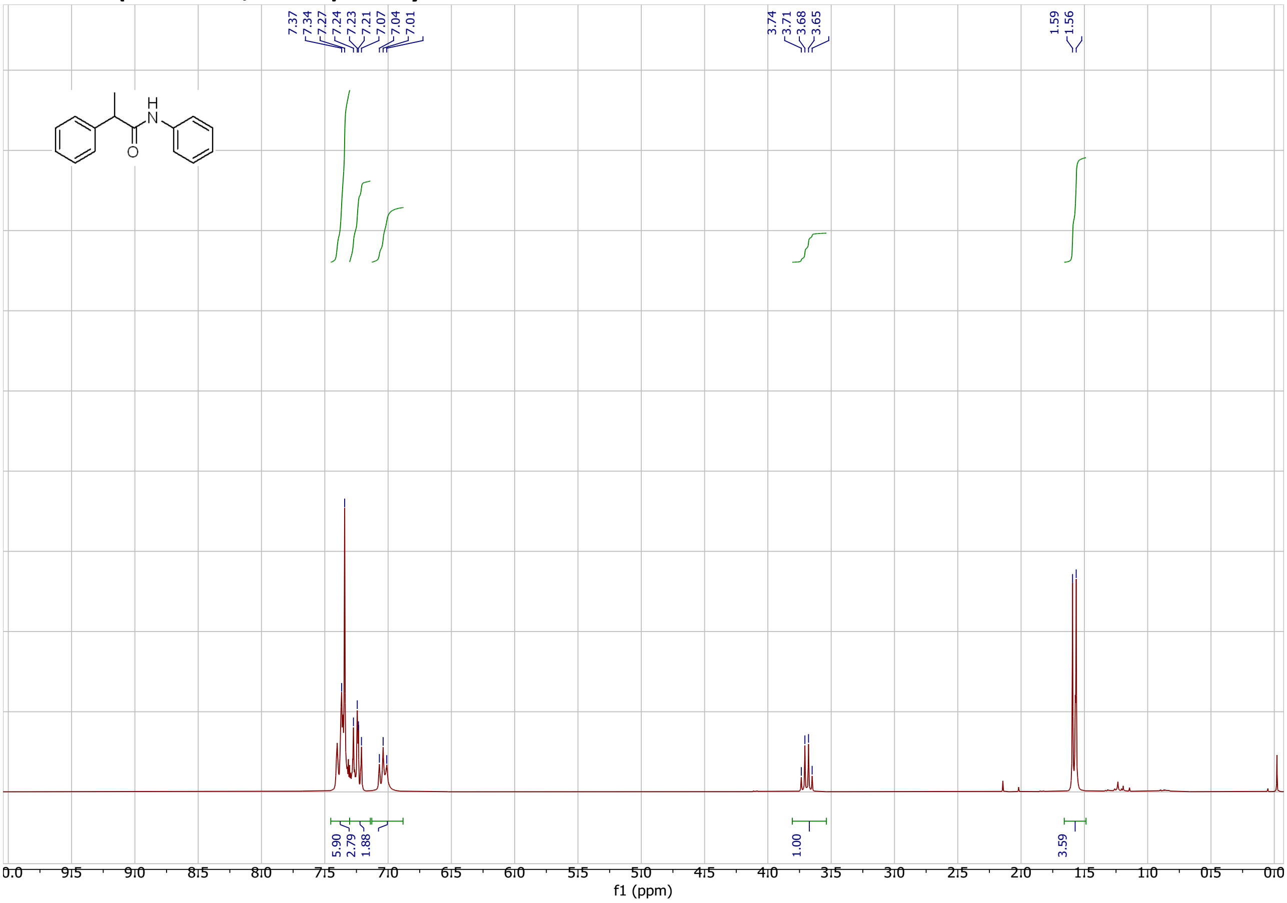

^{13}C NMR (75 MHz) Analysis of 3aa

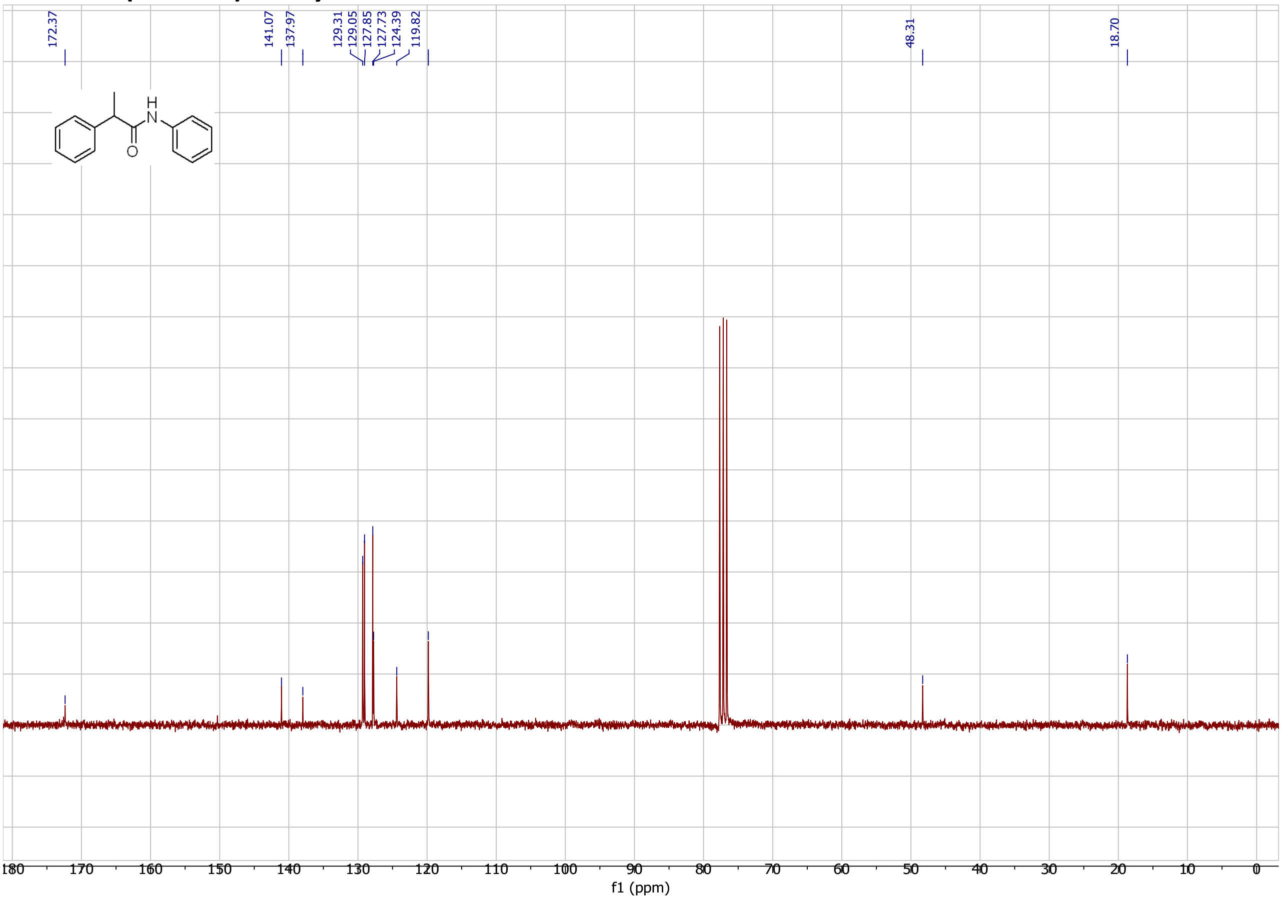

^{1}H NMR (400 MHz, $CDCl_3$) Analysis of 3ba.

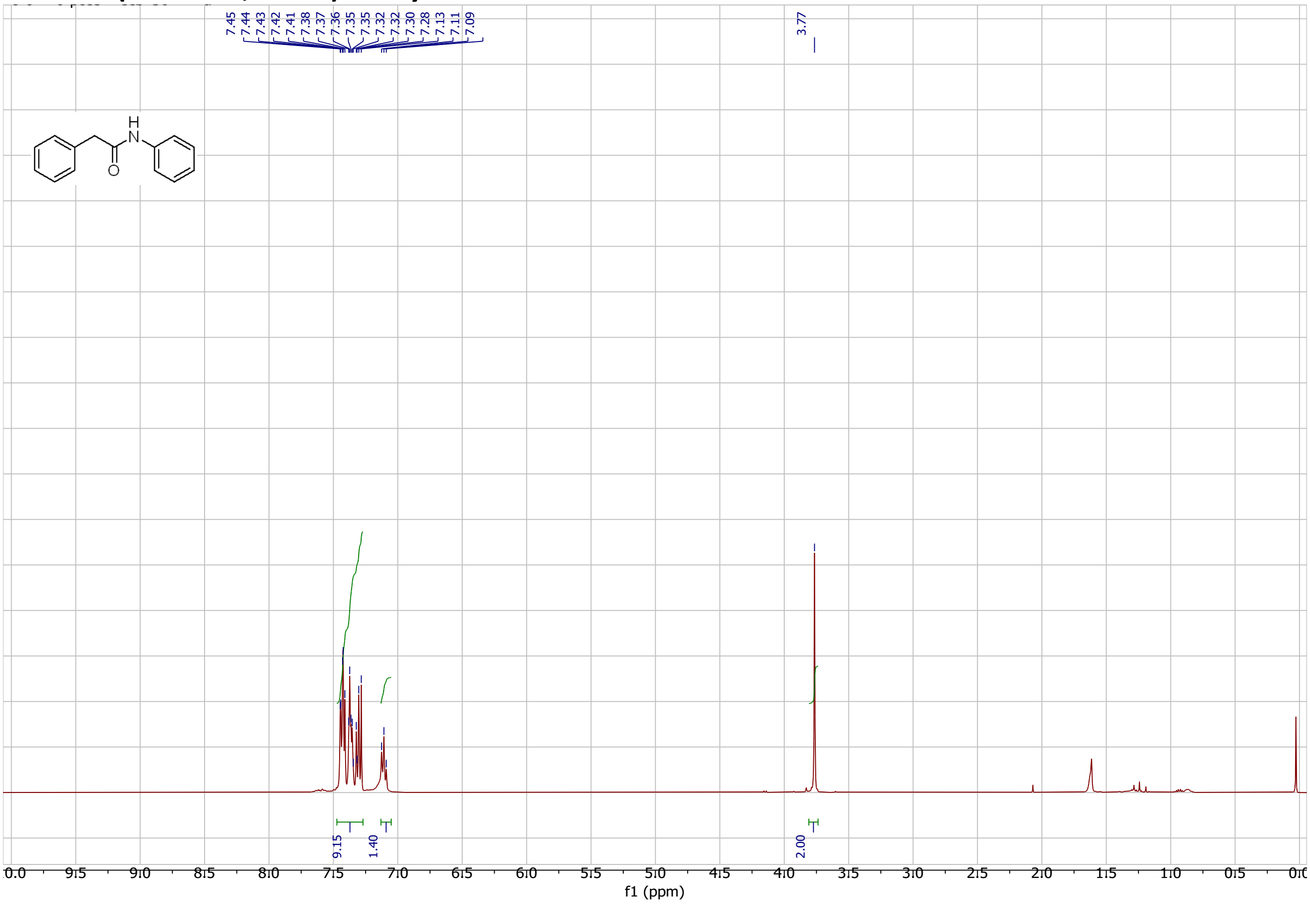

^{13}C NMR (101 MHz, $CDCl_3$) Analysis of 3ba.

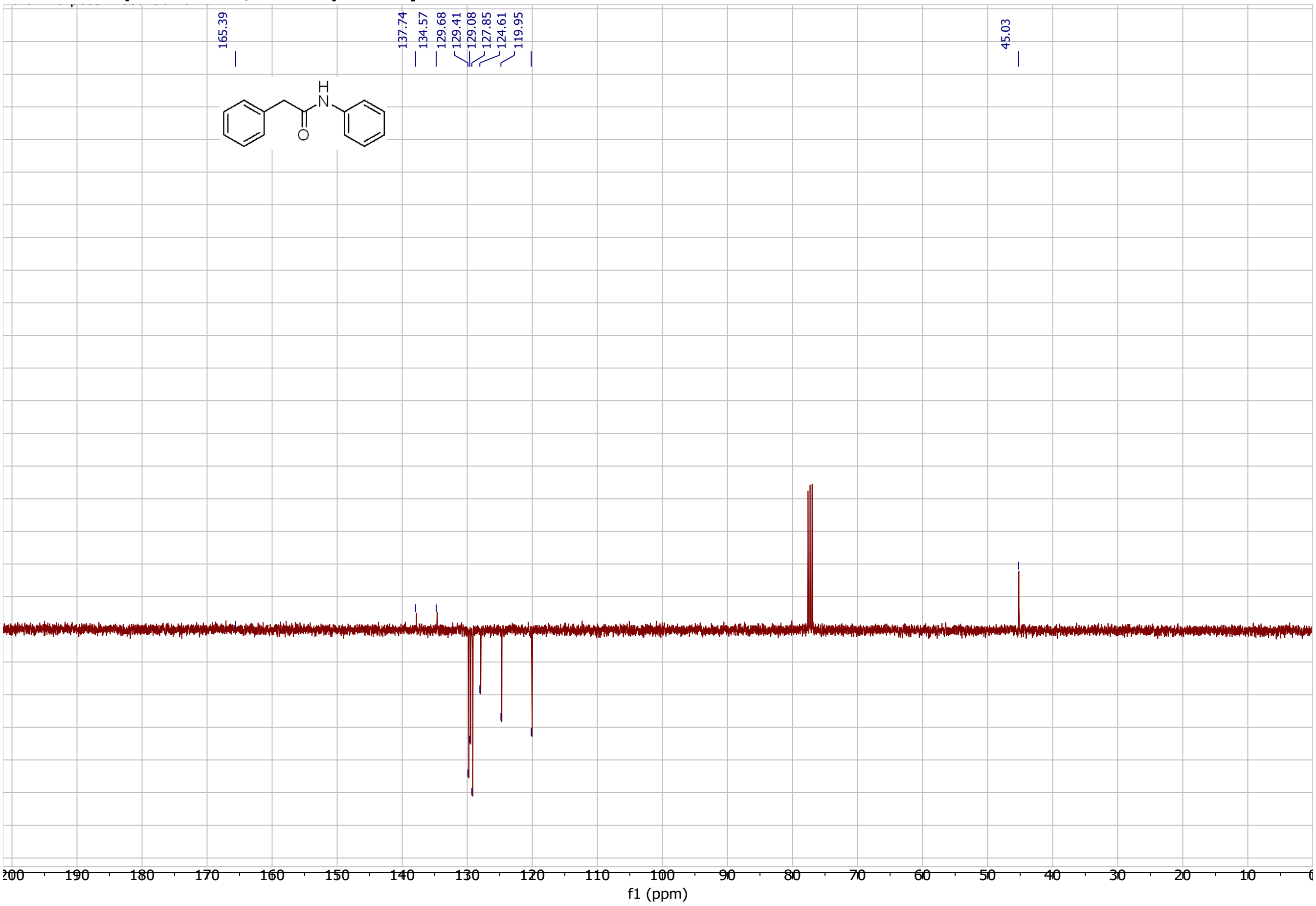

^{1}H NMR (400 MHz, $CDCl_3$) Analysis of 3ca.

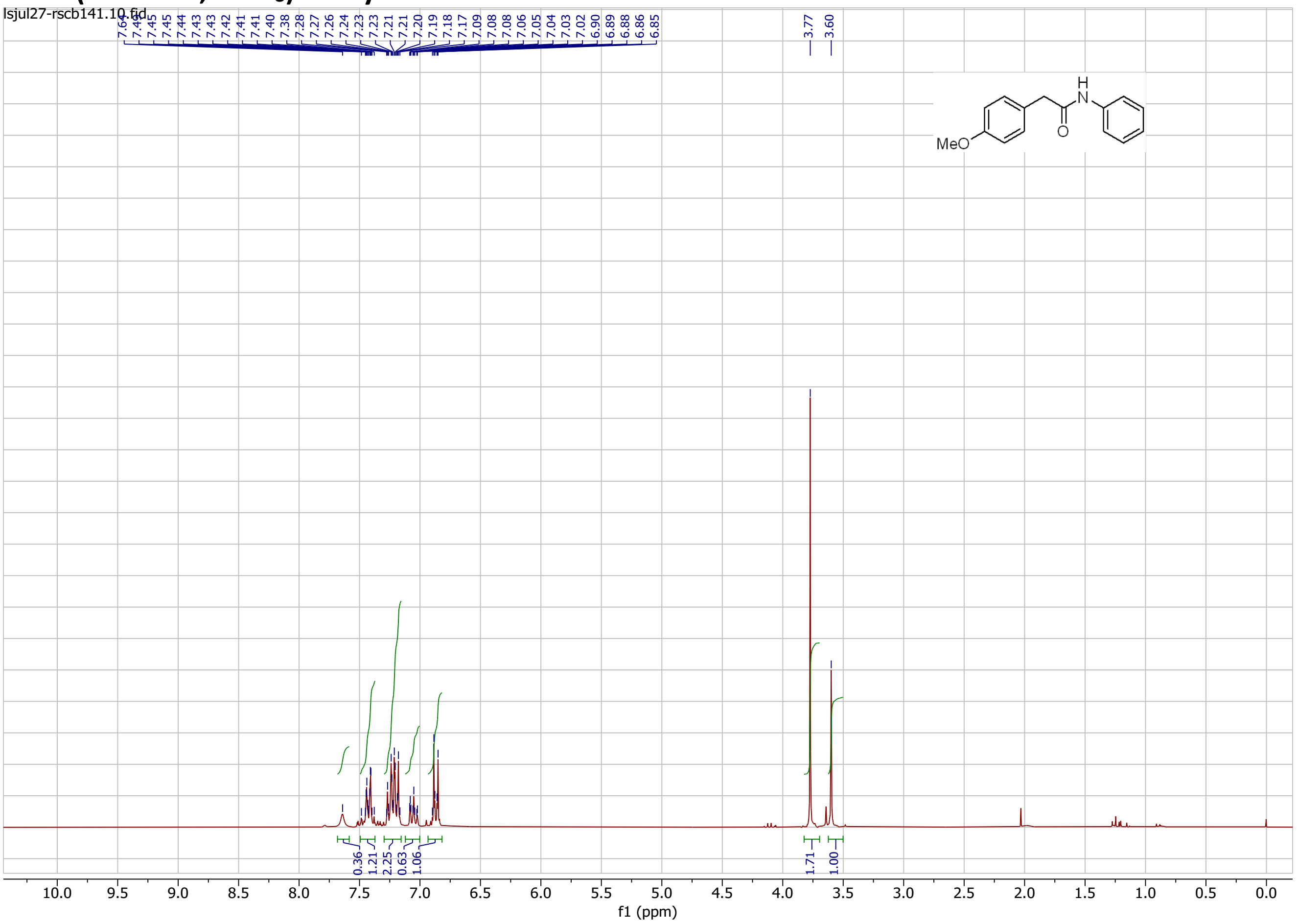

^{13}C NMR (101 MHz, $CDCl_3$) Analysis of 3ca.

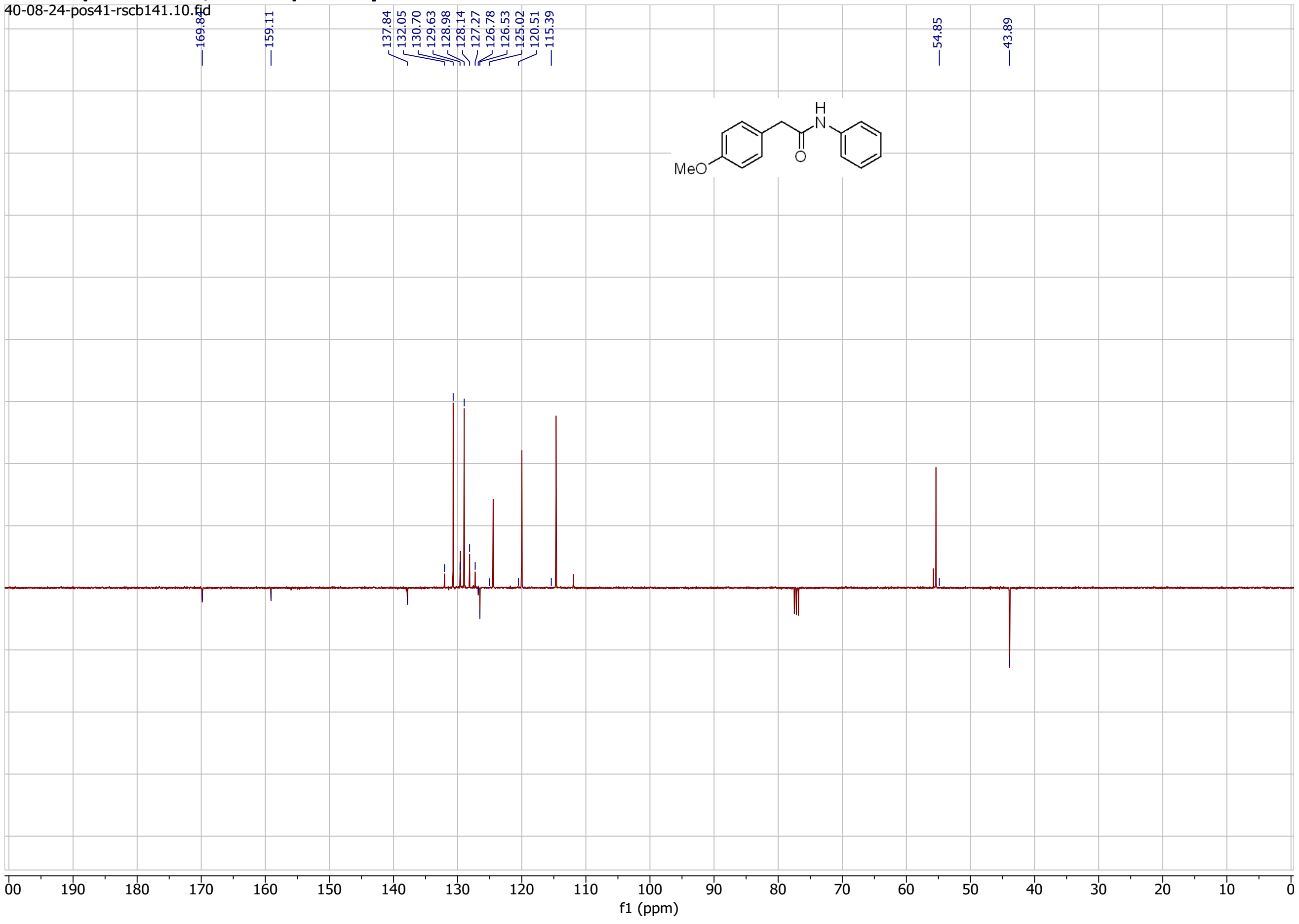

^{1}H NMR (400 MHz, $CDCl_3$) Analysis of 3da.

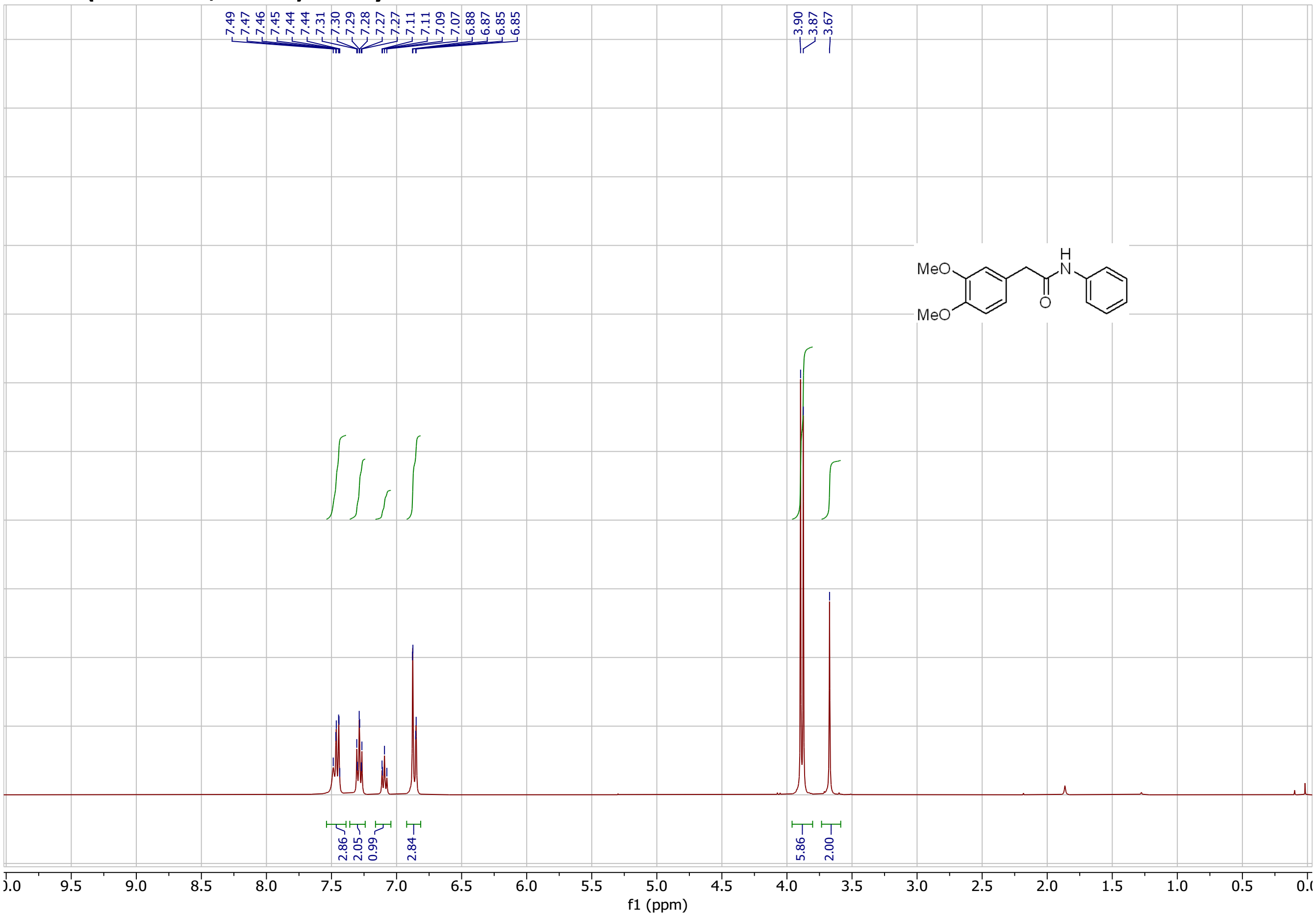

^{13}C NMR (101 MHz, $CDCl_3$) Analysis of 3da.

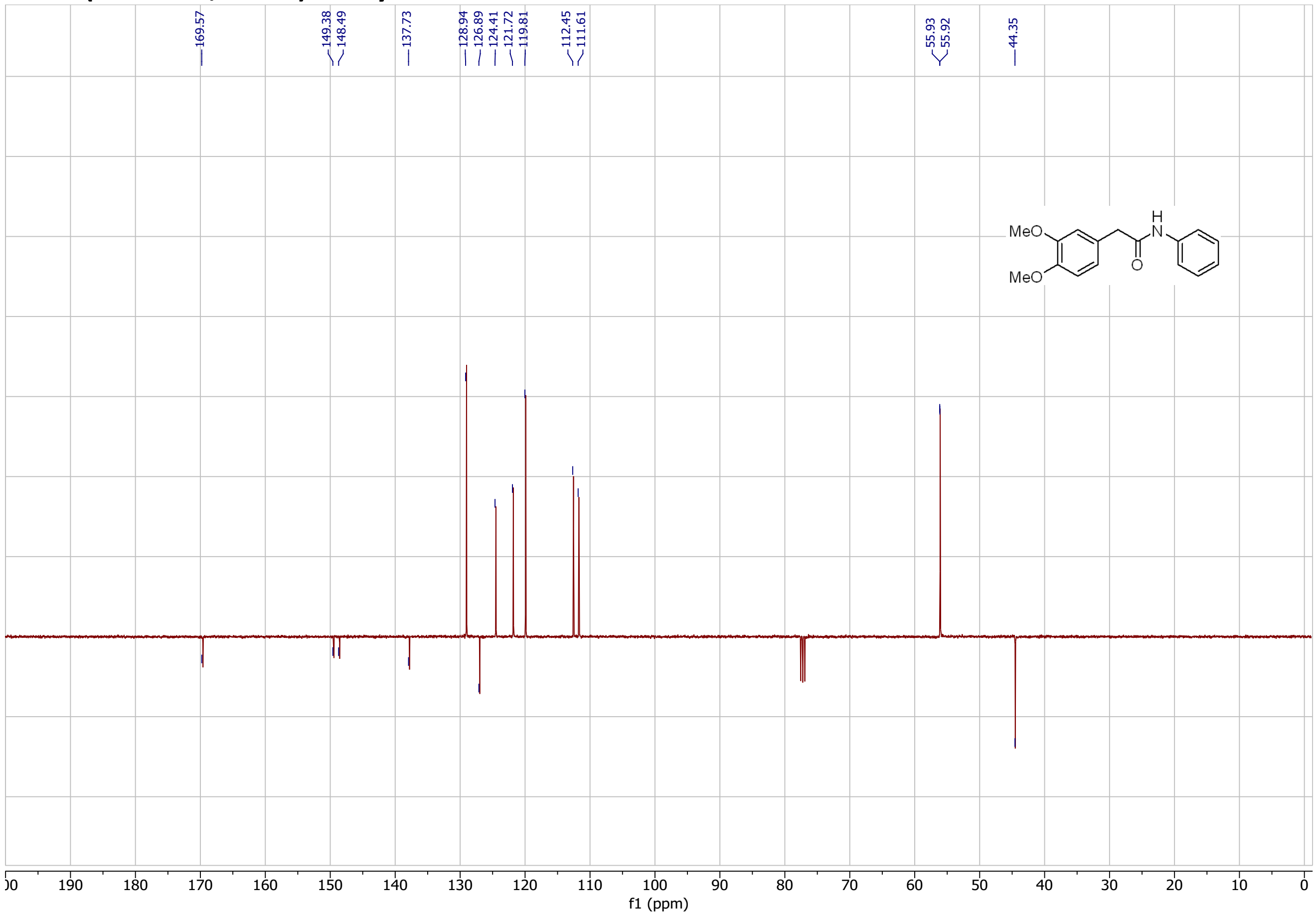

^1H NMR (400 MHz, $CDCl_3$) Analysis of 3ea.

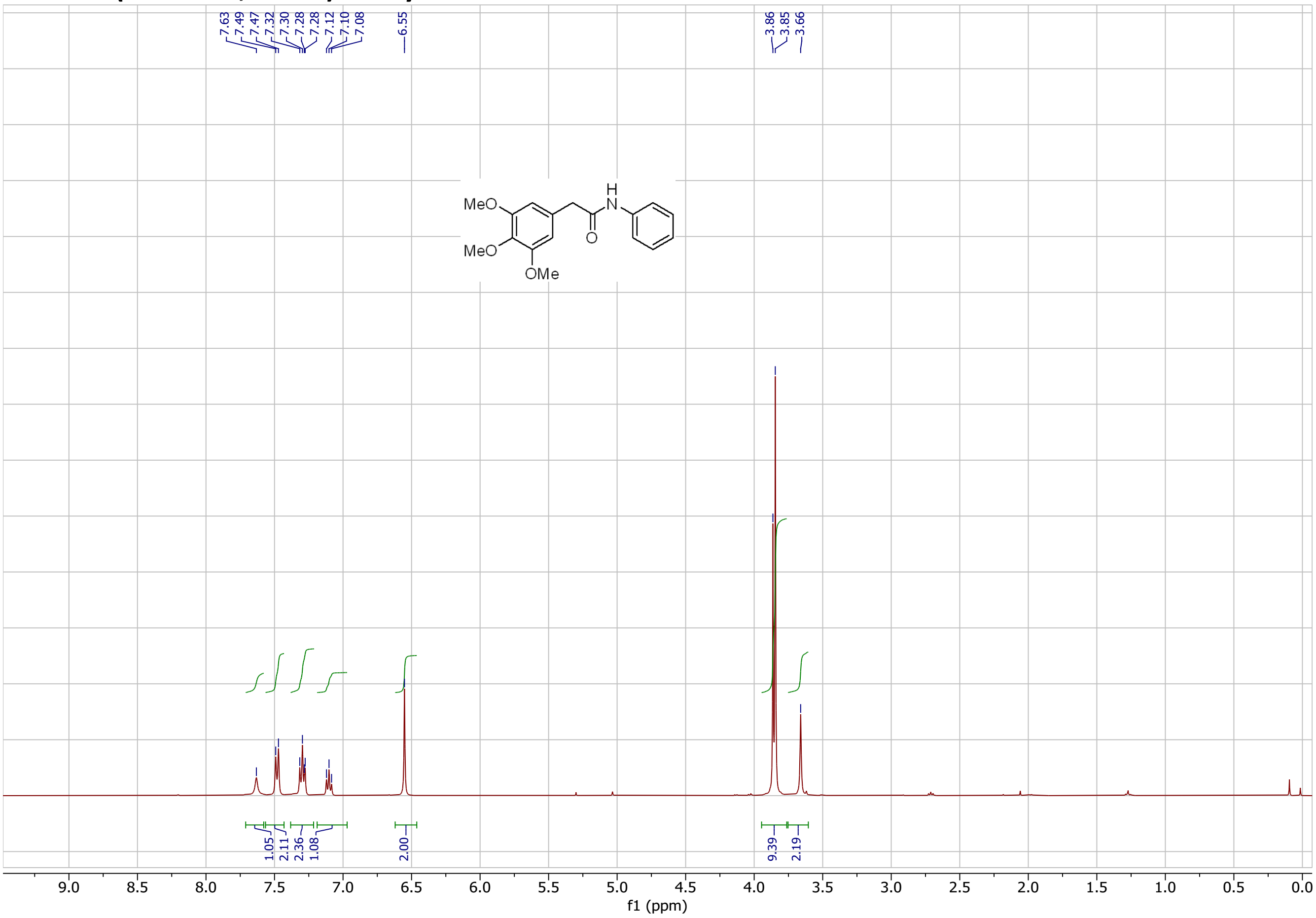

^{13}C NMR (101 MHz, $CDCl_3$) Analysis of 3ea.

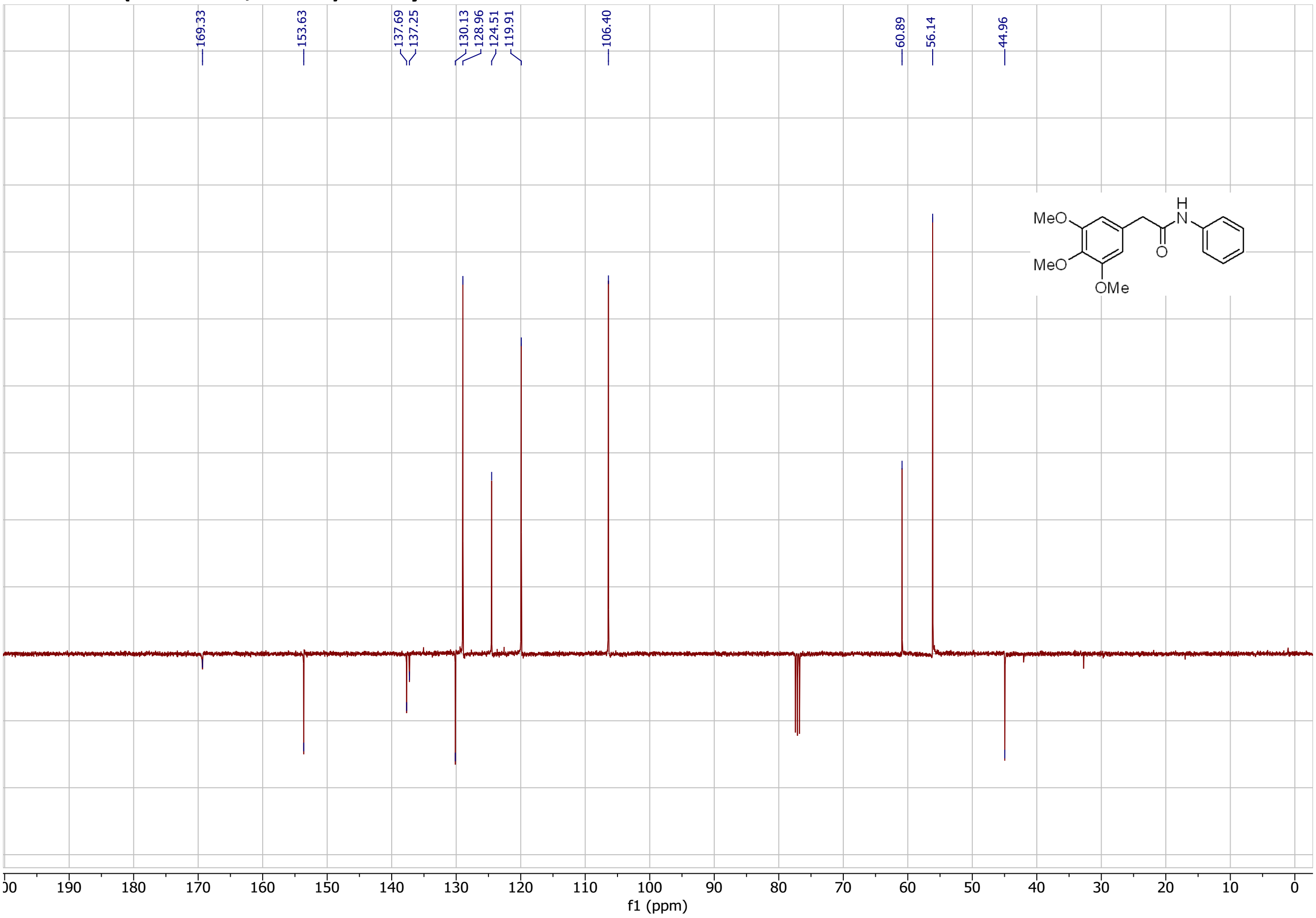

^{1}H NMR (400 MHz, DMSO-d_6) Analysis of 3fa.

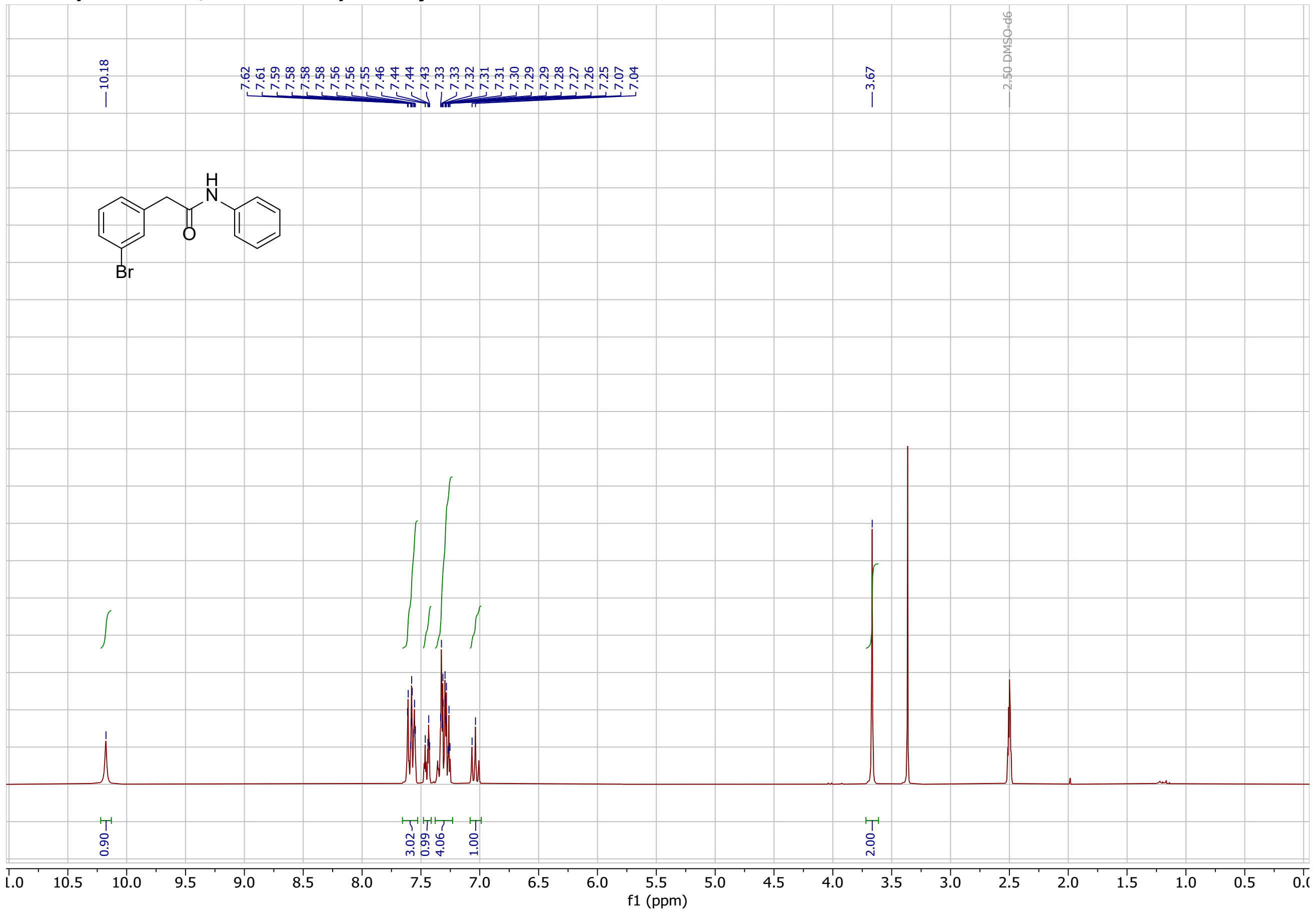

^{13}C NMR (101 MHz, DMSO-d_6) Analysis of 3fa.

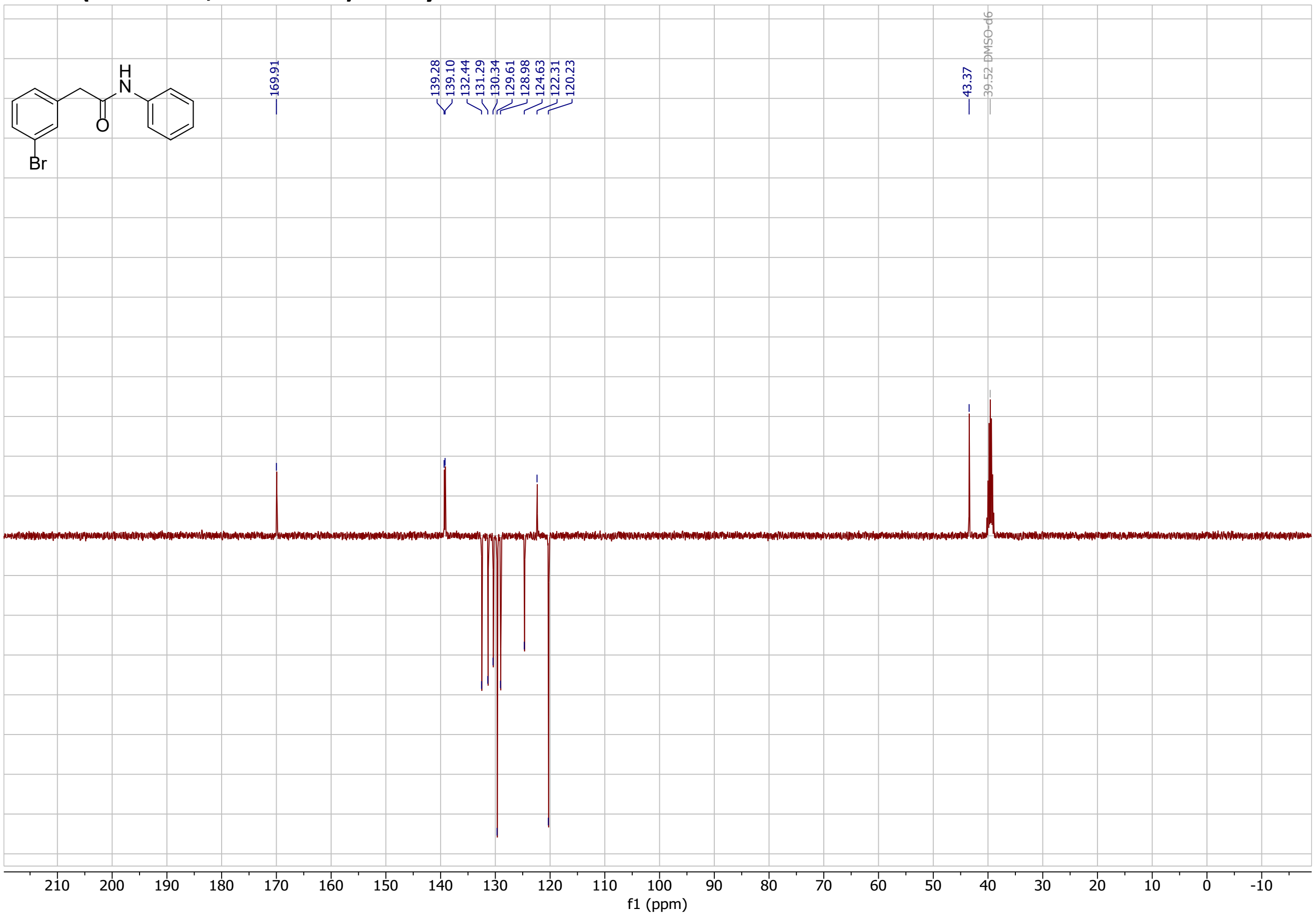

^{1}H NMR (400 MHz, CDCl$_3$) Analysis of 3ga.

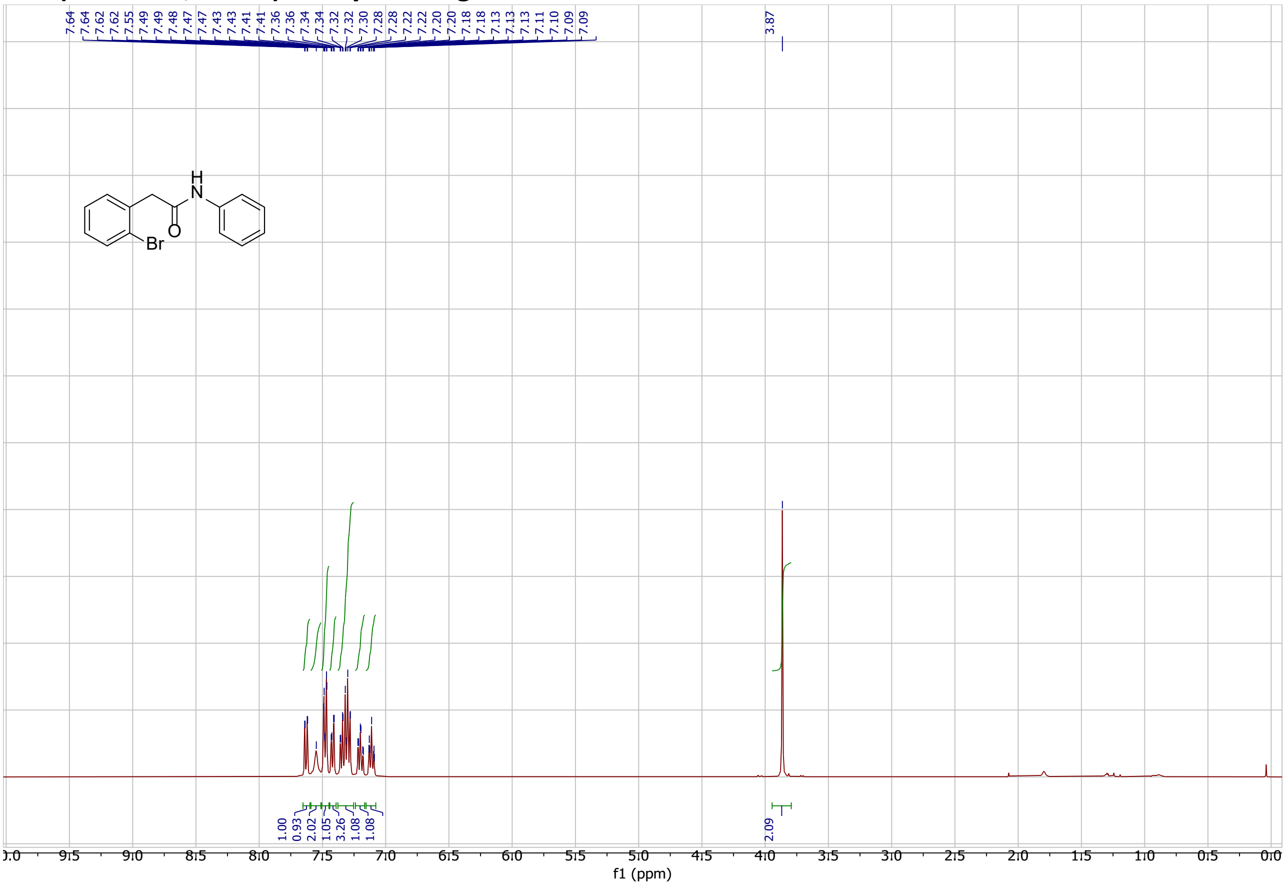

^{13}C NMR (101 MHz, CDCl$_3$) Analysis of 3ga.

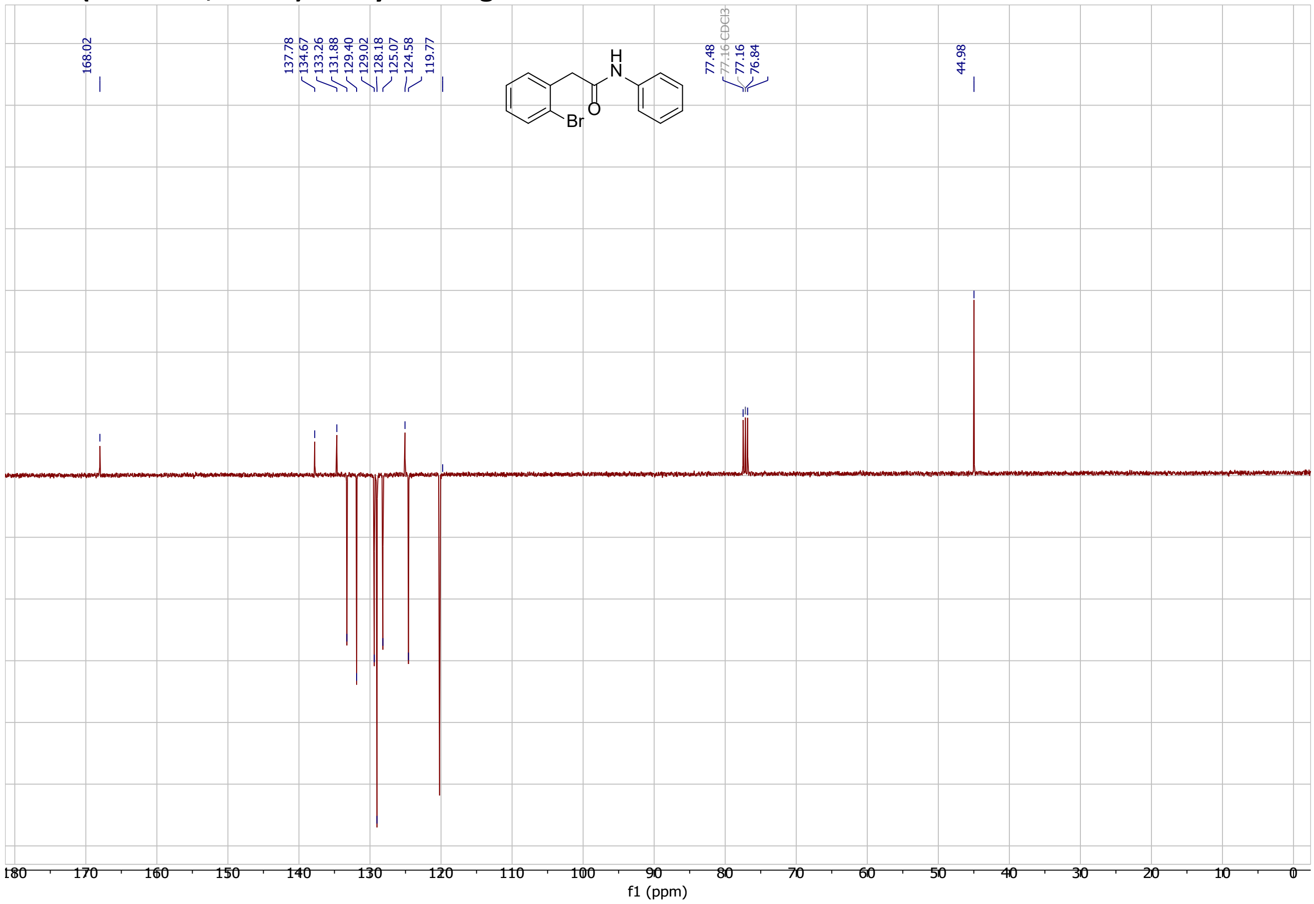

^{1}H NMR (250 MHz, CDCl$_3$) Analysis of 3ha.

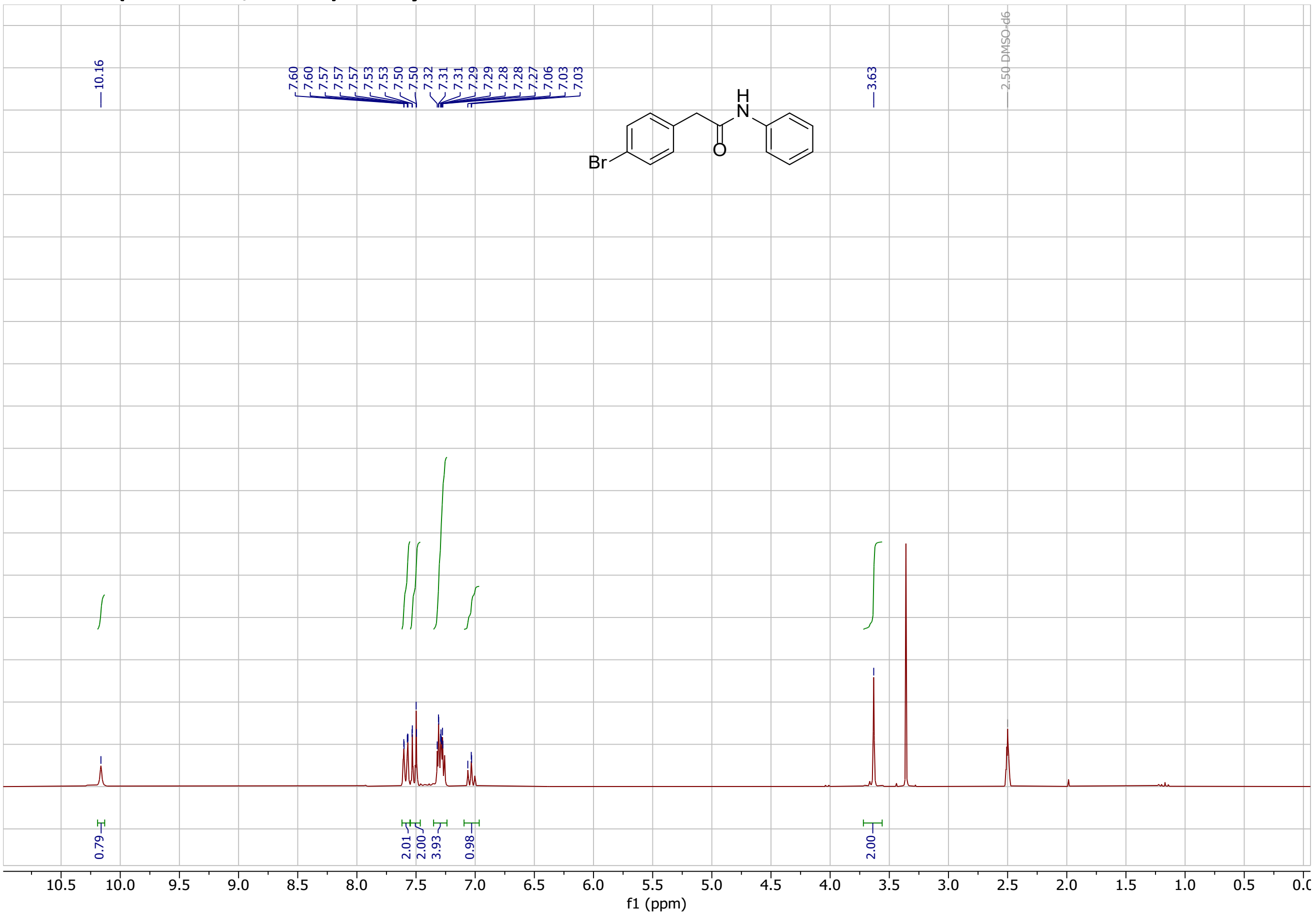

^{1}H NMR (400 MHz, $(CD_3)_2CO$)) Analysis of 3ia.

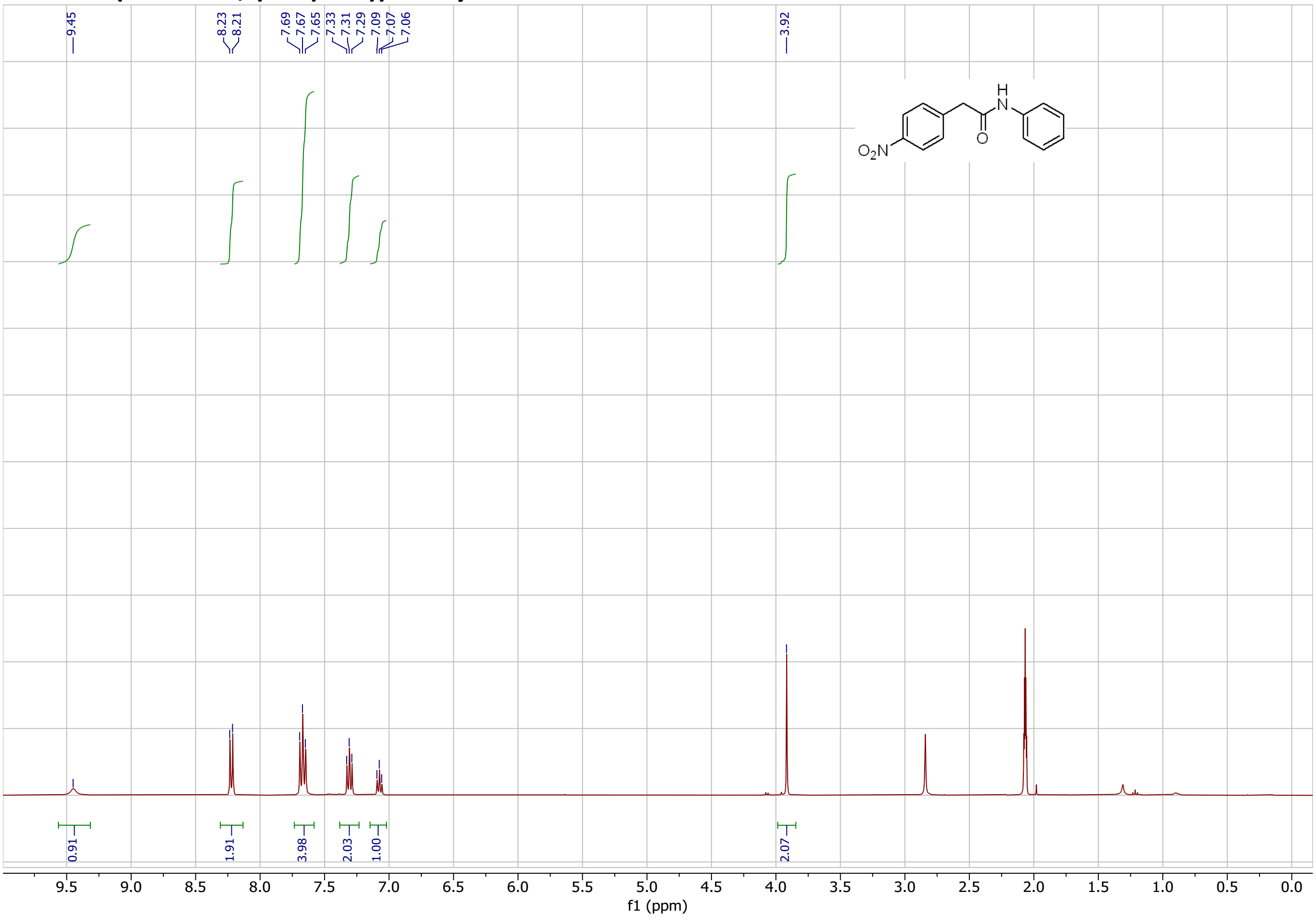

^{13}C NMR (101 MHz, $(CD_3)_2CO$)) Analysis of 3ia.

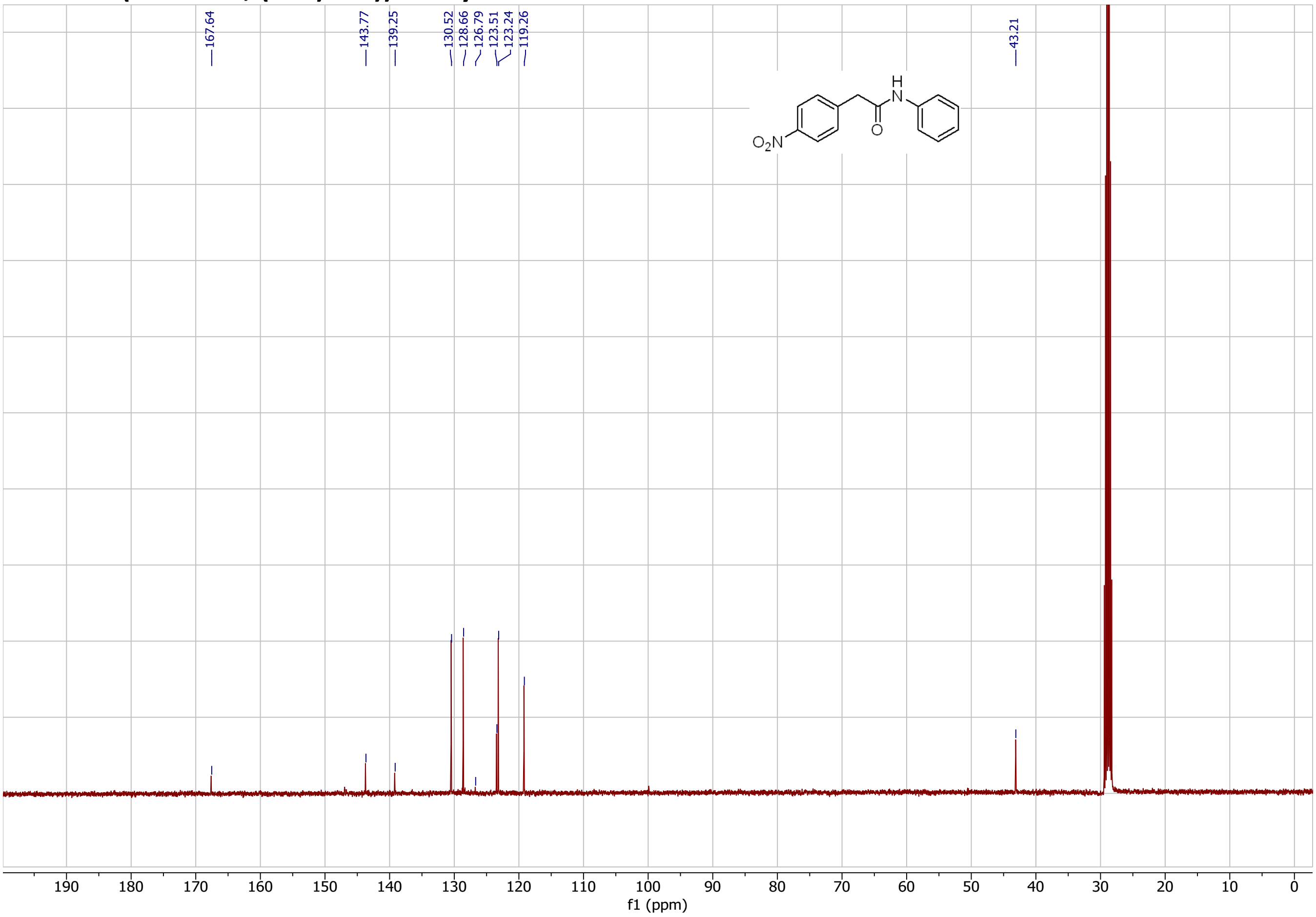

^{1}H NMR (400 MHz, CDCl$_3$) Analysis of 3ja.

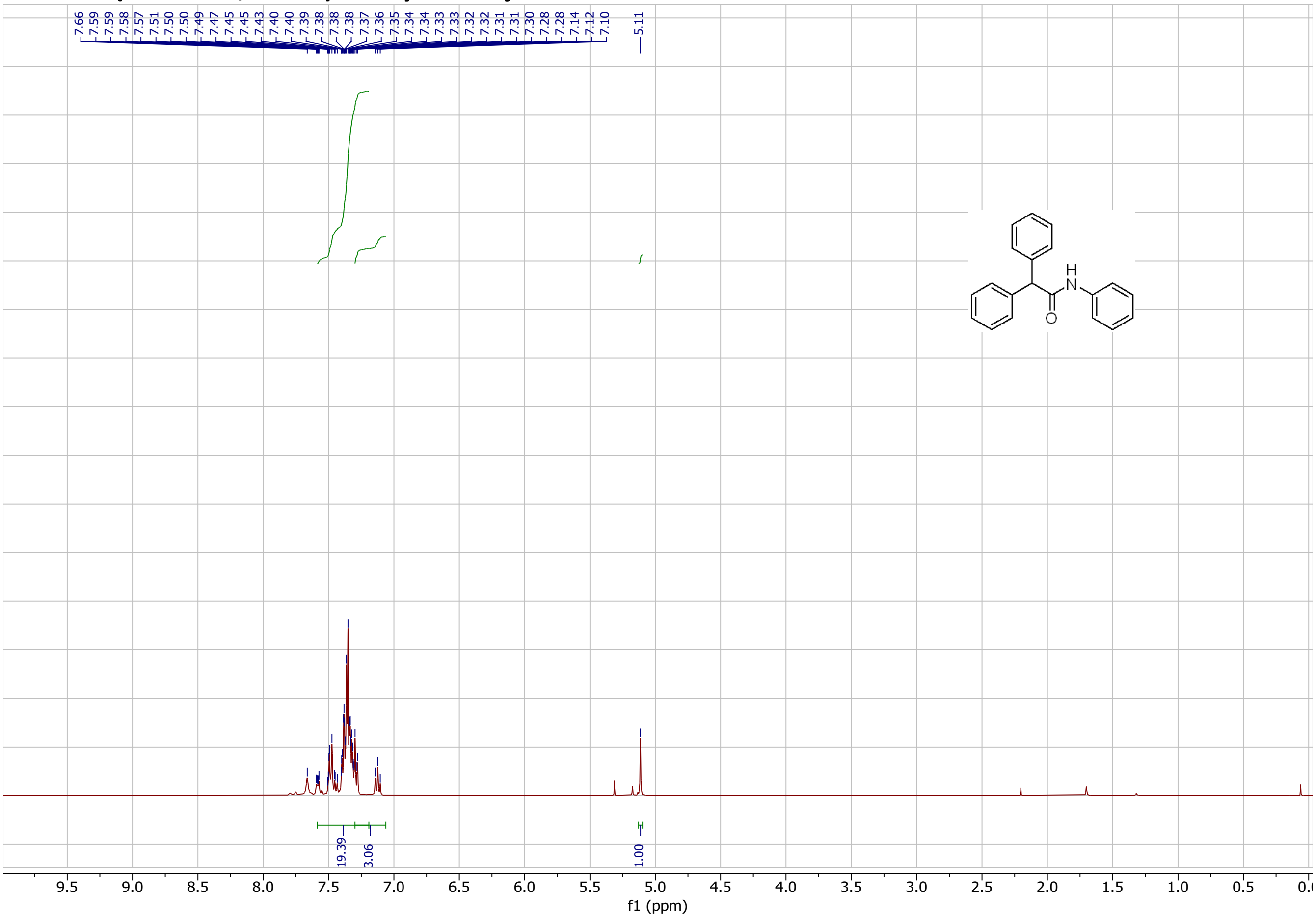

^{13}C NMR (101 MHz, CDCl$_3$) Analysis of 3ja.

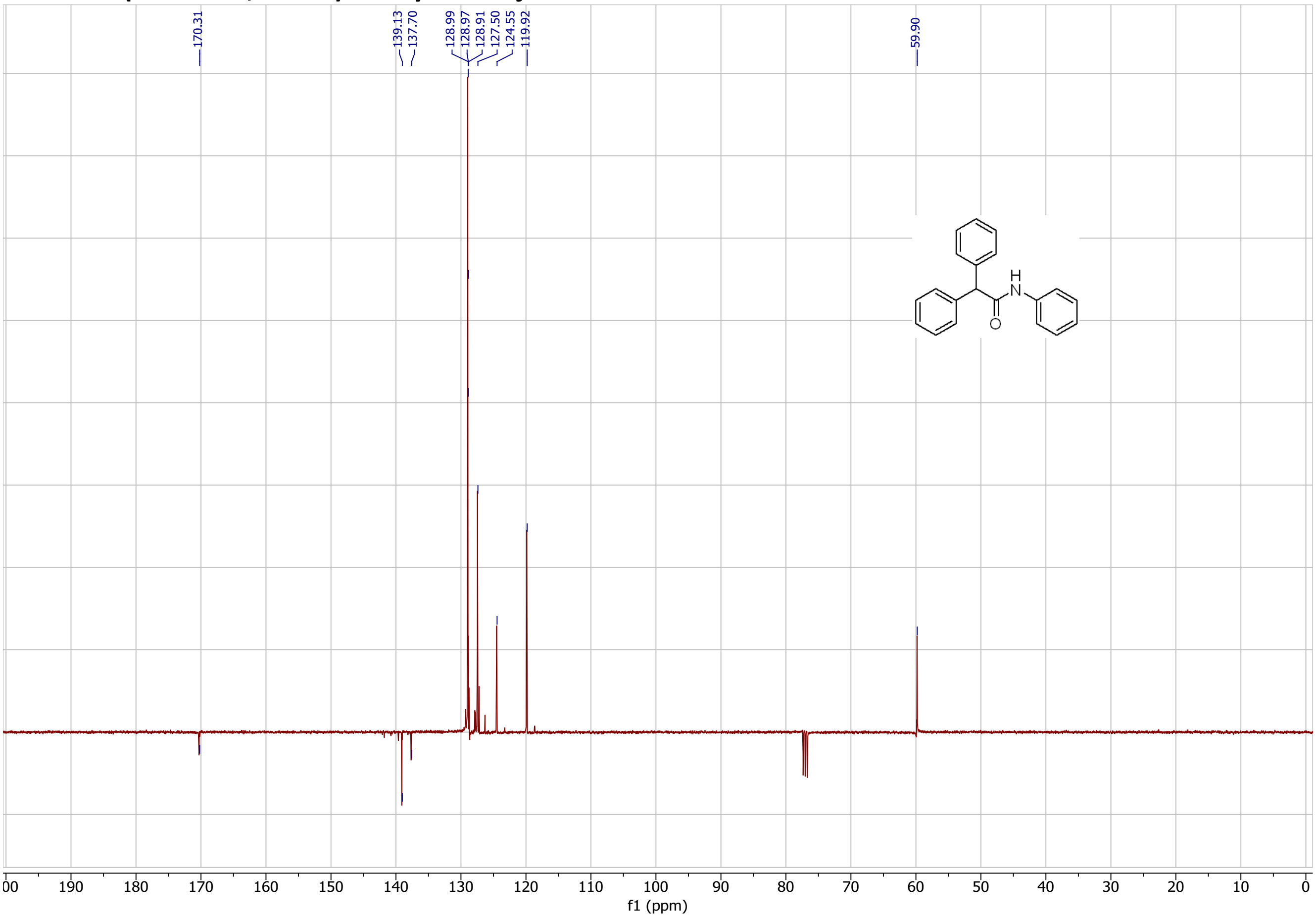

^{1}H NMR (400 MHz, $CDCl_3$) Analysis of 3ab.

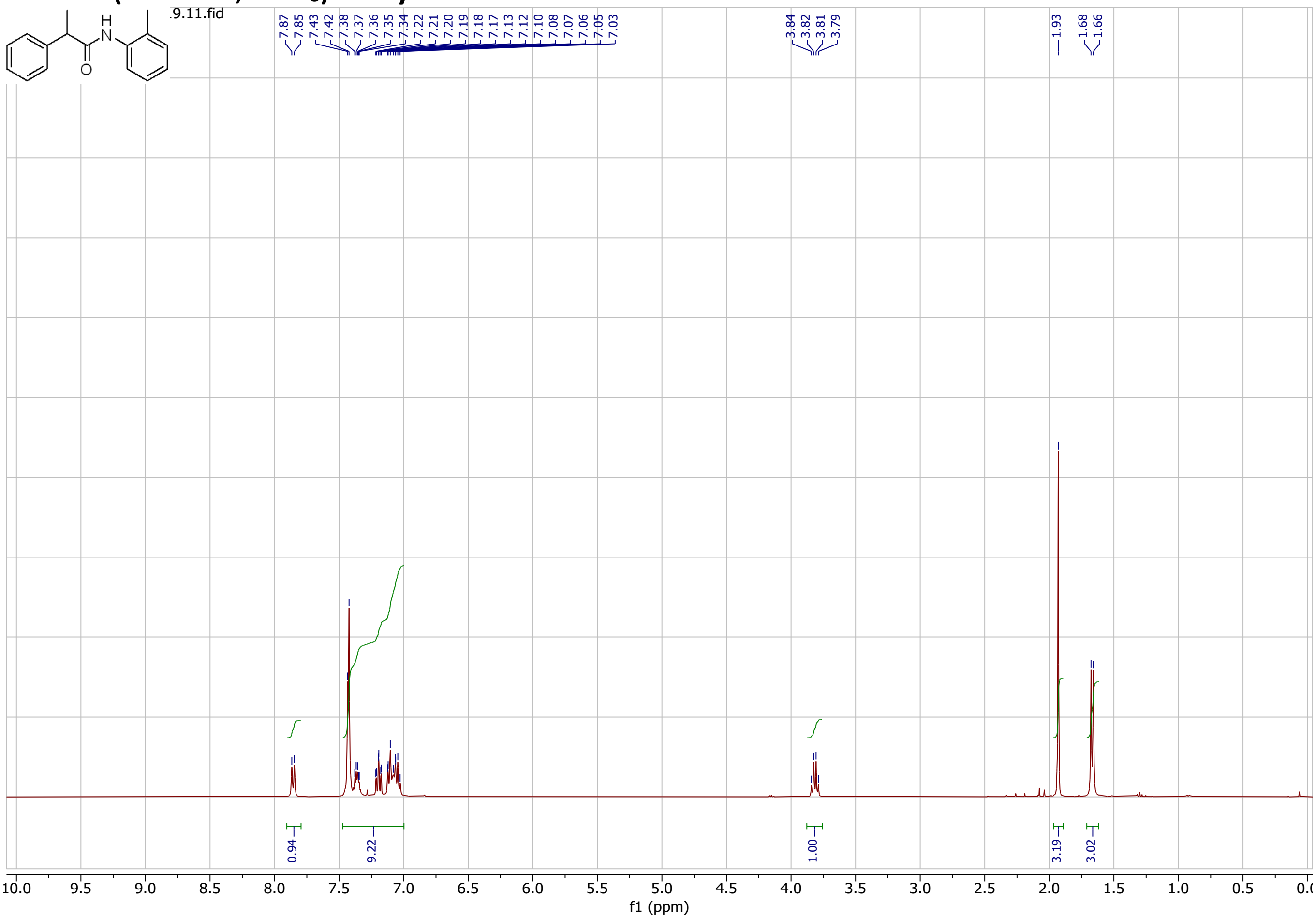

^{13}C NMR (101 MHz, $CDCl_3$) Analysis of 3ab.

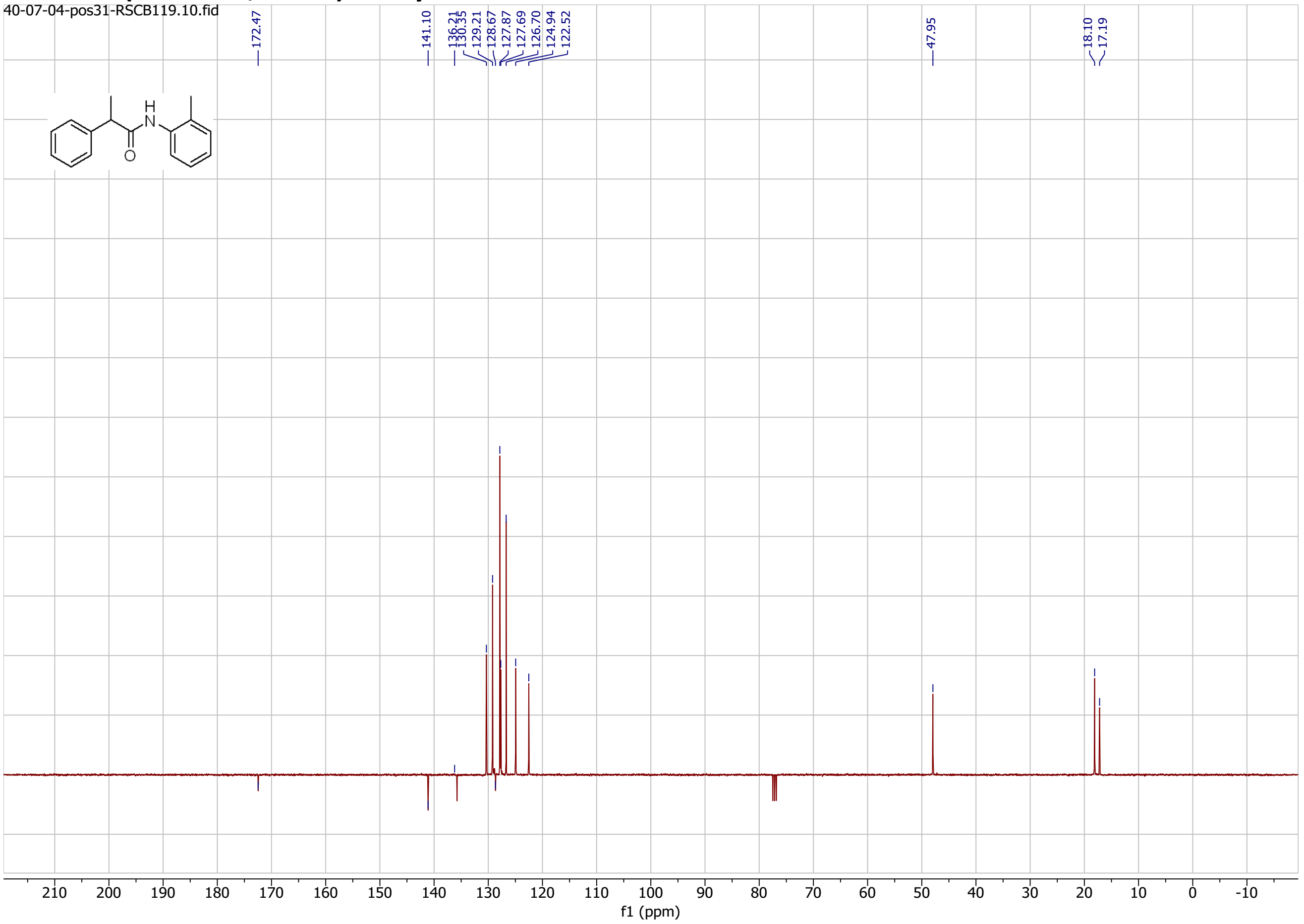

^{1}H NMR (400 MHz, CDCl$_3$) Analysis of 3ac.

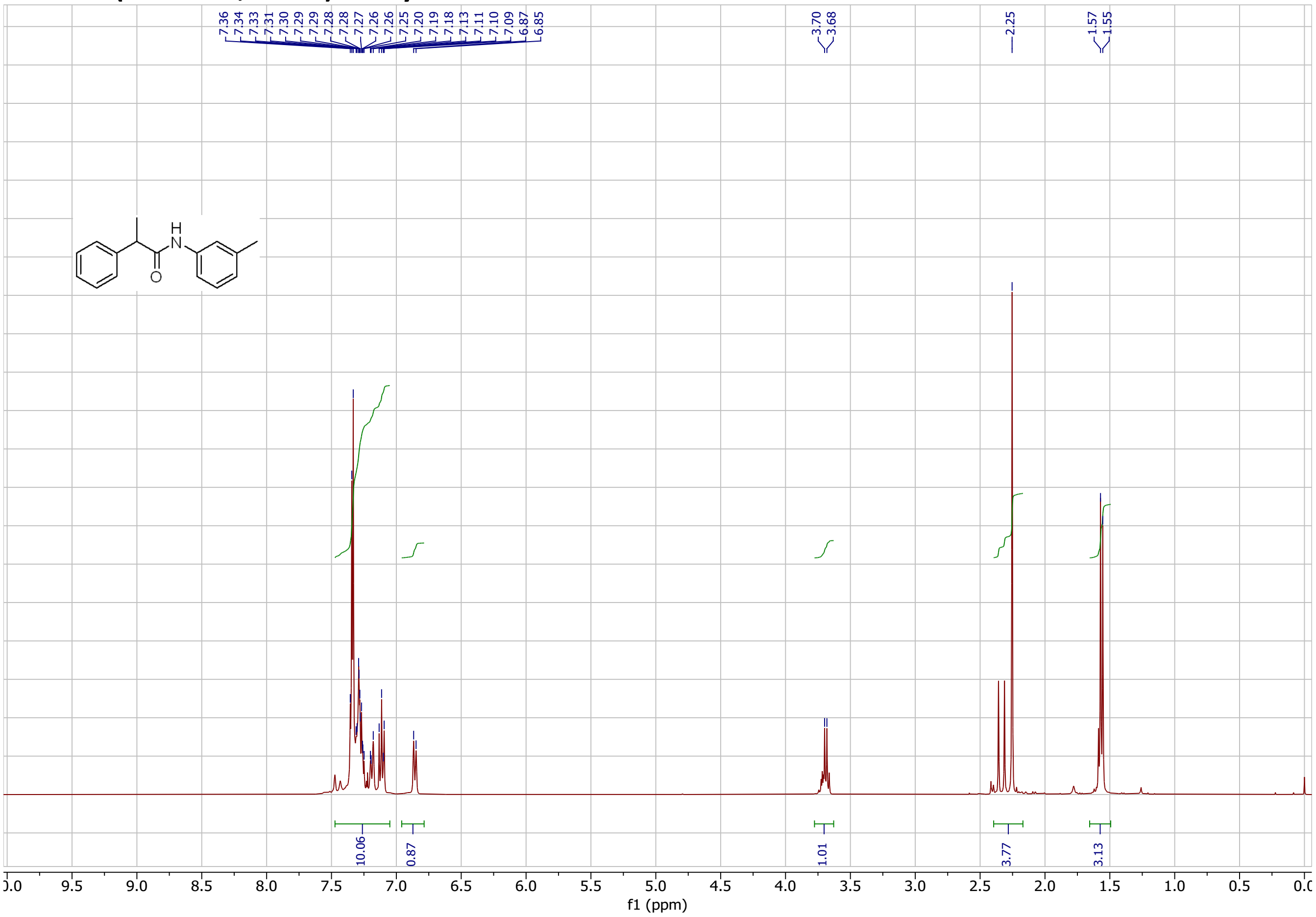

^{13}C NMR (101 MHz, CDCl$_3$) Analysis of 3ac.

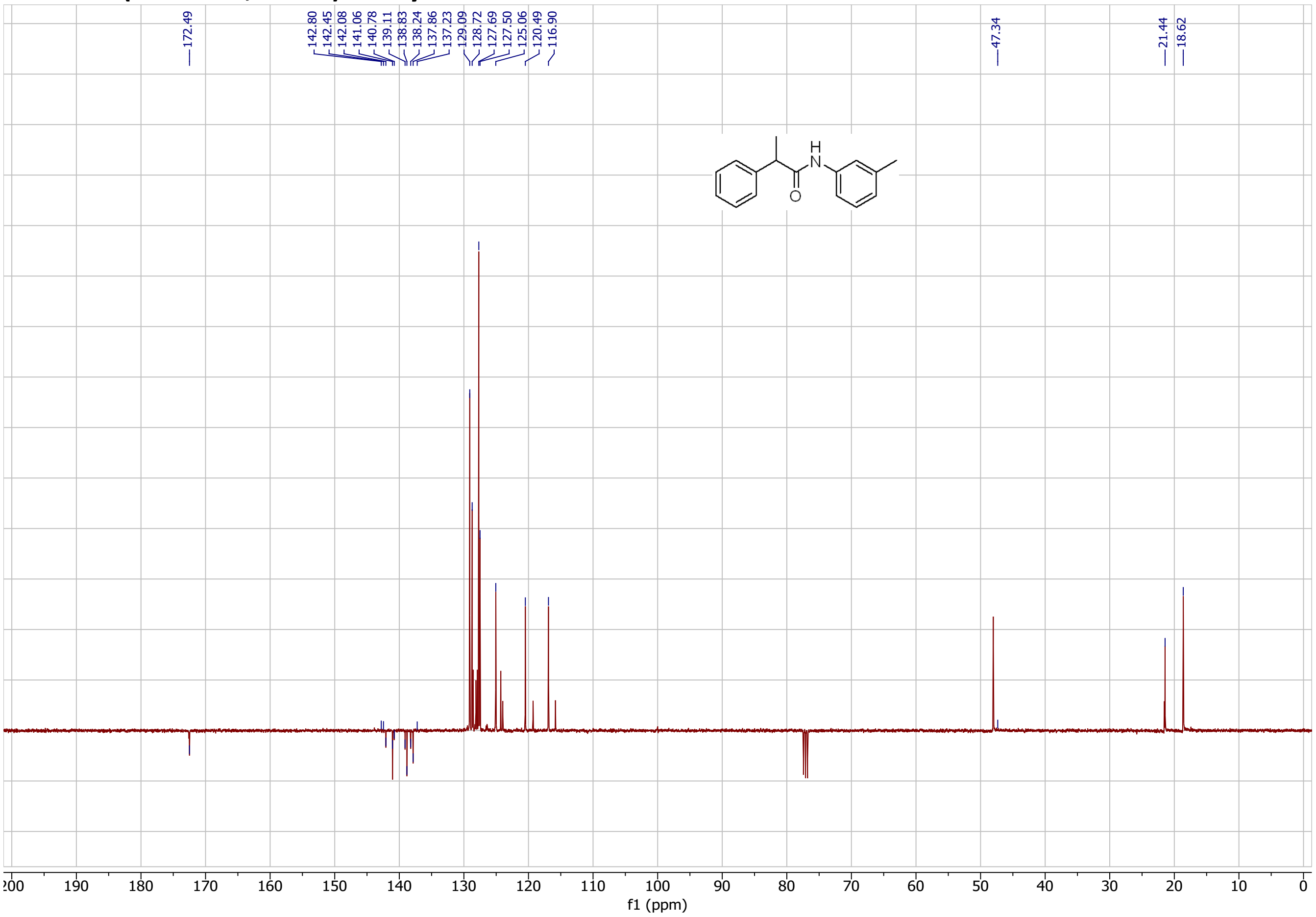

¹H NMR (400 MHz, CDCl$_3$) Analysis of 3ad.

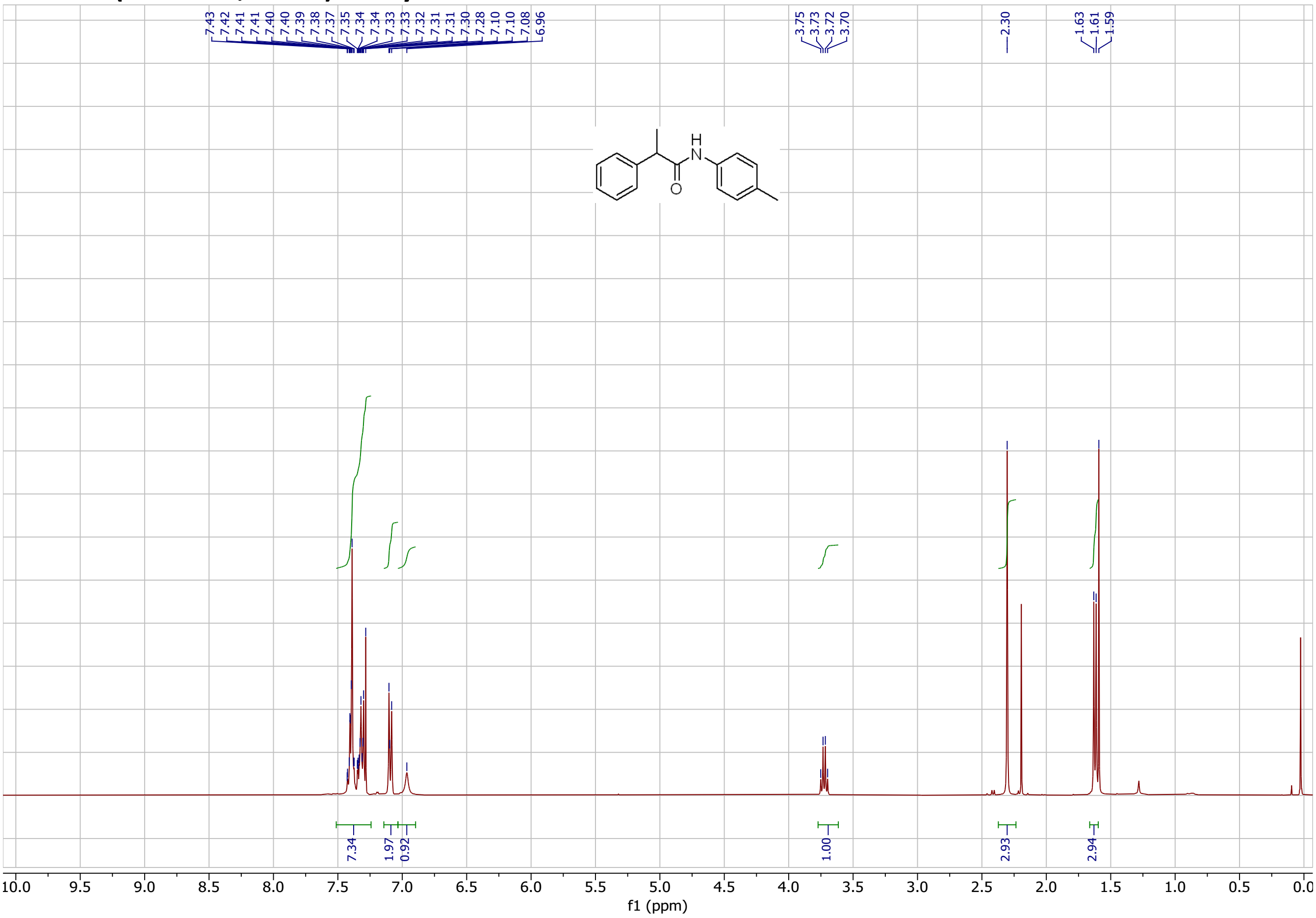

¹³C NMR (101 MHz, CDCl$_3$) Analysis of 3ad.

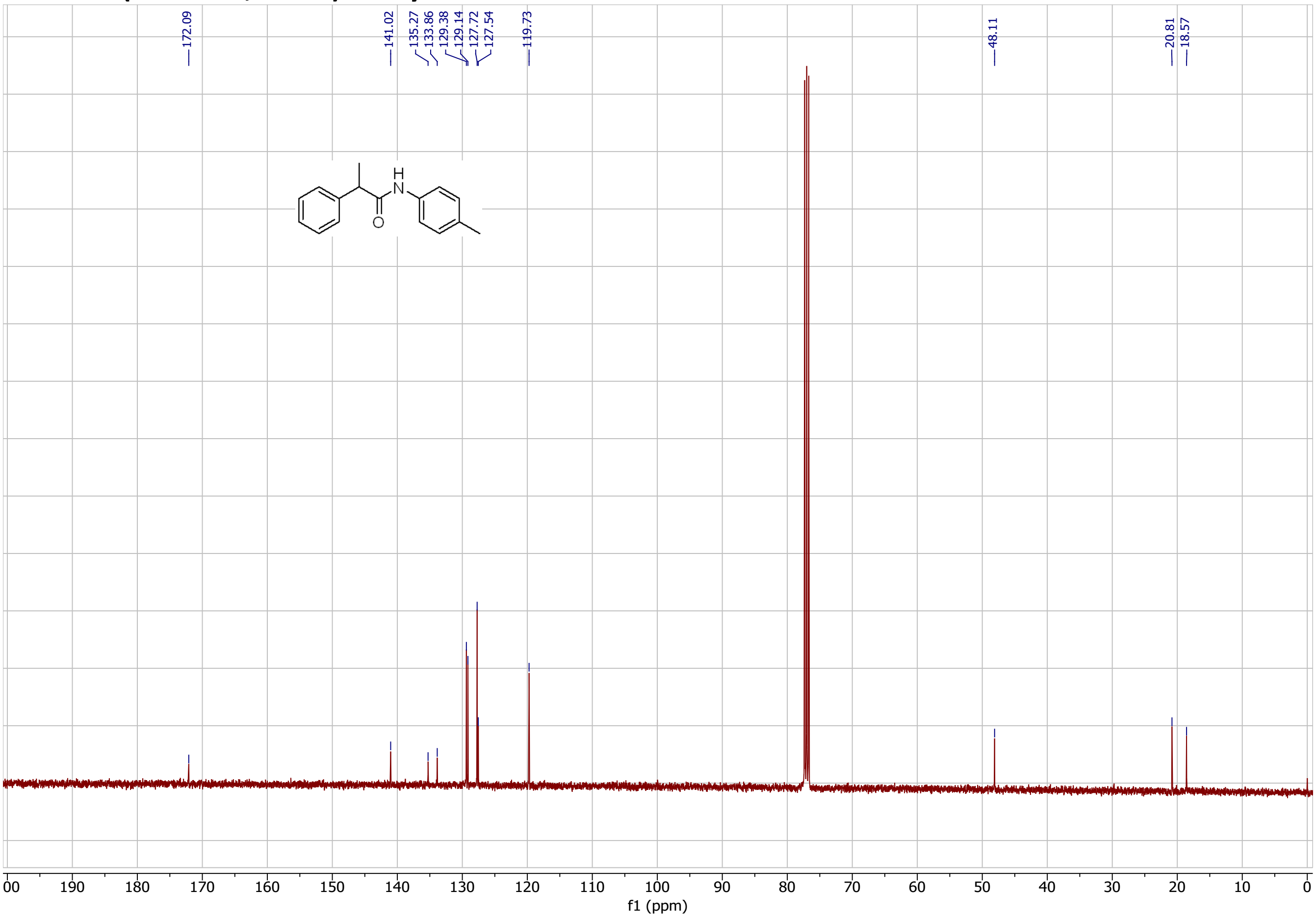

^{1}H NMR (400 MHz, $CDCl_3$) Analysis of 3ae.

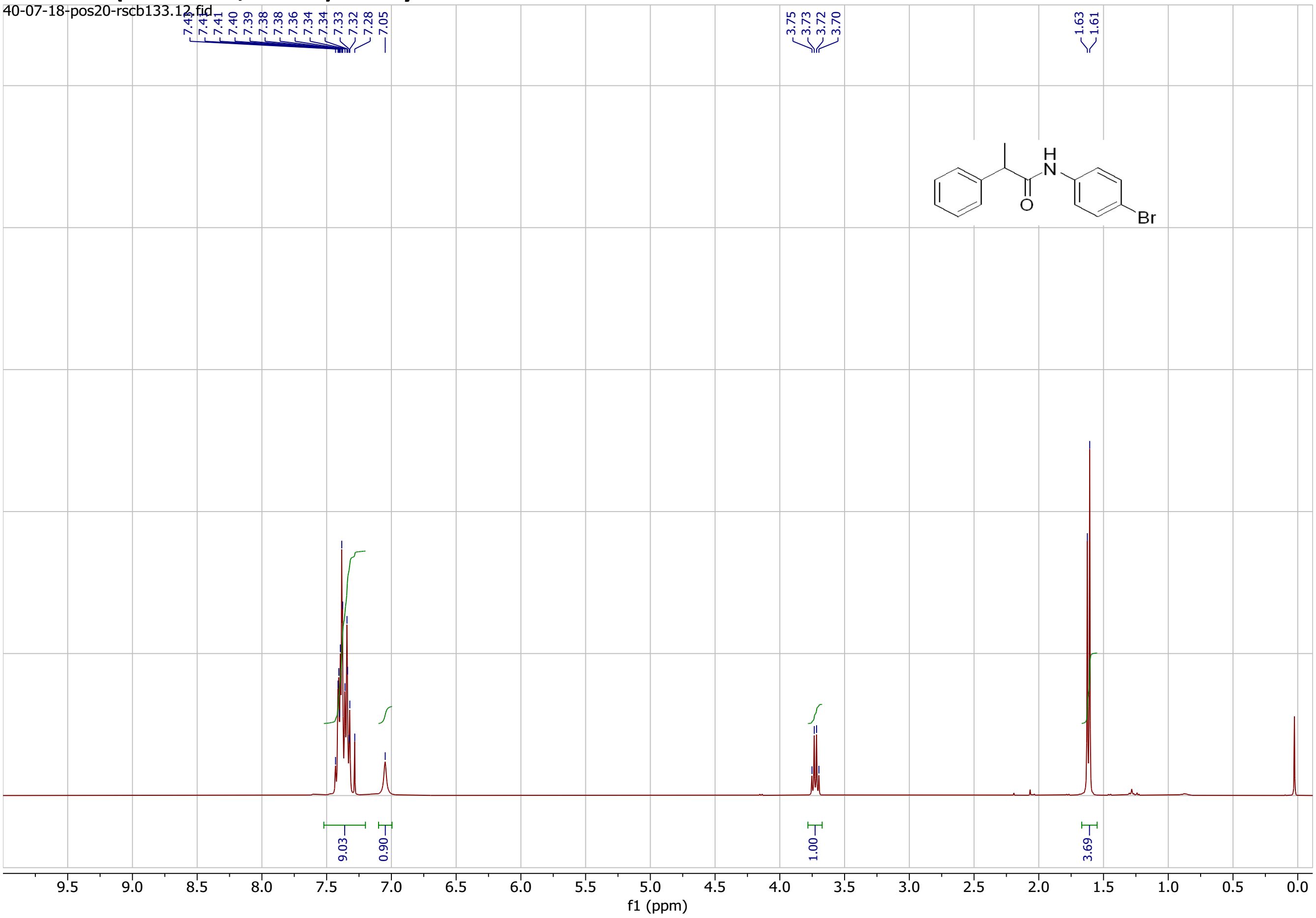

^{13}C NMR (101 MHz, $CDCl_3$) Analysis of 3ae.

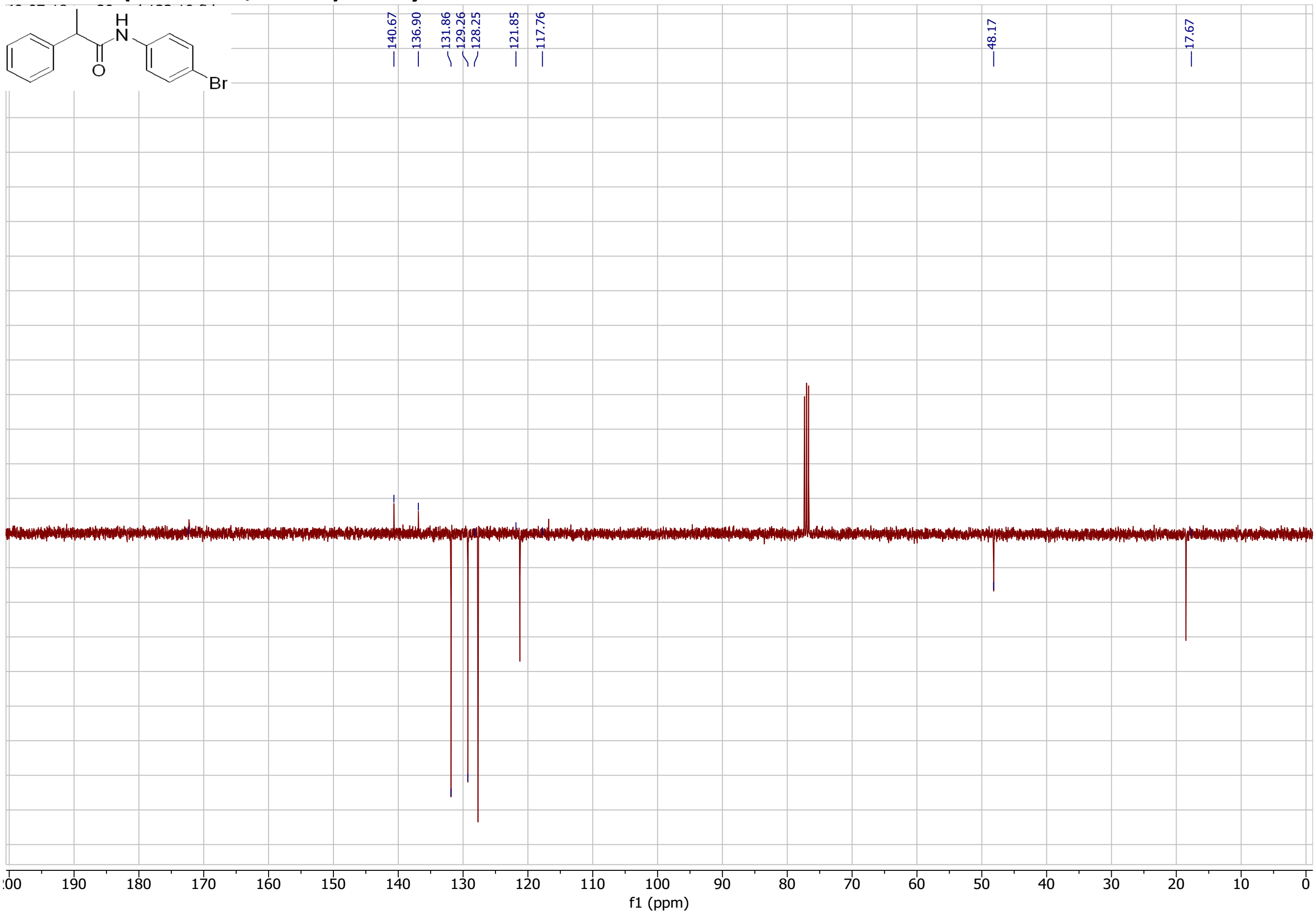

^{1}H NMR (400 MHz, $CDCl_3$) Analysis of 3af.

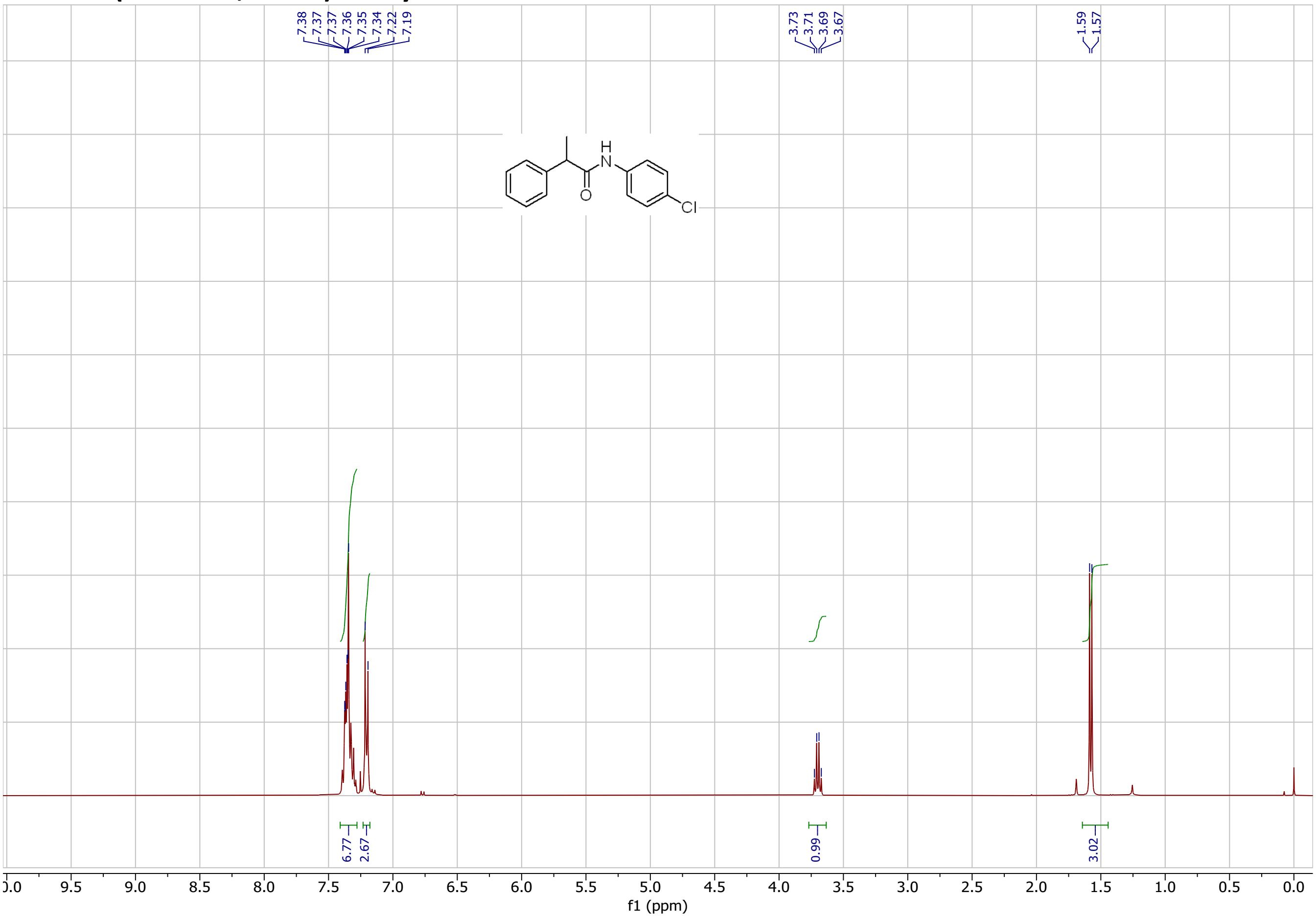

^{13}C NMR (101 MHz, $CDCl_3$) Analysis of 3af.

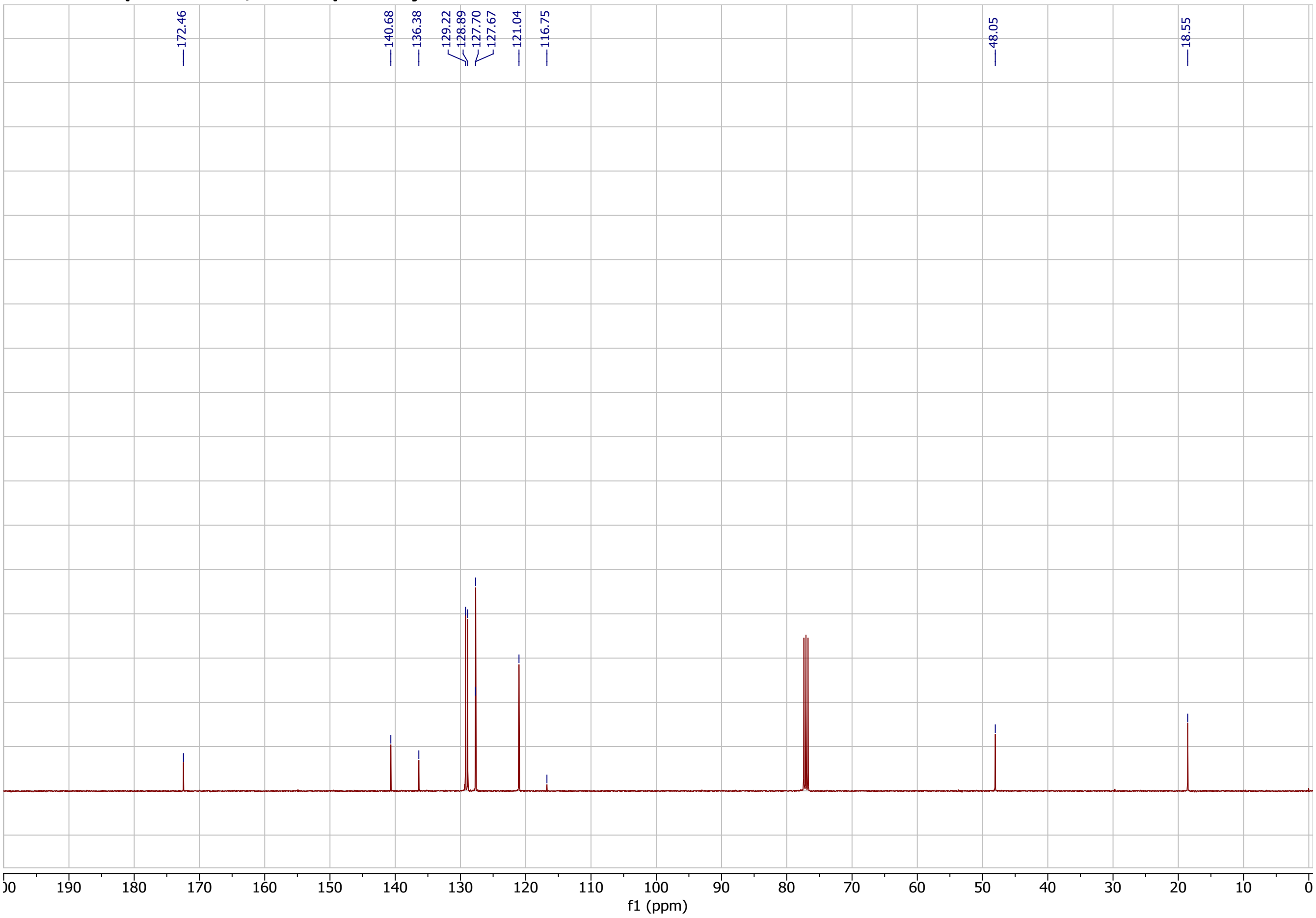

^{1}H NMR (400 MHz, $CDCl_3$) Analysis of 3ag.

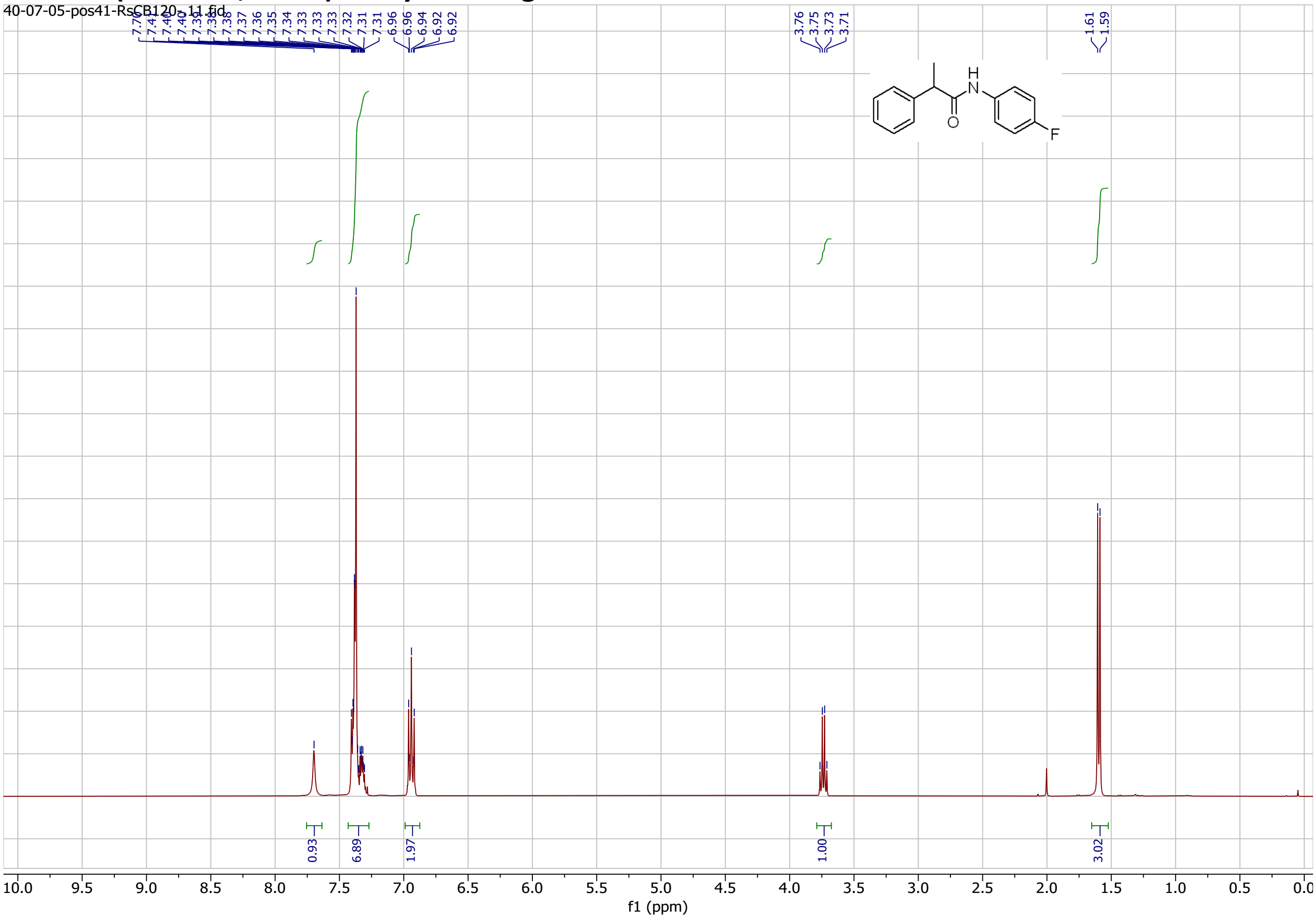

^{13}C NMR (101 MHz, $CDCl_3$) Analysis of 3ag.

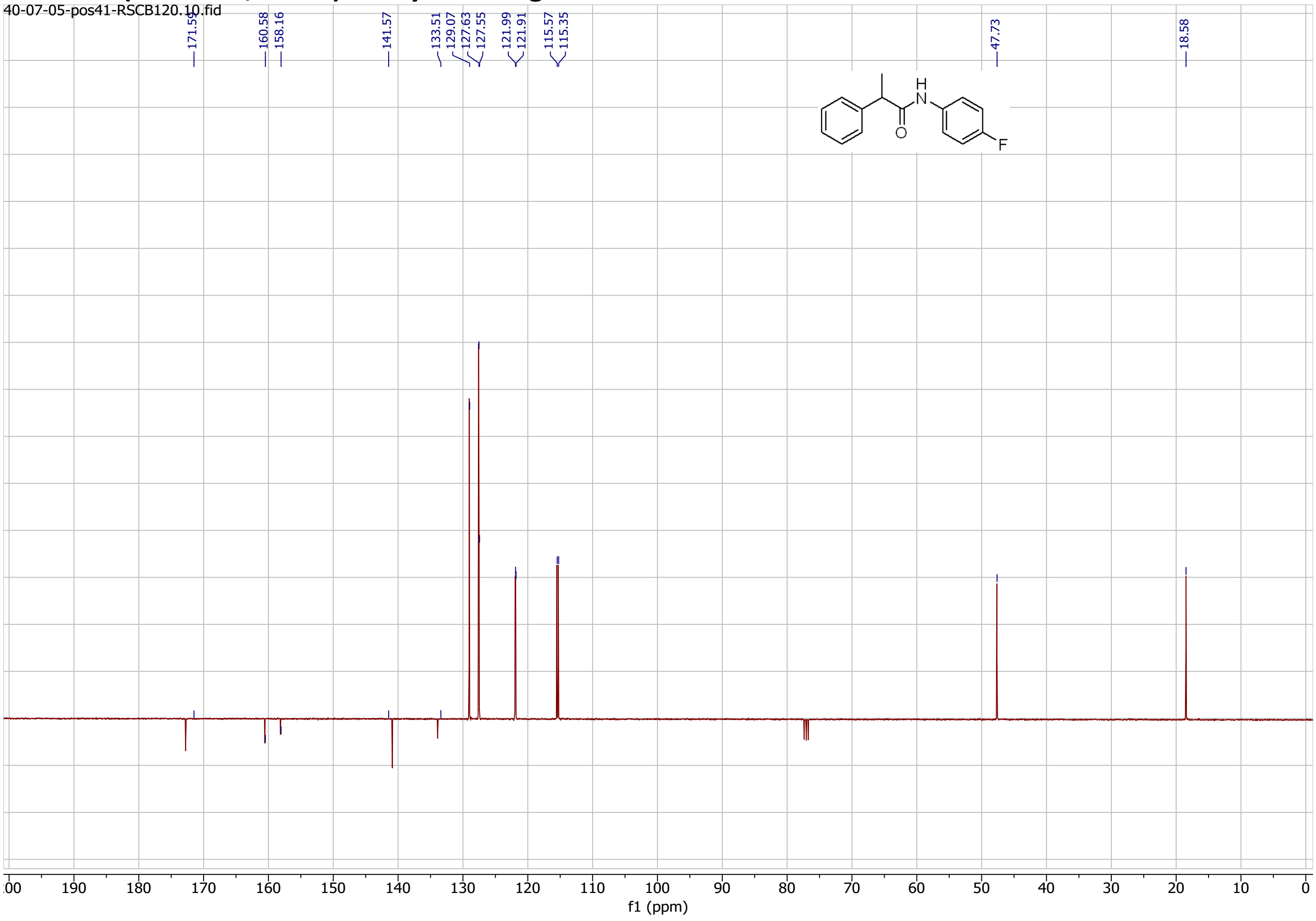

^{1}H NMR (400 MHz, $CDCl_3$) Analysis of 3ah.

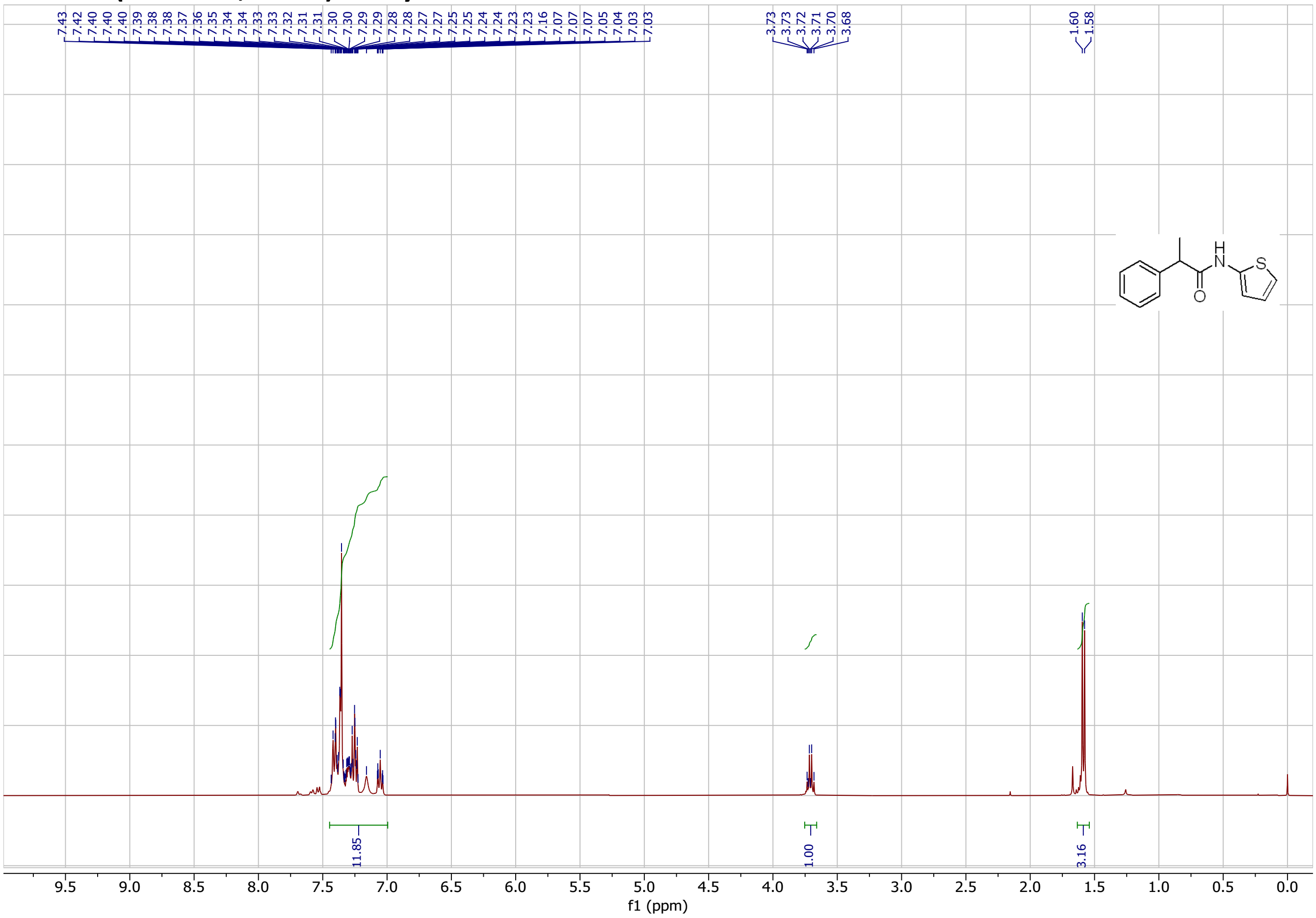

^{13}C NMR (101 MHz, $CDCl_3$) Analysis of 3ah

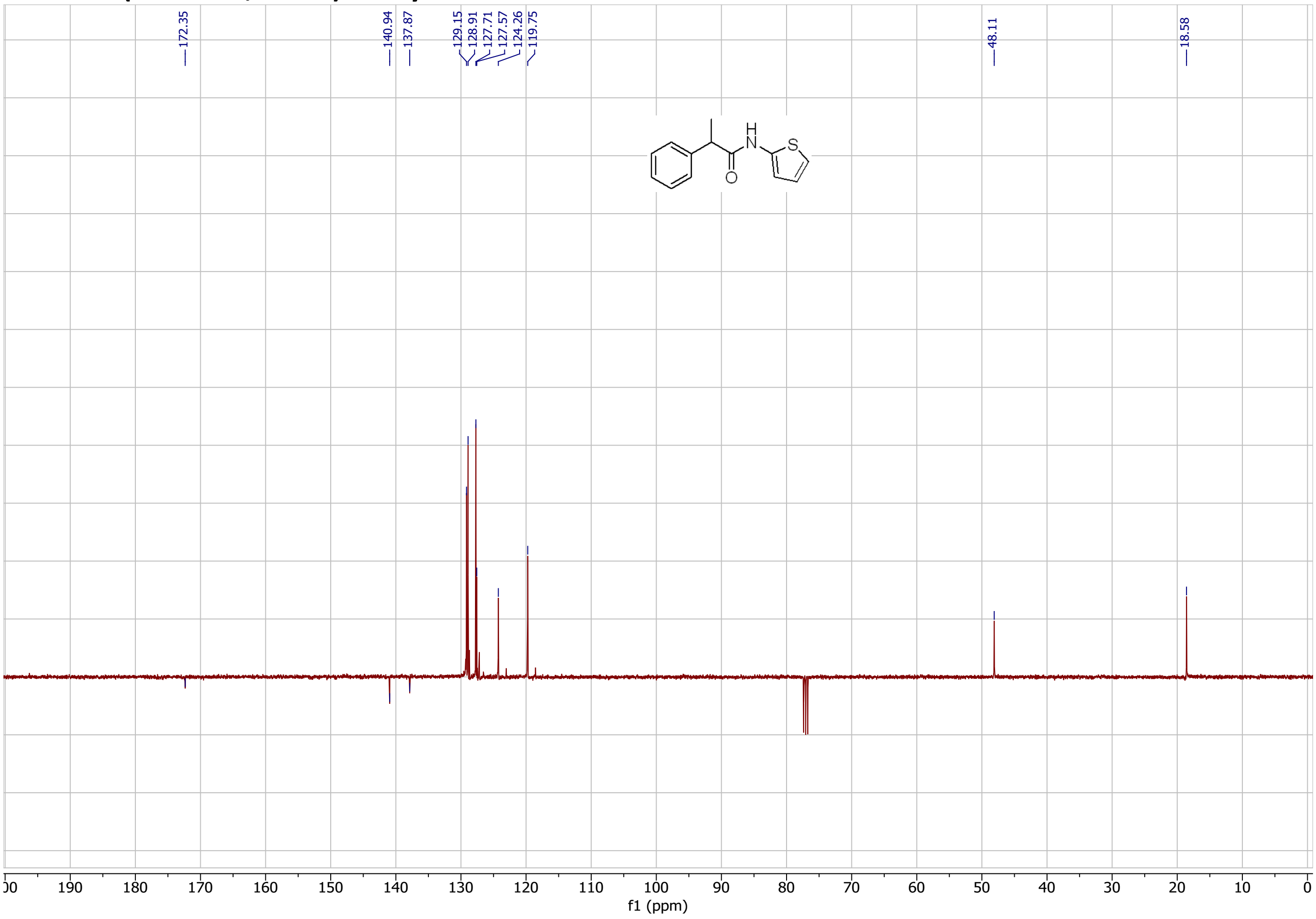

^{1}H NMR (400 MHz) Analysis of 4a.

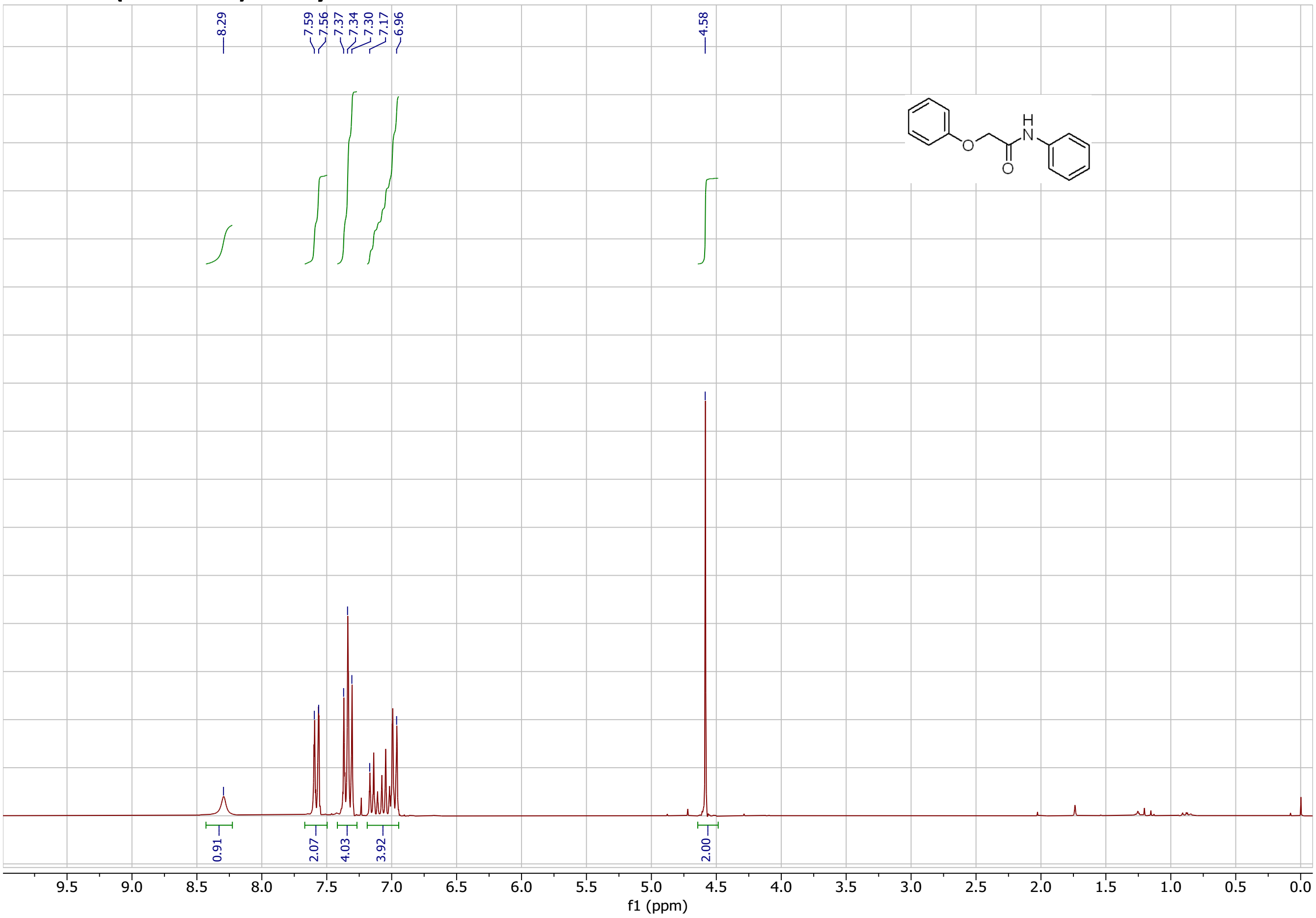

^{13}C NMR (101 MHz) Analysis of 4a.

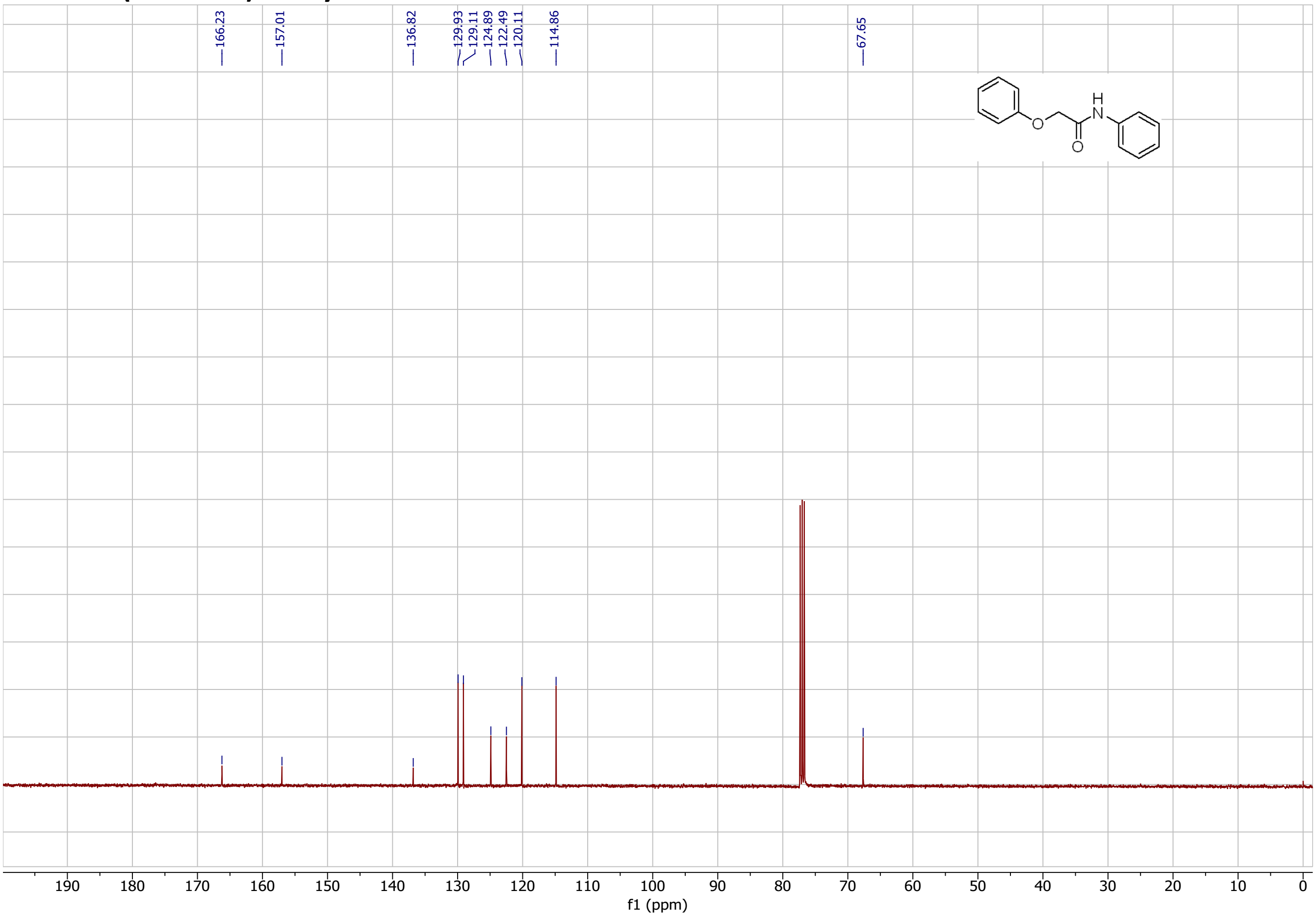

^{1}H NMR (400 MHz) Analysis of 4b.

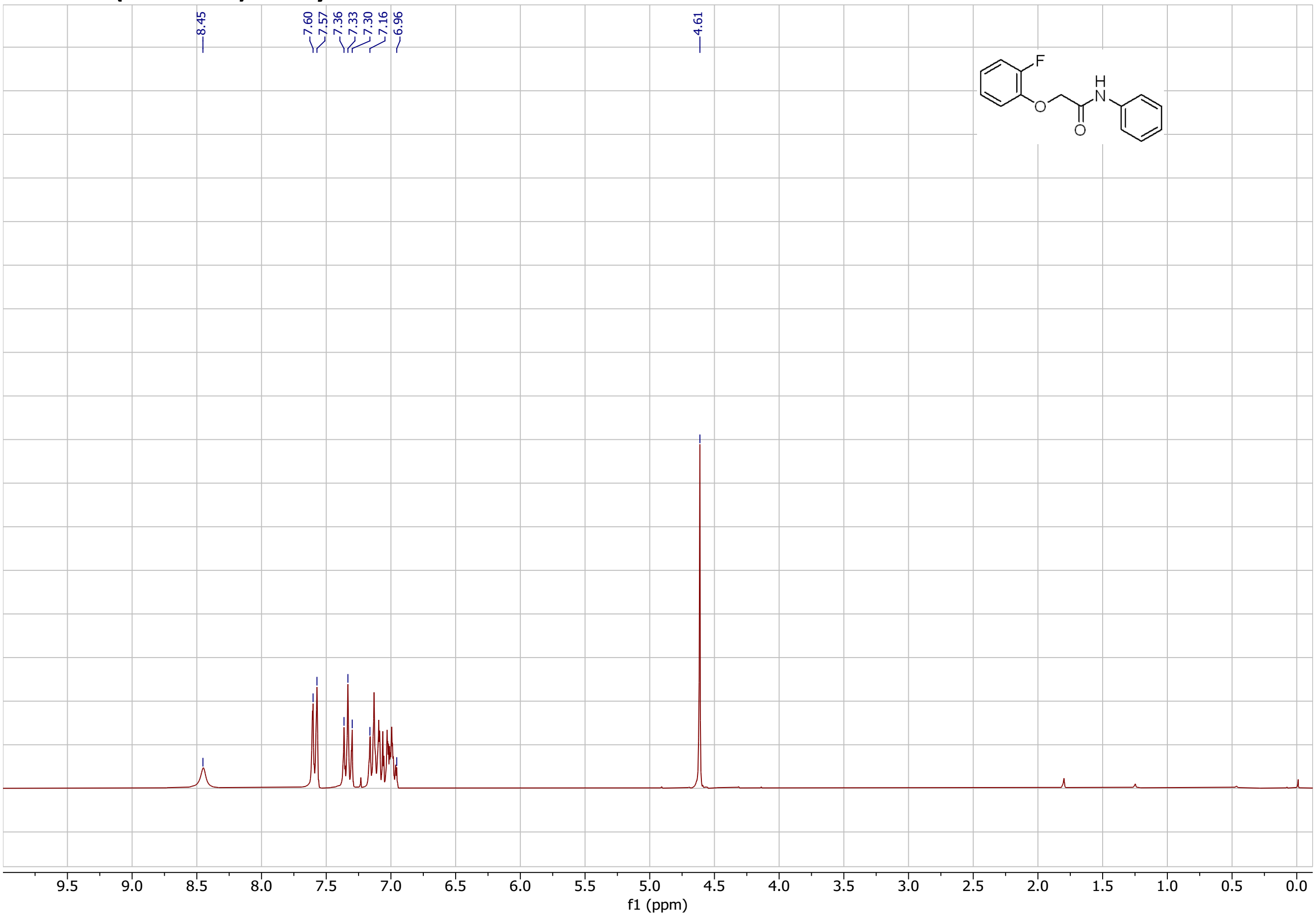

^{13}C NMR (101 MHz) Analysis of 4b.

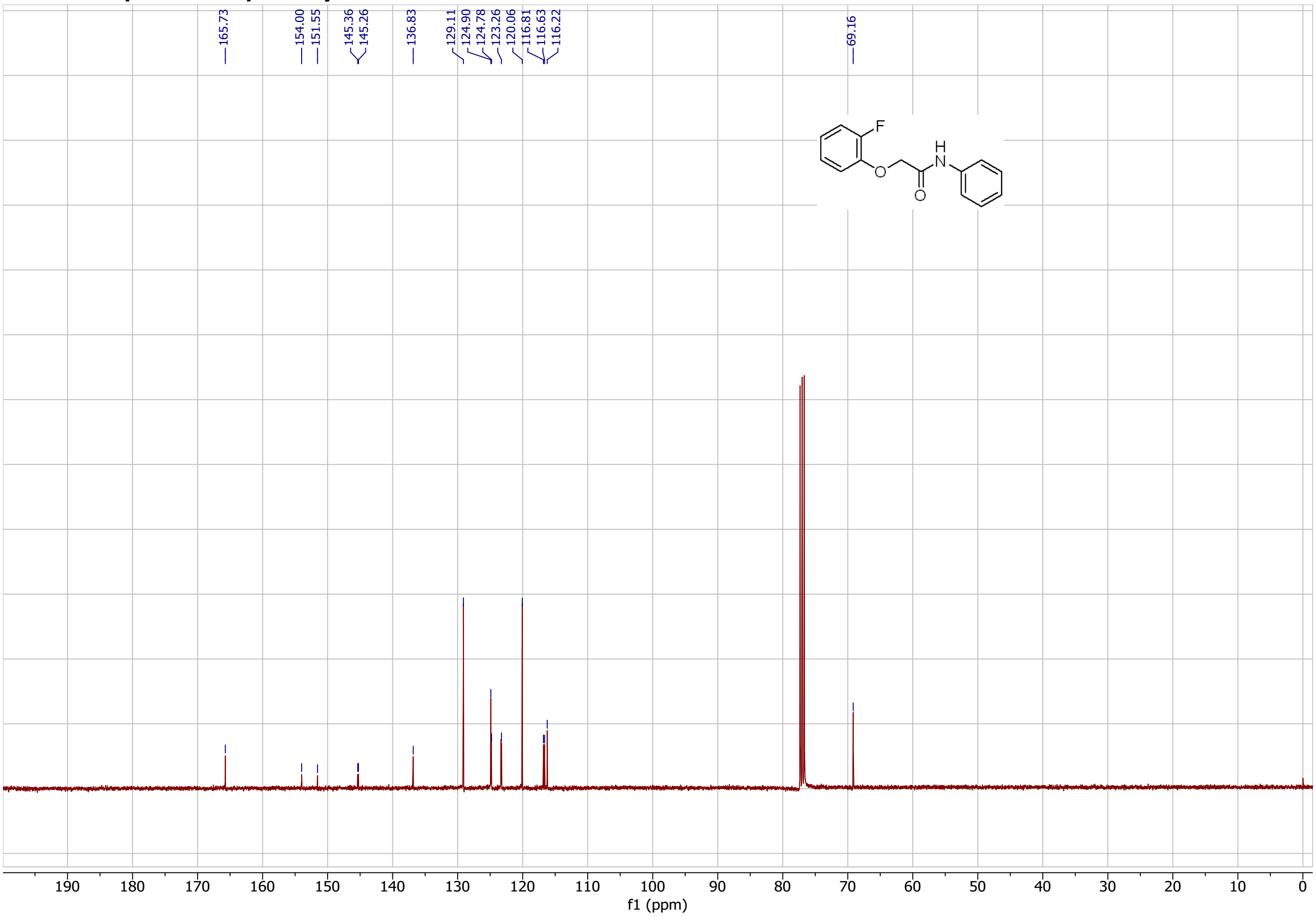

^{1}H NMR (400 MHz) Analysis of 4c.

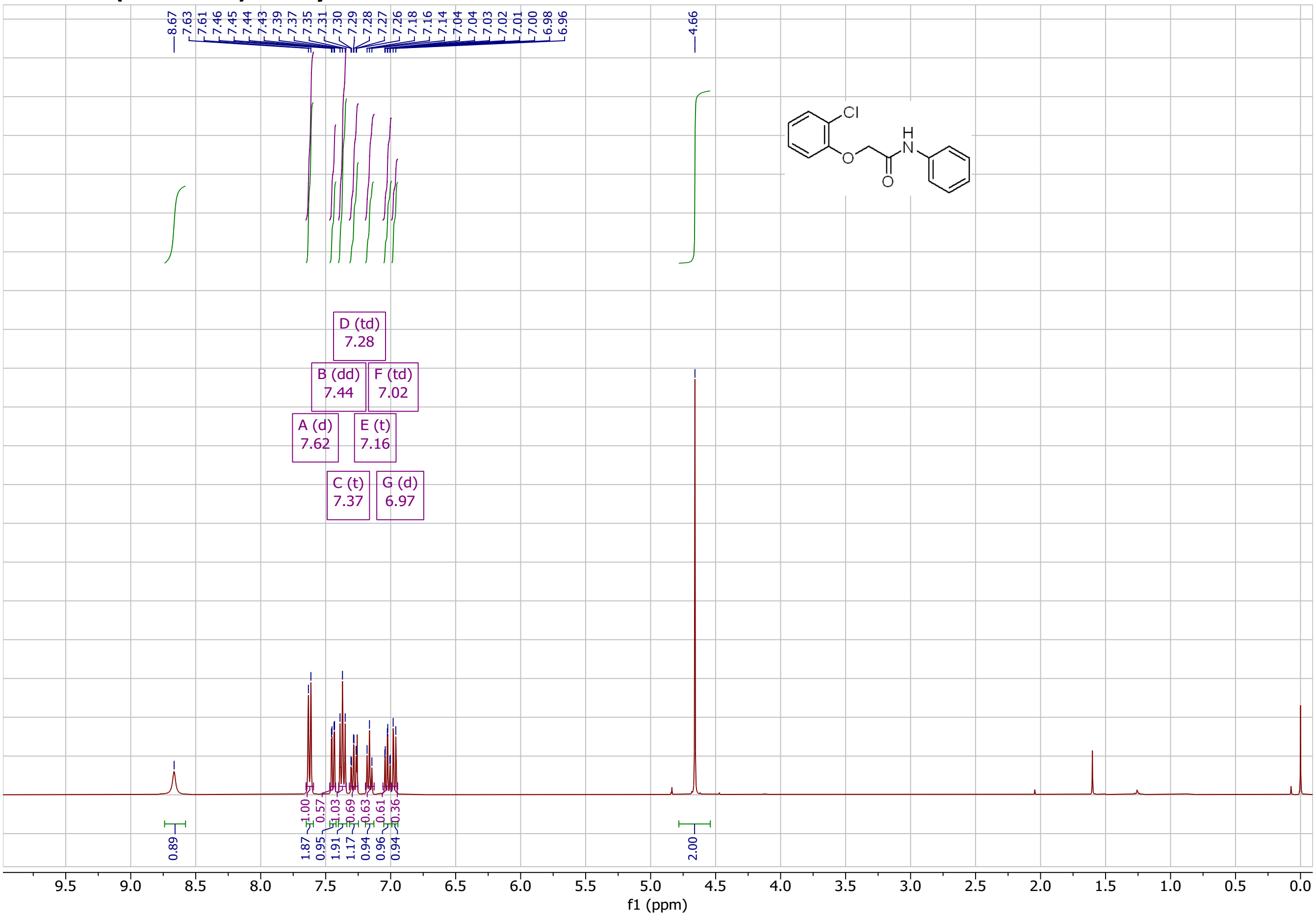

^{13}C NMR (101 MHz) Analysis of 4c.

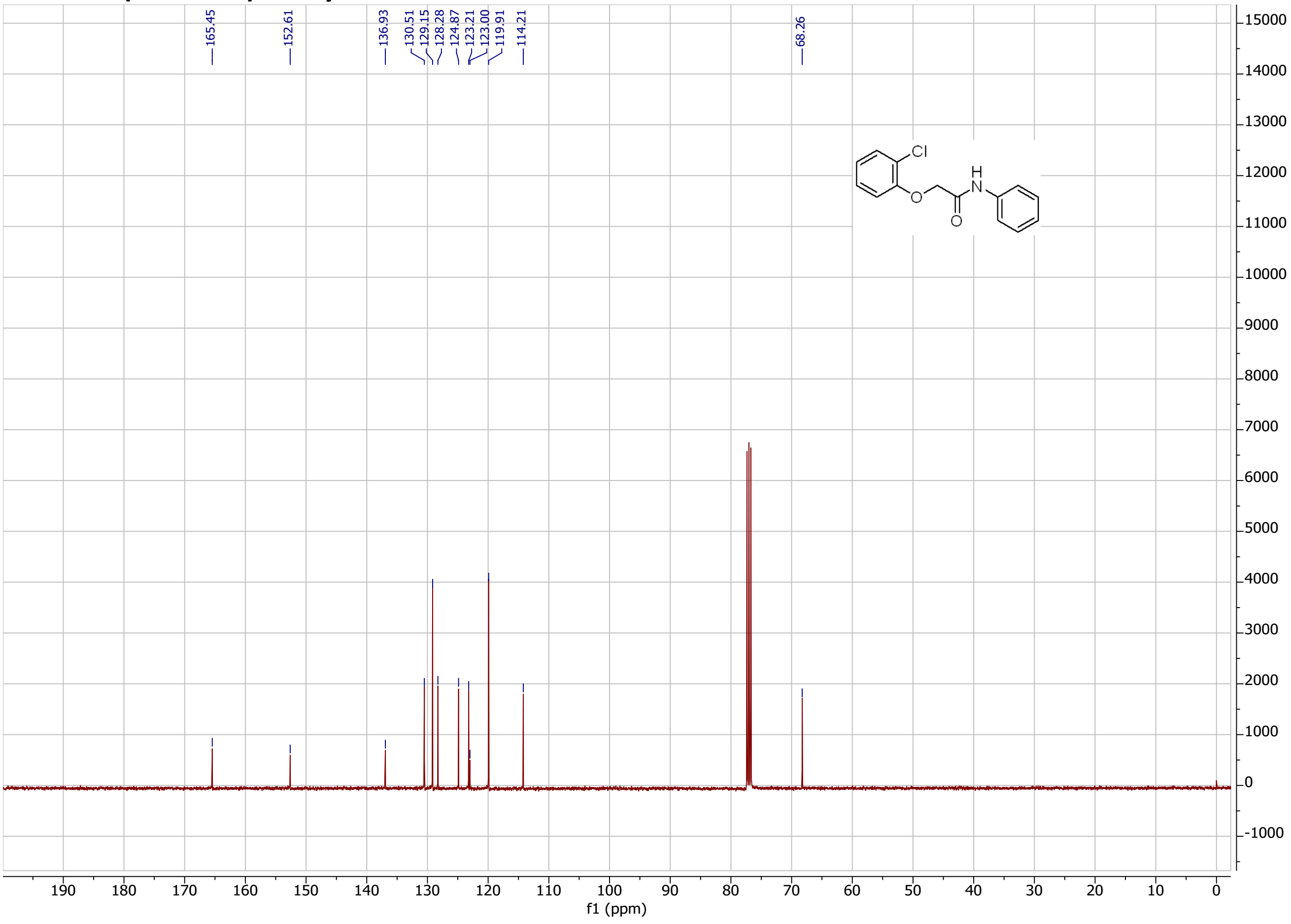

^{1}H NMR (400 MHz) Analysis of 4d.

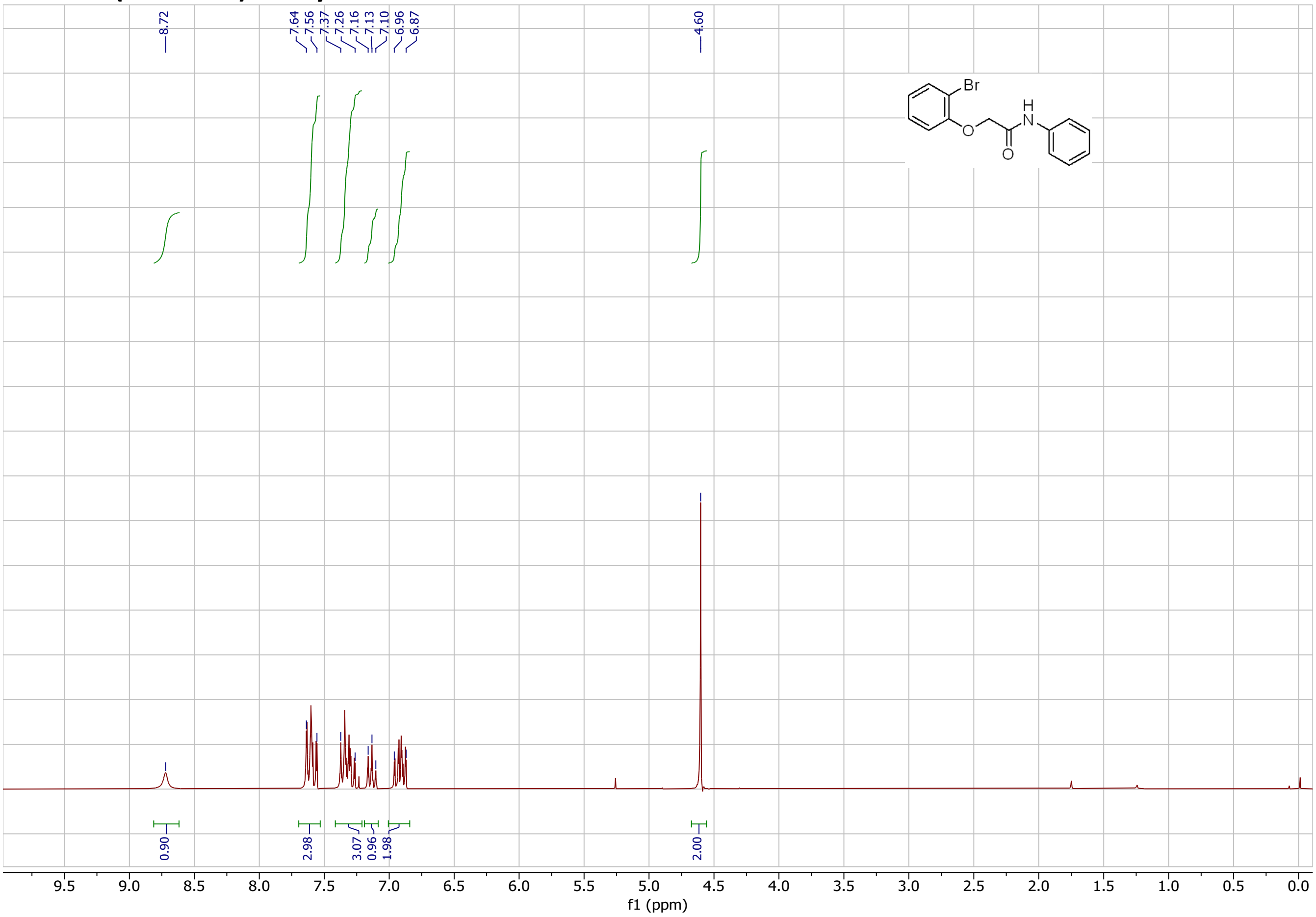

^{13}C NMR (101 MHz) Analysis of 4d.

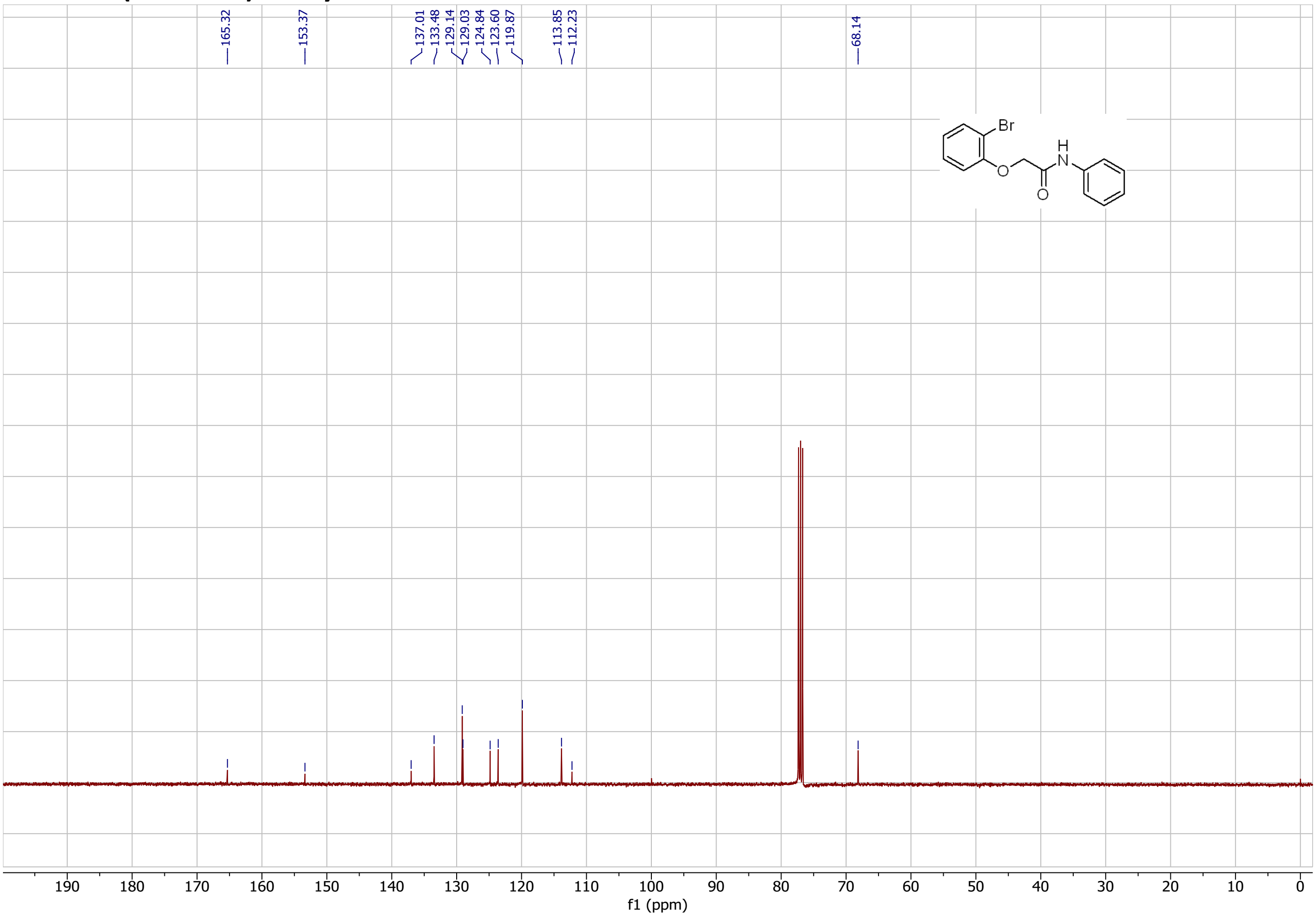

^{1}H NMR (400 MHz) Analysis of 4e.

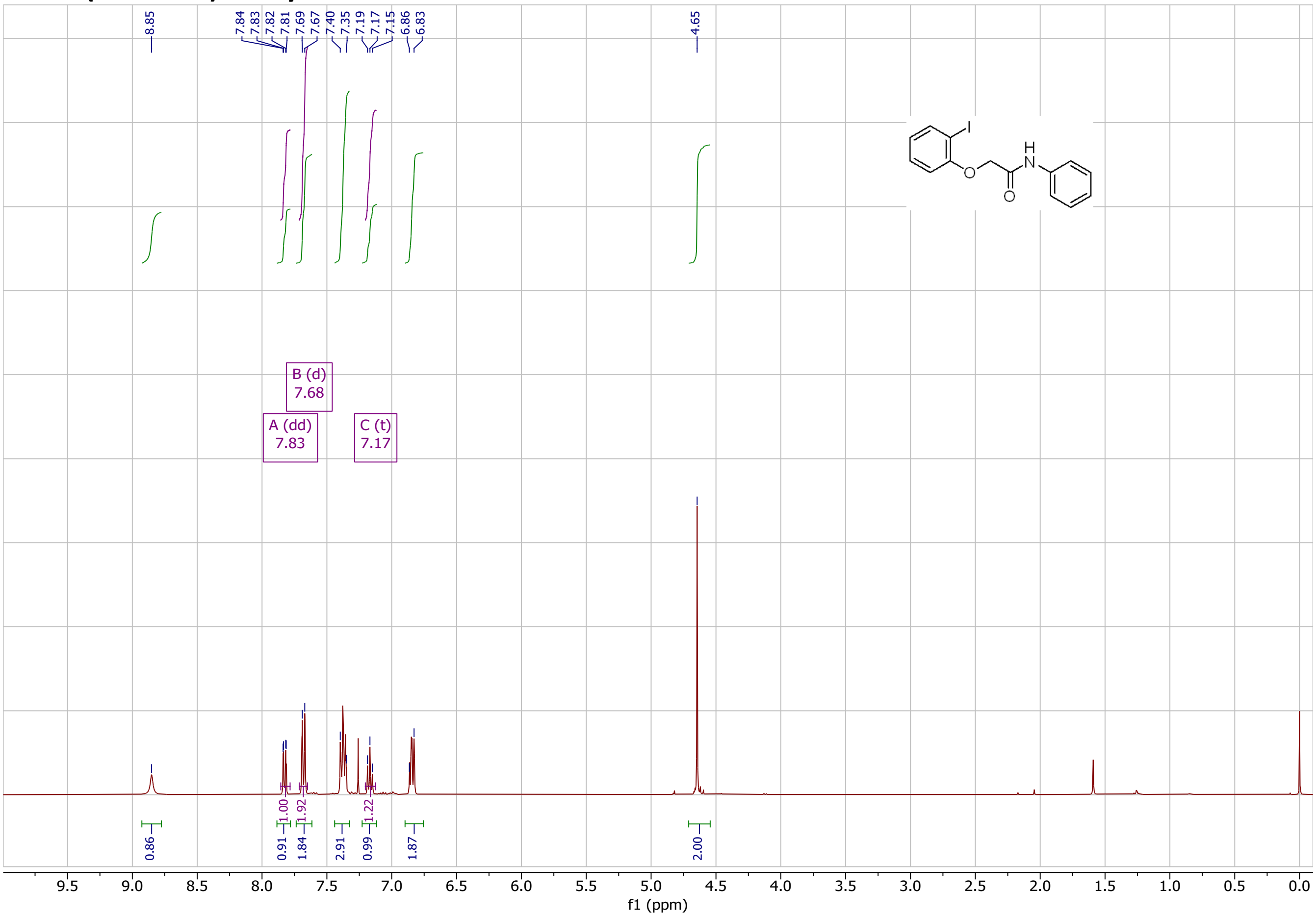

^{13}C NMR (101 MHz) Analysis of 4e.

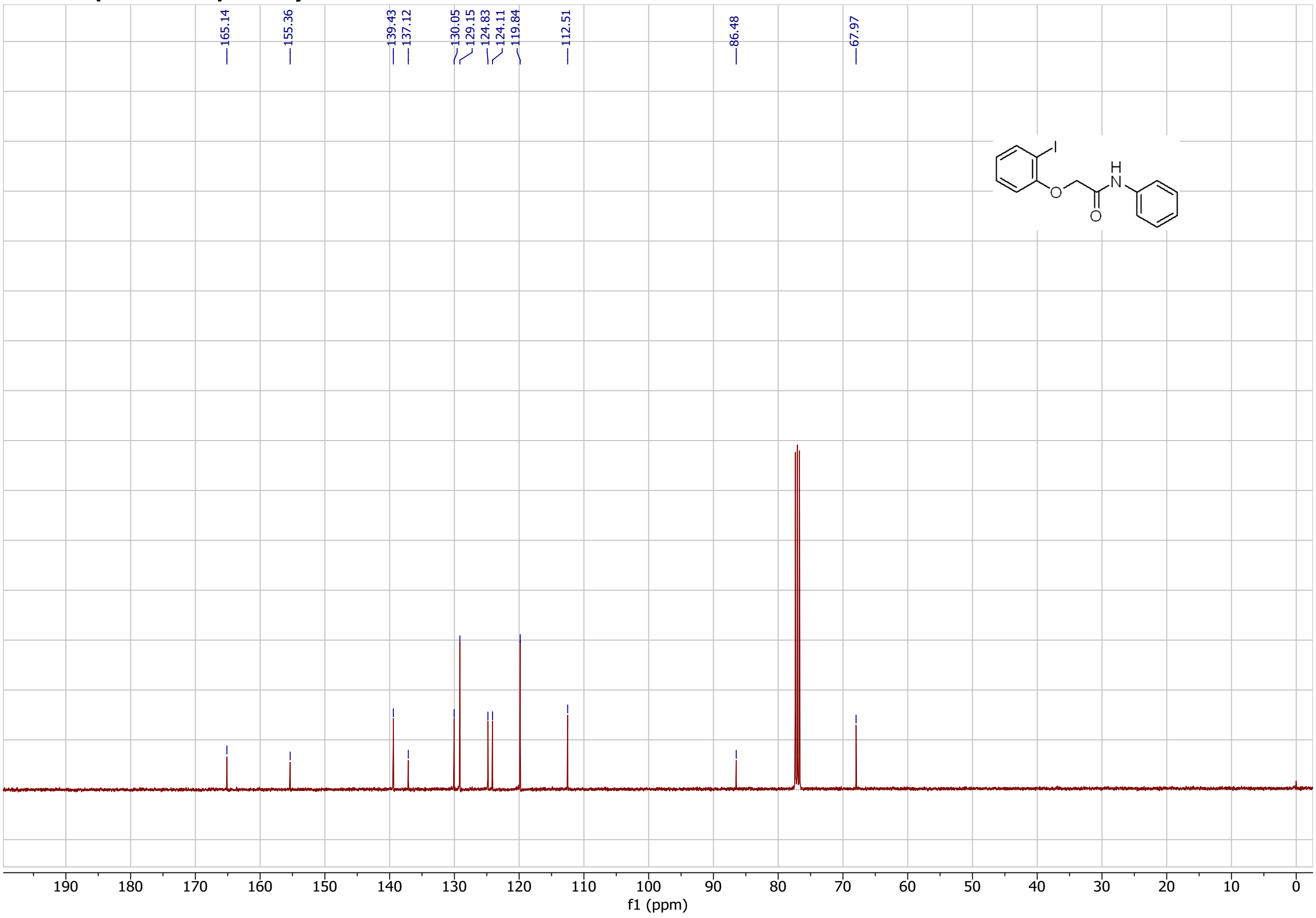

^{1}H NMR (400 MHz) Analysis of 4f.

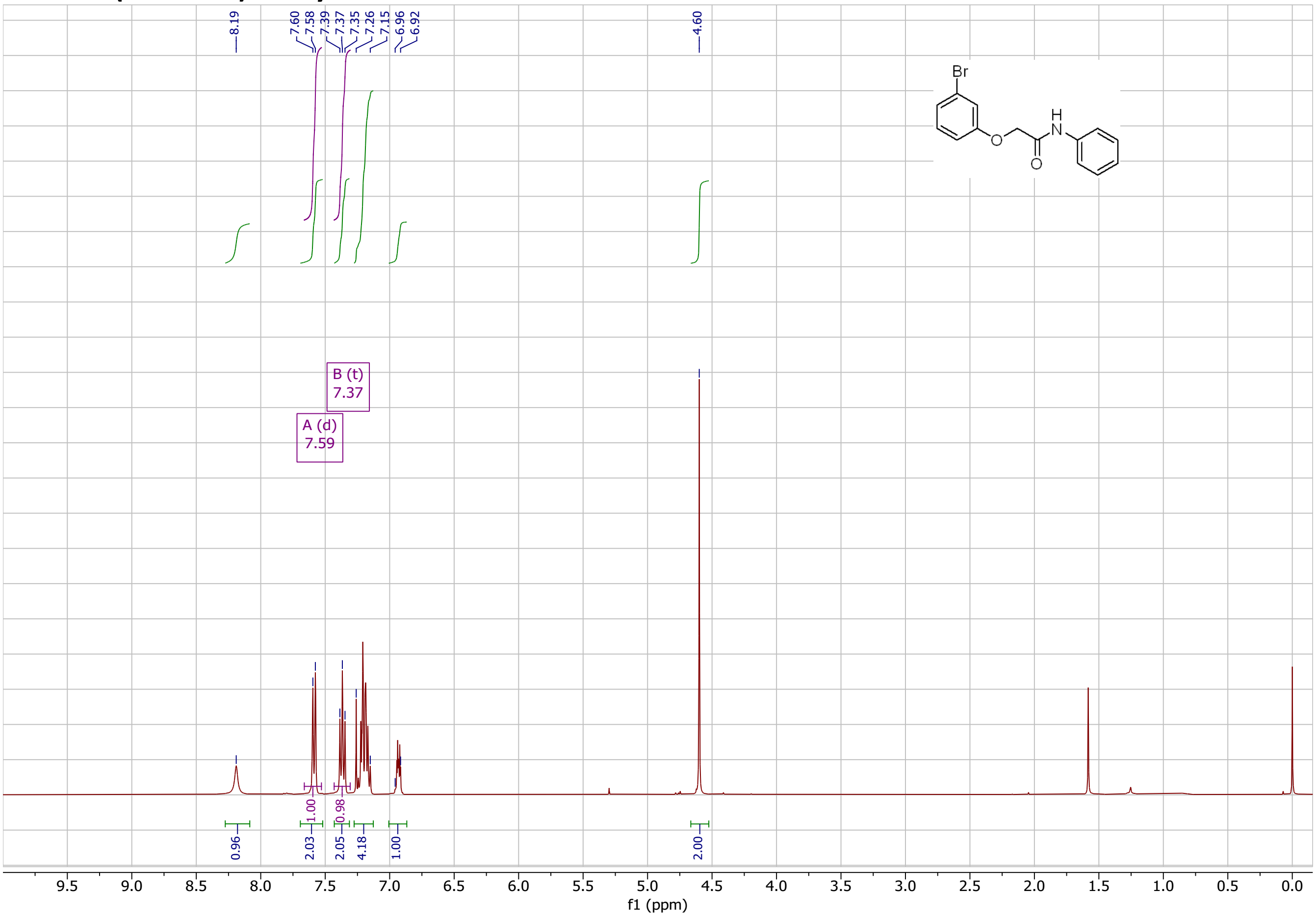

13C NMR (101 MHz) Analysis of 4f.

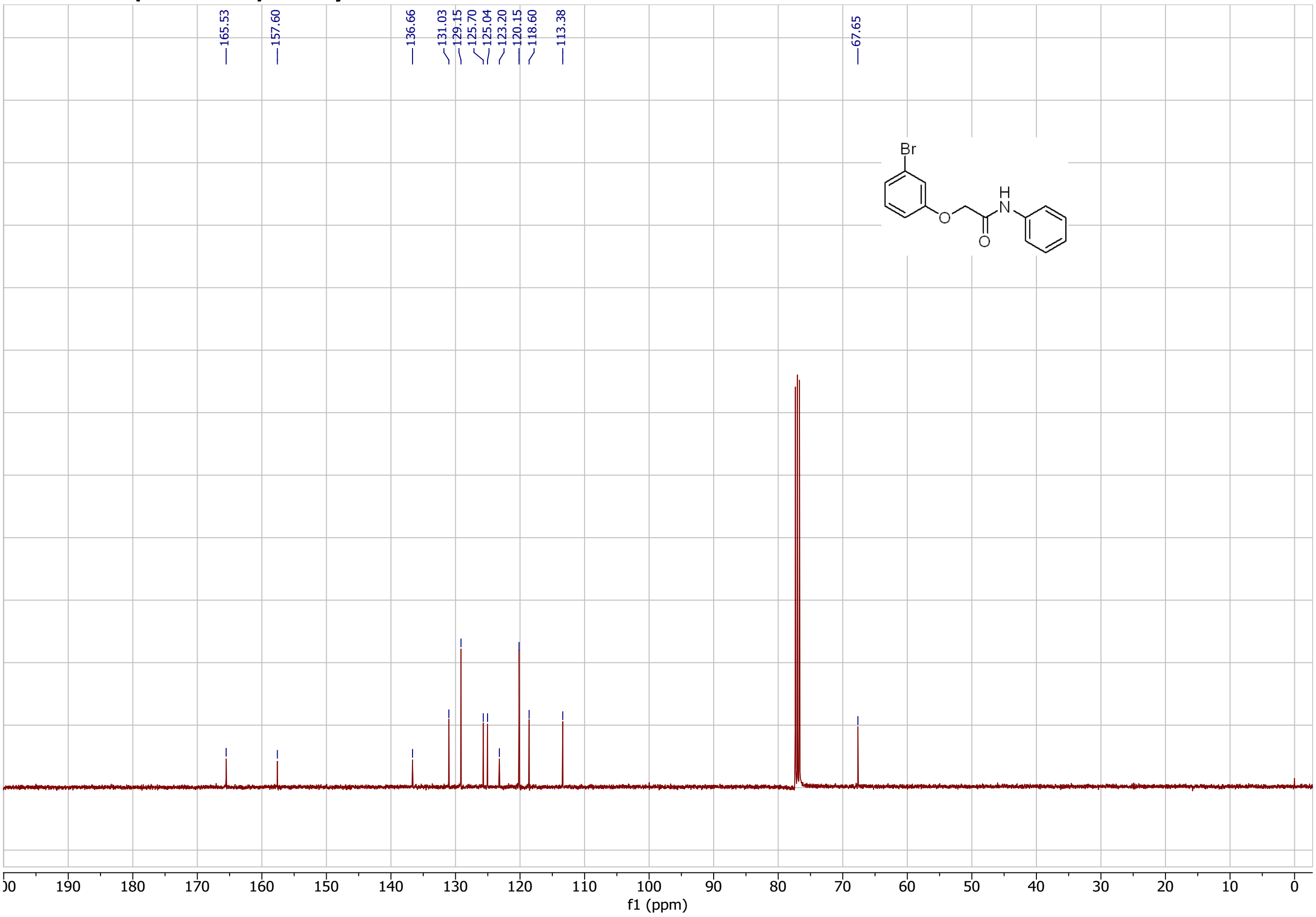

^{1}H NMR (400 MHz) Analysis of 4g

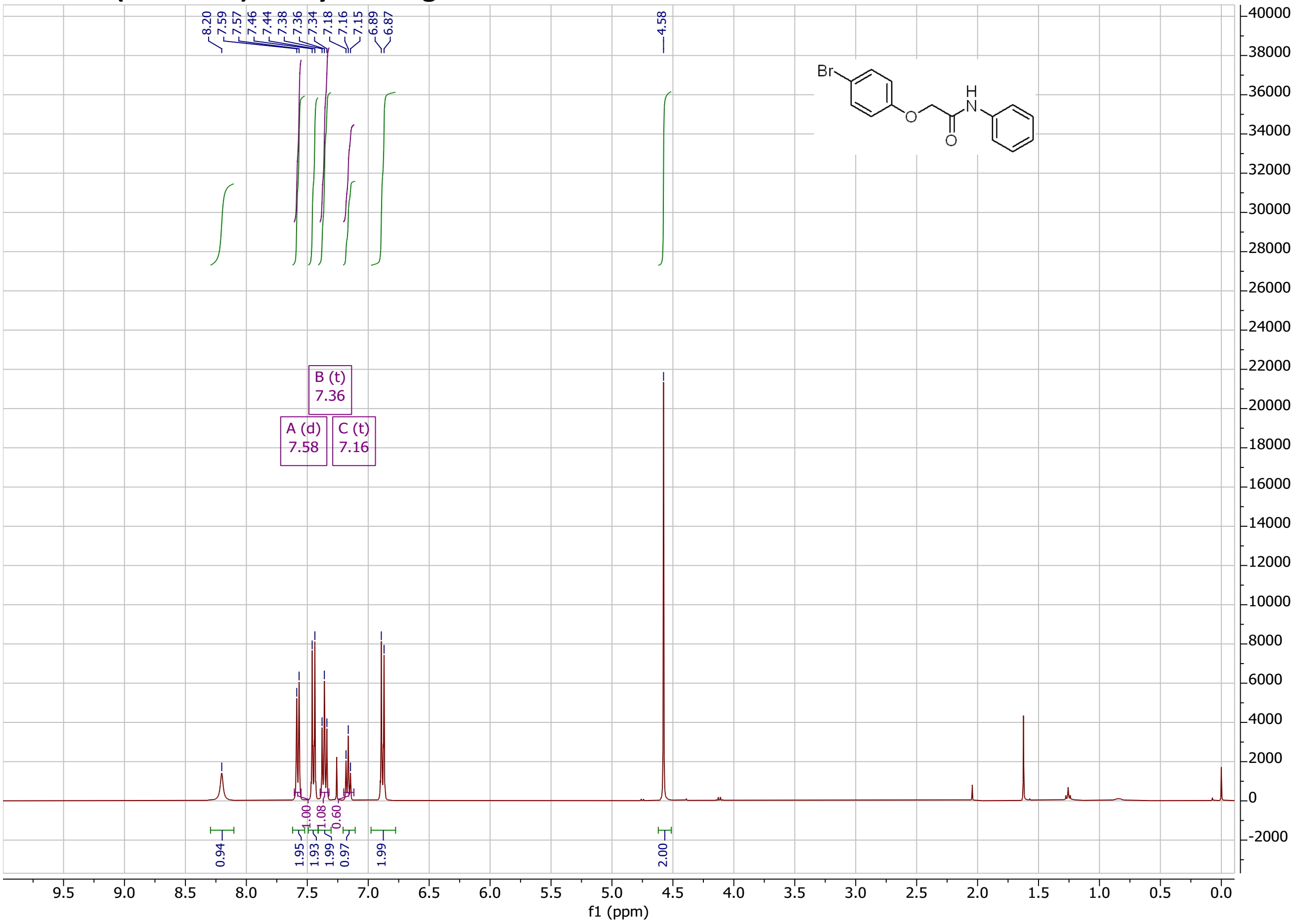

13C NMR (101 MHz) Analysis of 4g.

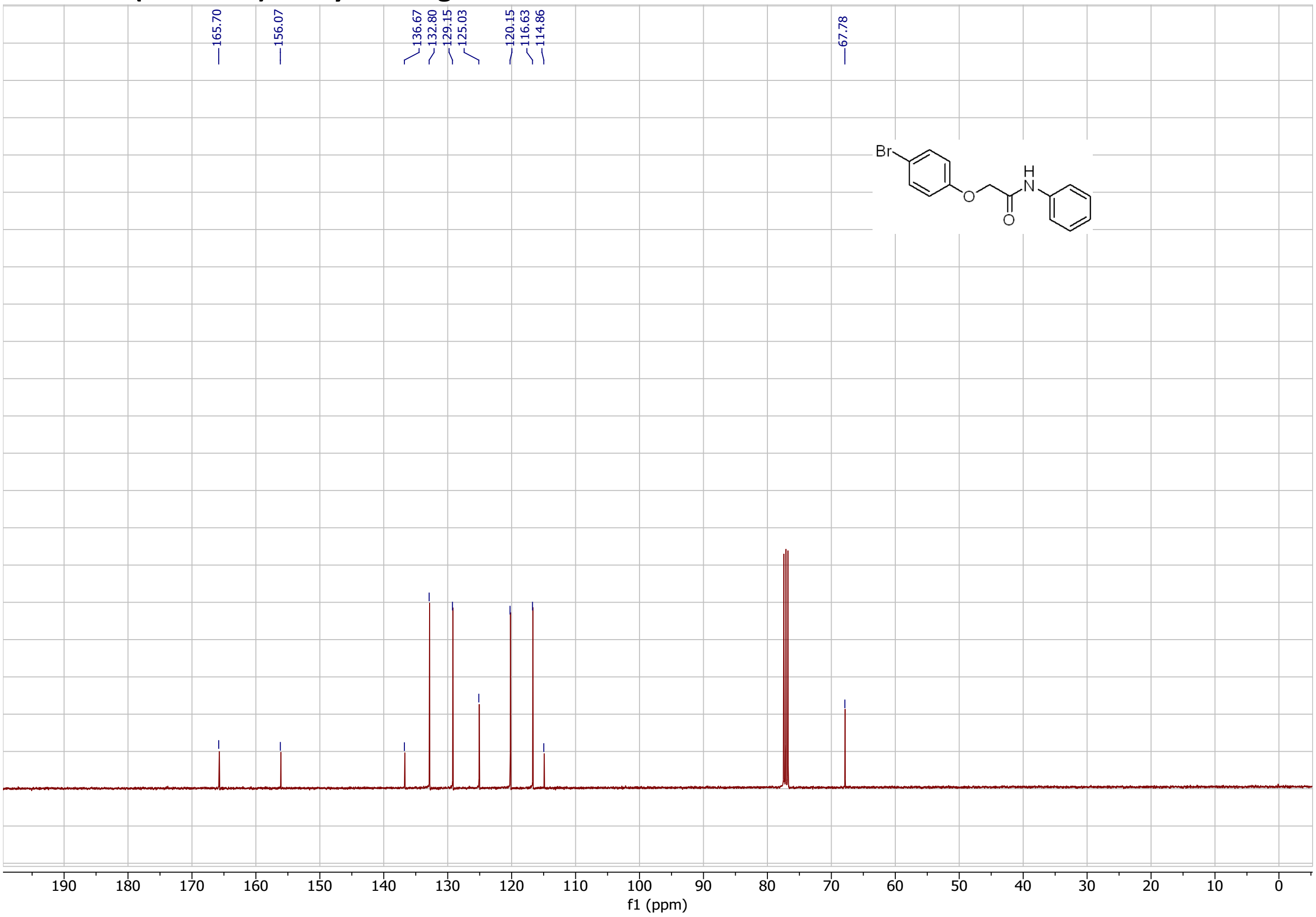

^{1}H NMR (400 MHz) Analysis of 4h.

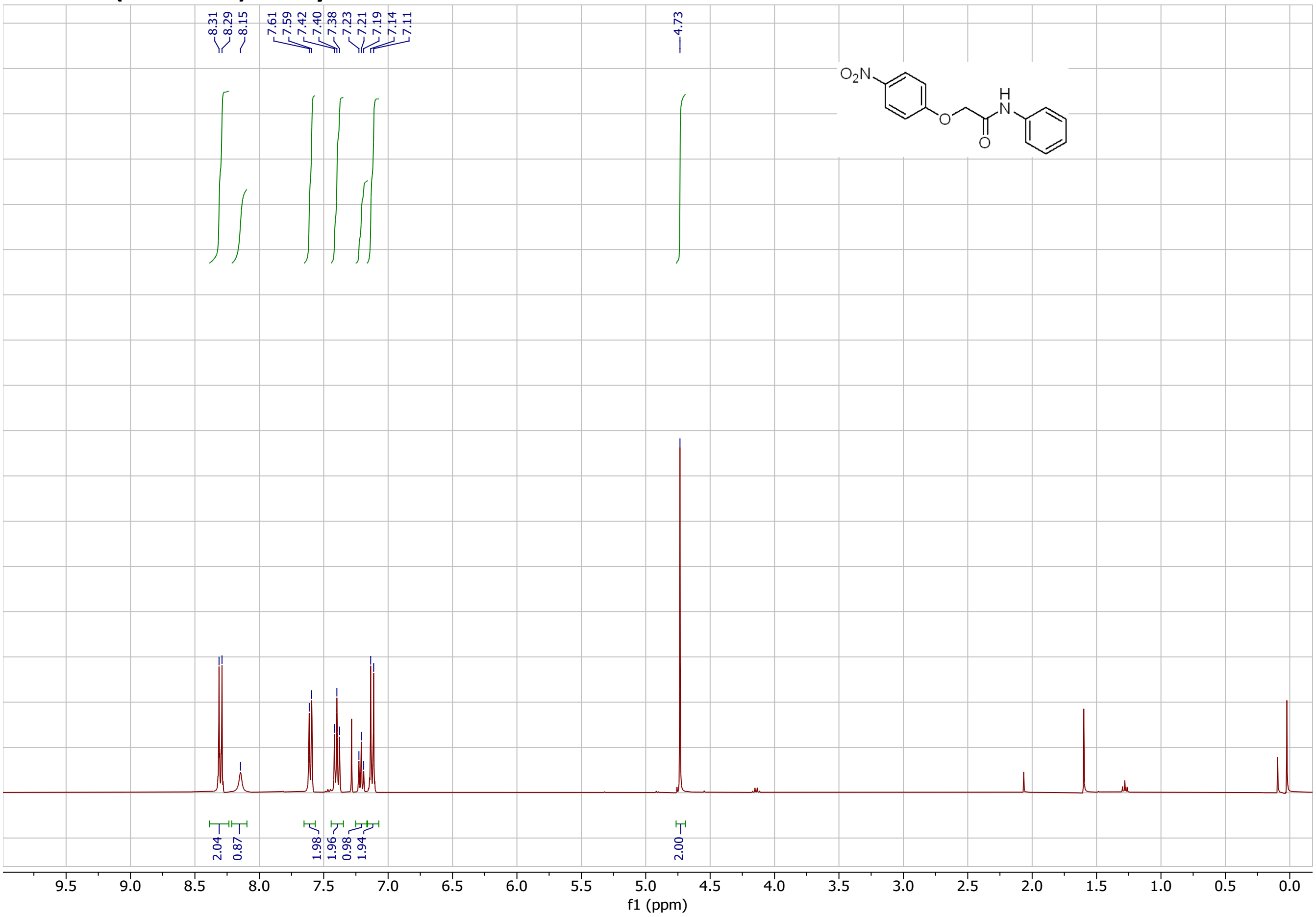

^{13}C NMR (101 MHz) Analysis of 4h.

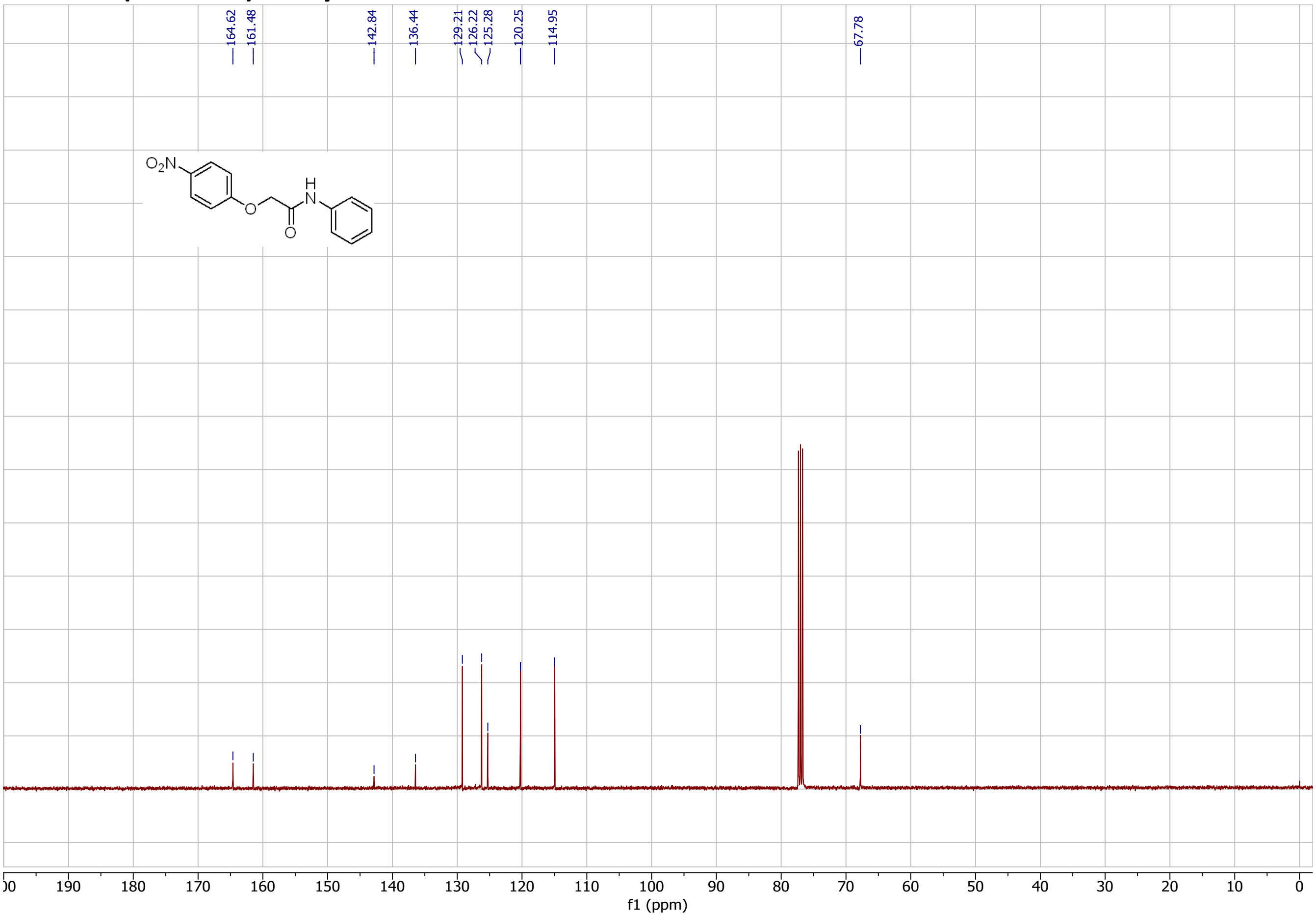

^{1}H NMR (400 MHz) Analysis of 4i.

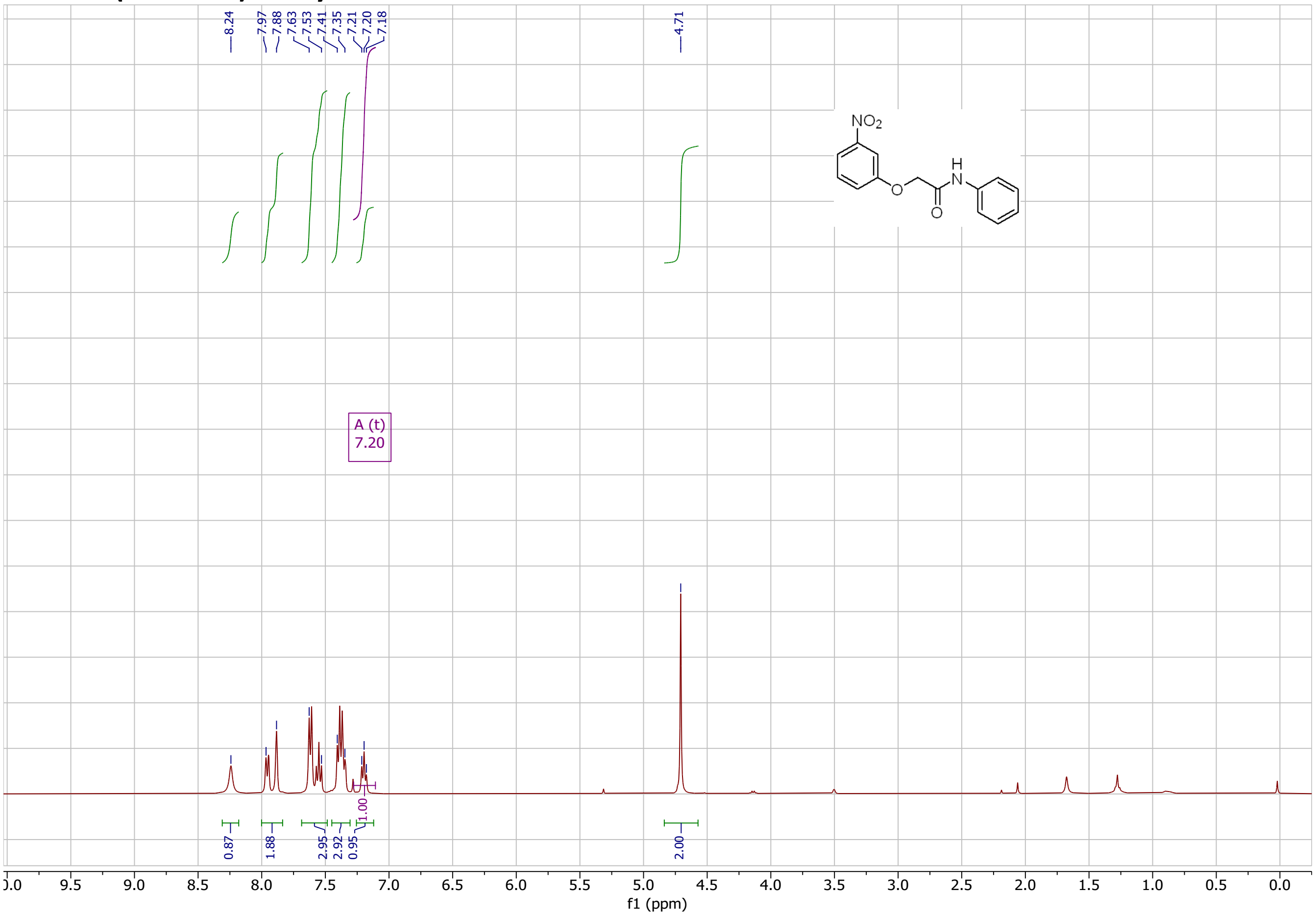

^{13}C NMR (101 MHz) Analysis of 4i.

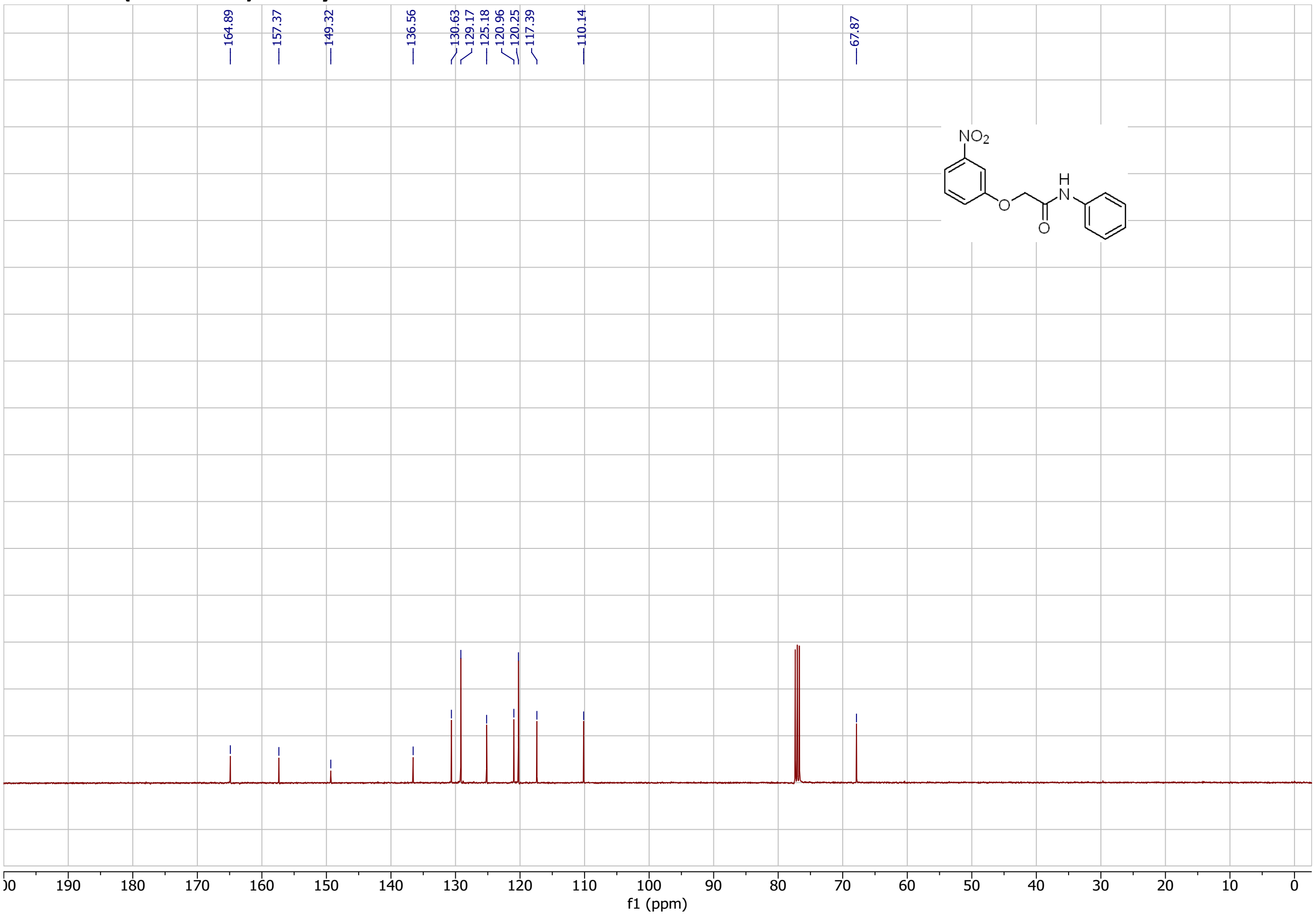

^{1}H NMR (400 MHz) Analysis of 4j.

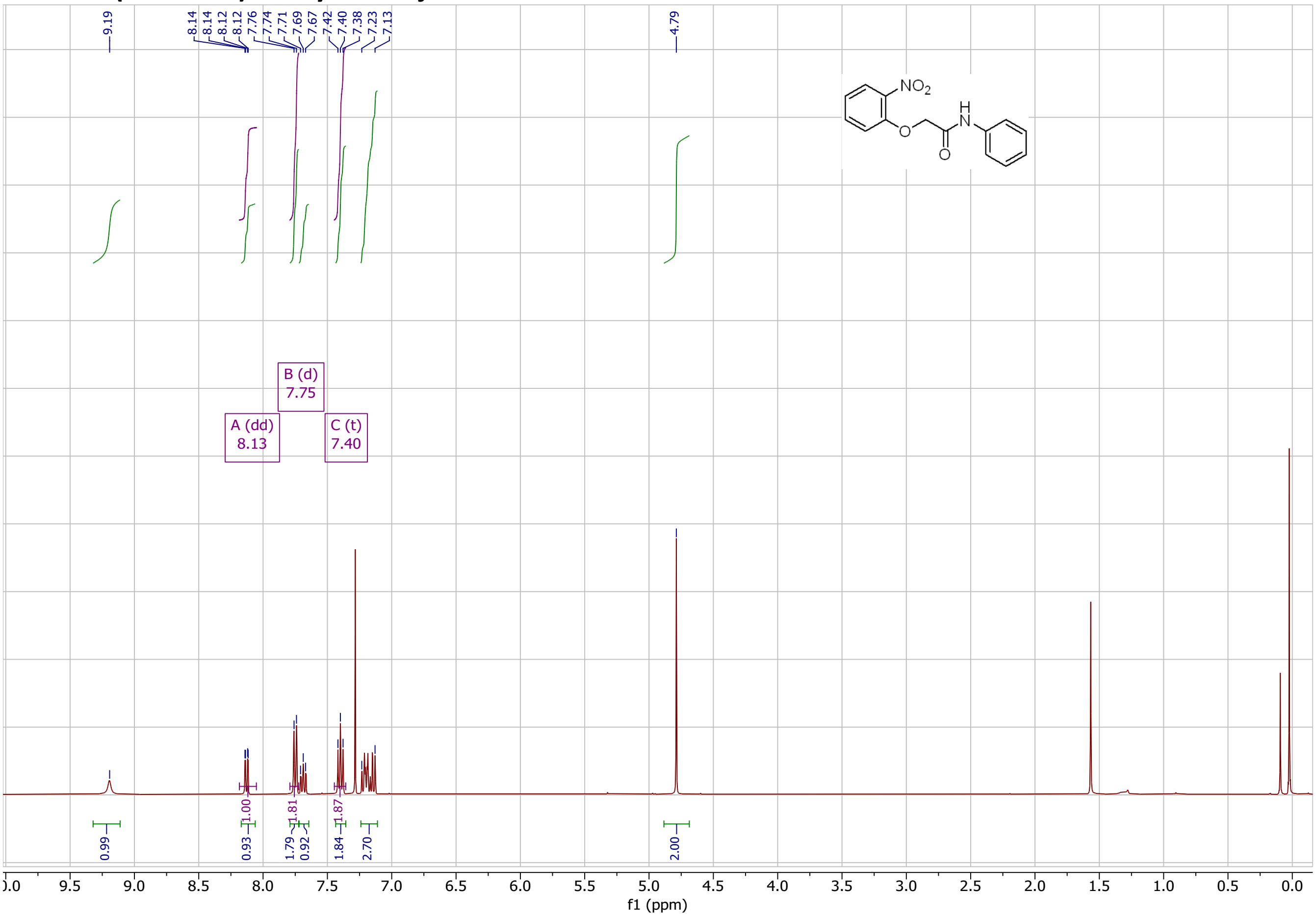

^{13}C NMR (101 MHz) Analysis of 4j.

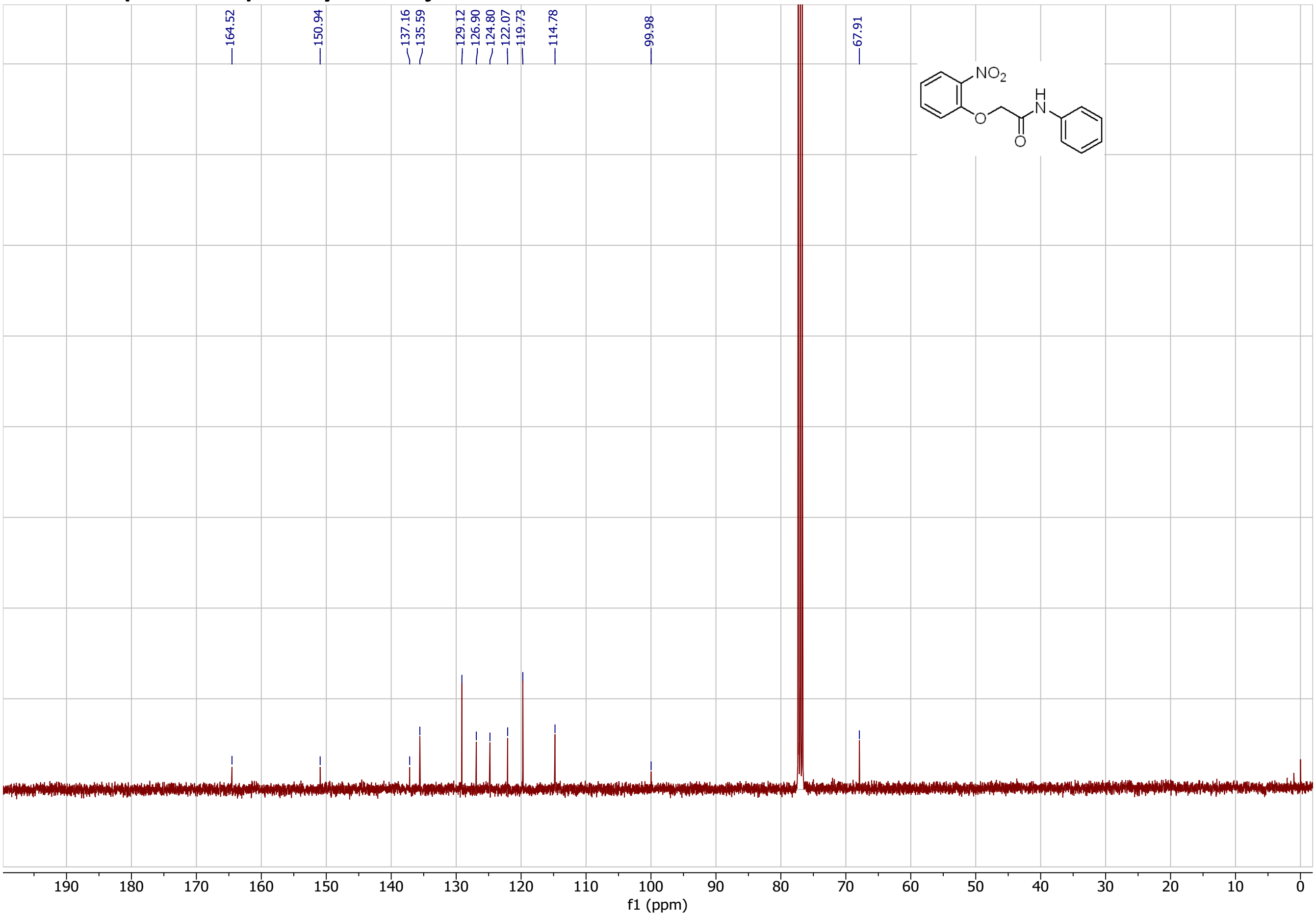

^{1}H NMR (400 MHz) Analysis of 4k.

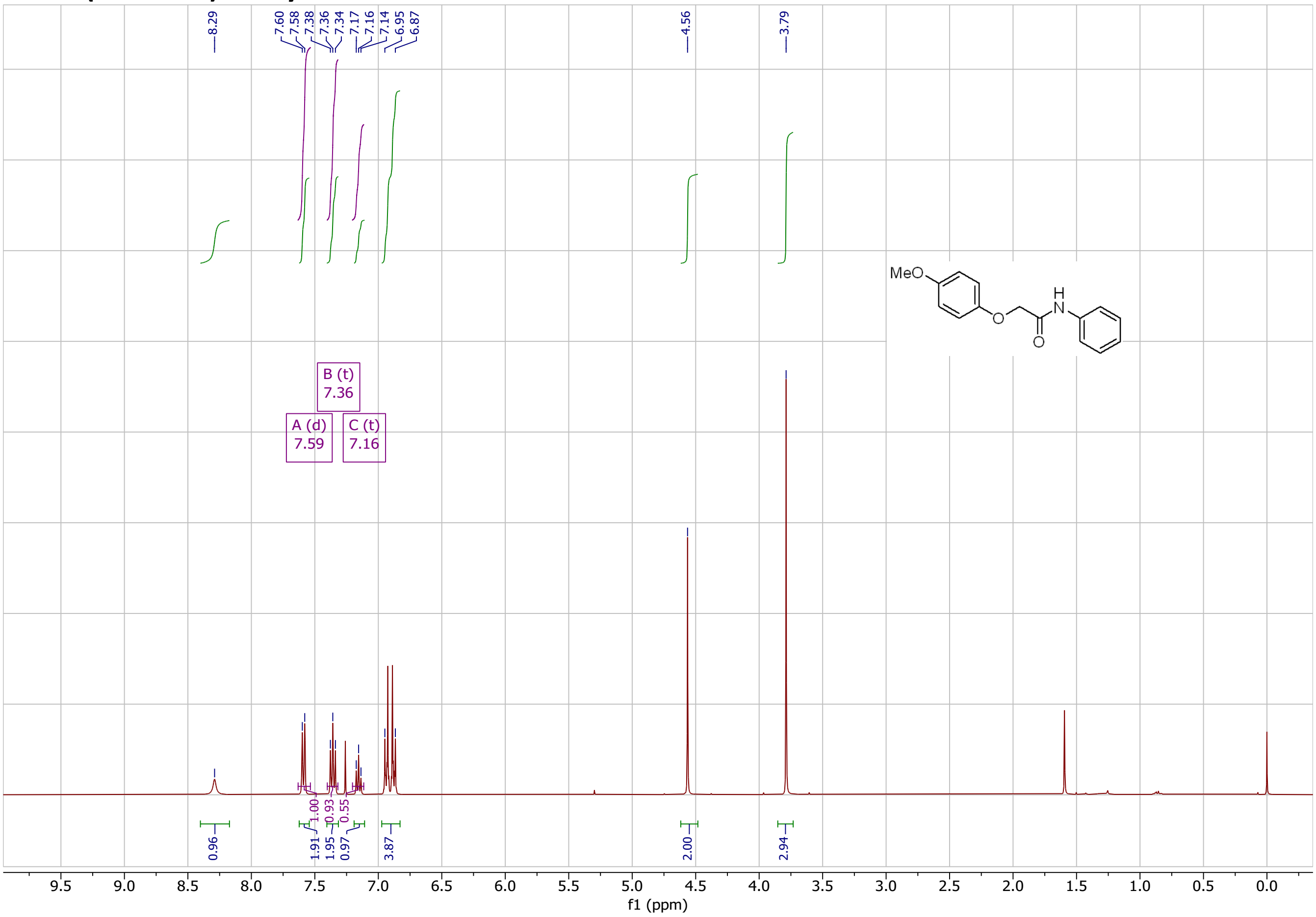

^{13}C NMR (101 MHz) Analysis of 4k.

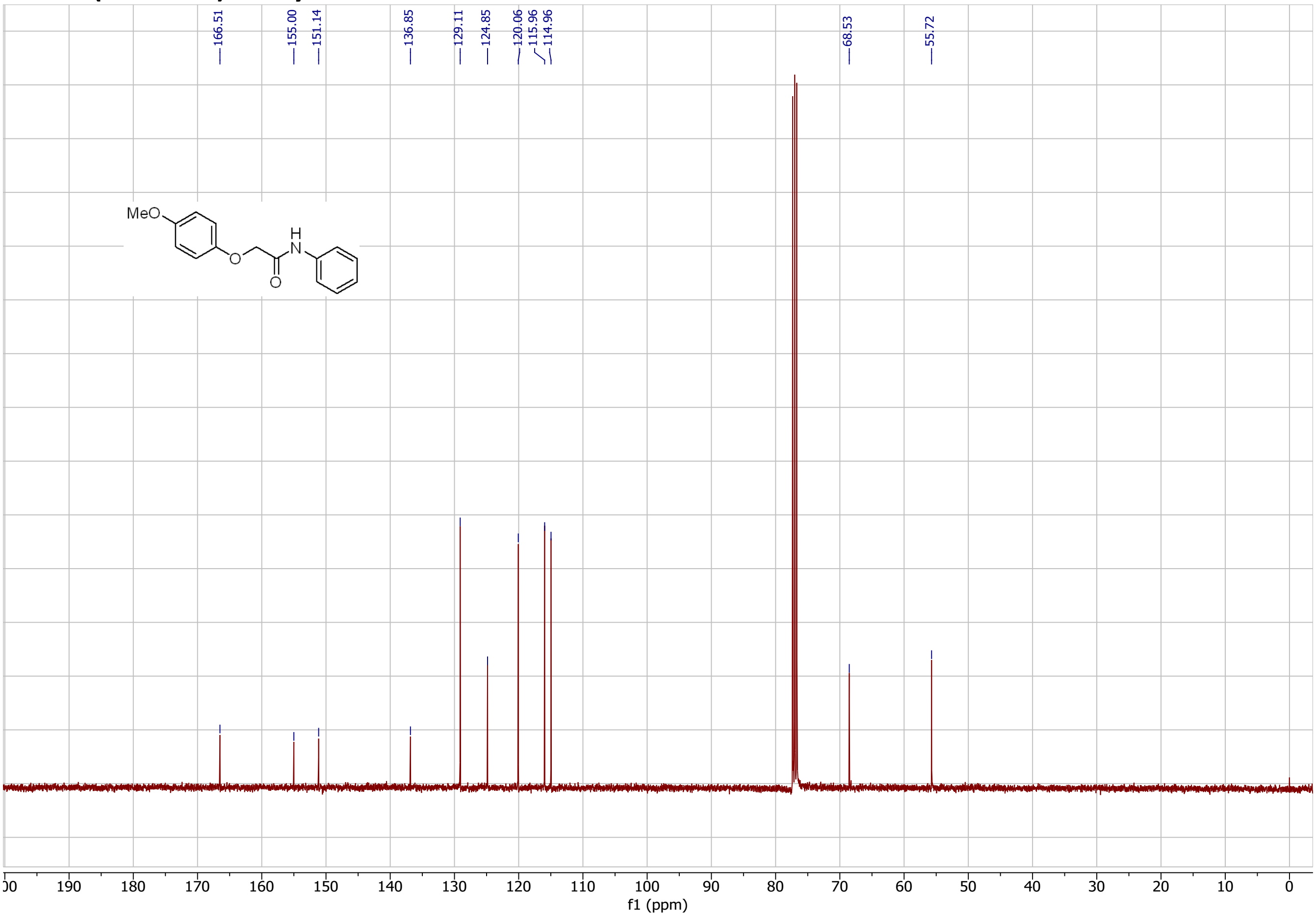

^{1}H NMR (400 MHz) Analysis of 4l.

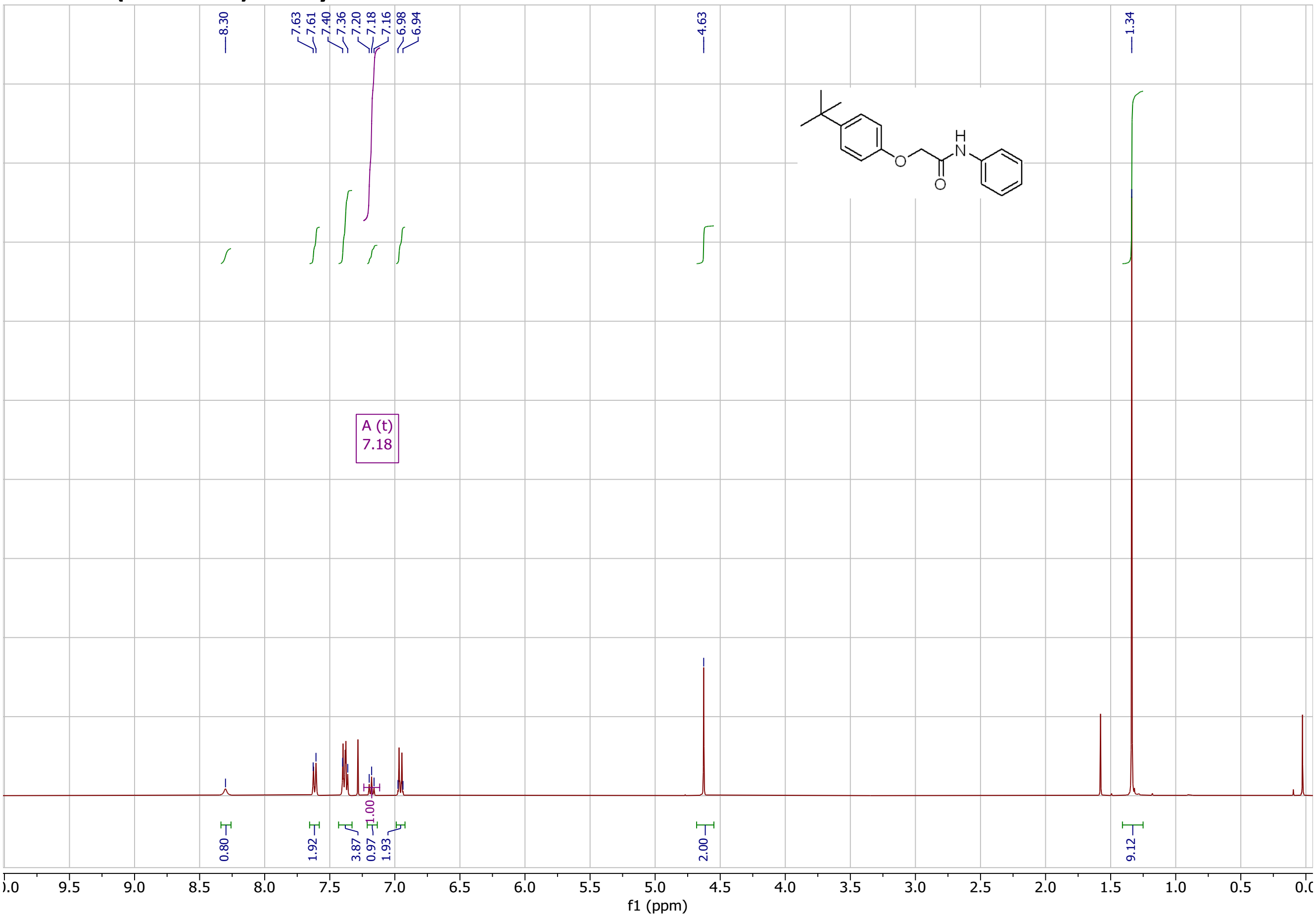

^{13}C NMR (101 MHz) Analysis of 4l.

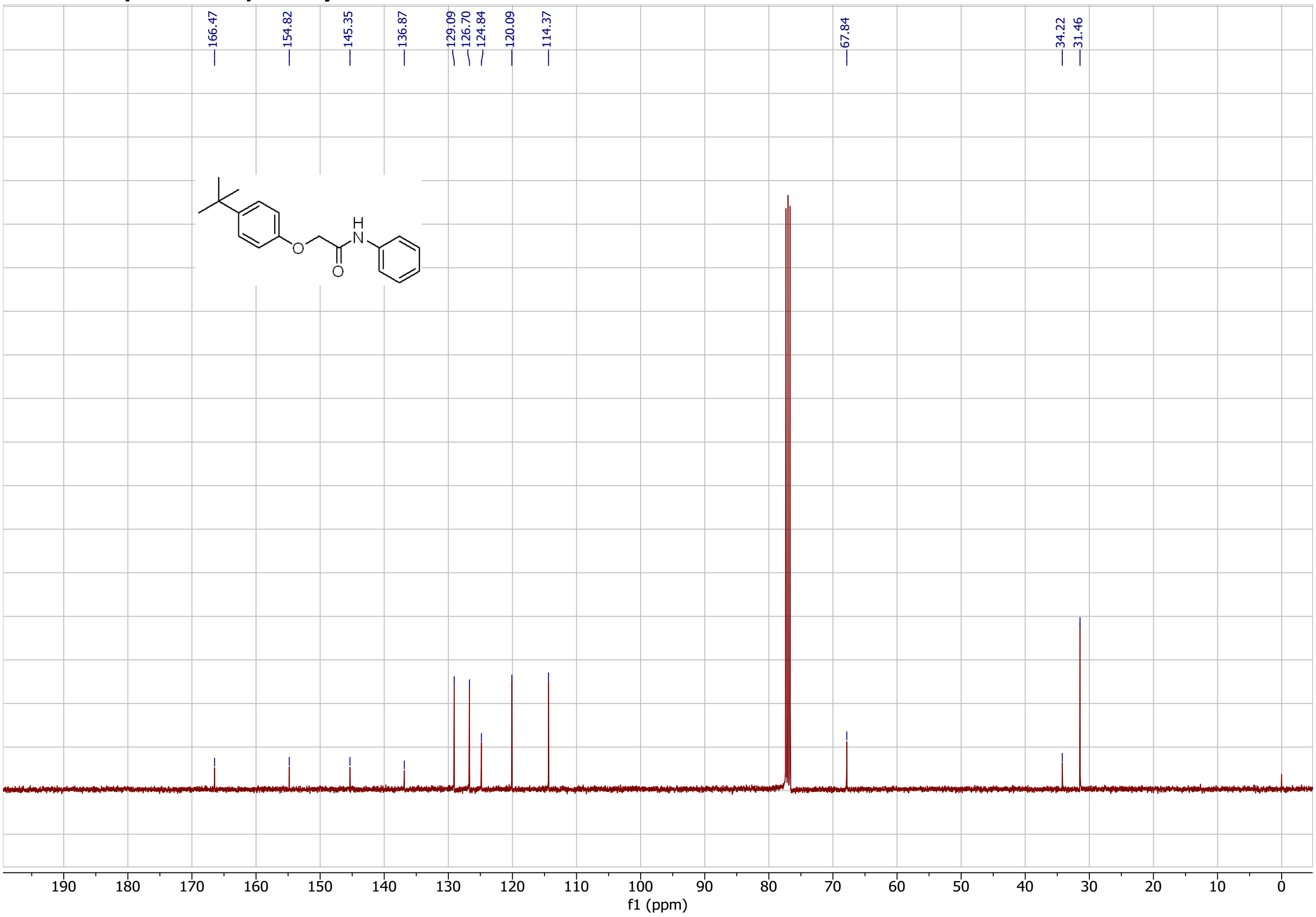

^{1}H NMR (400 MHz) Analysis of 4m.

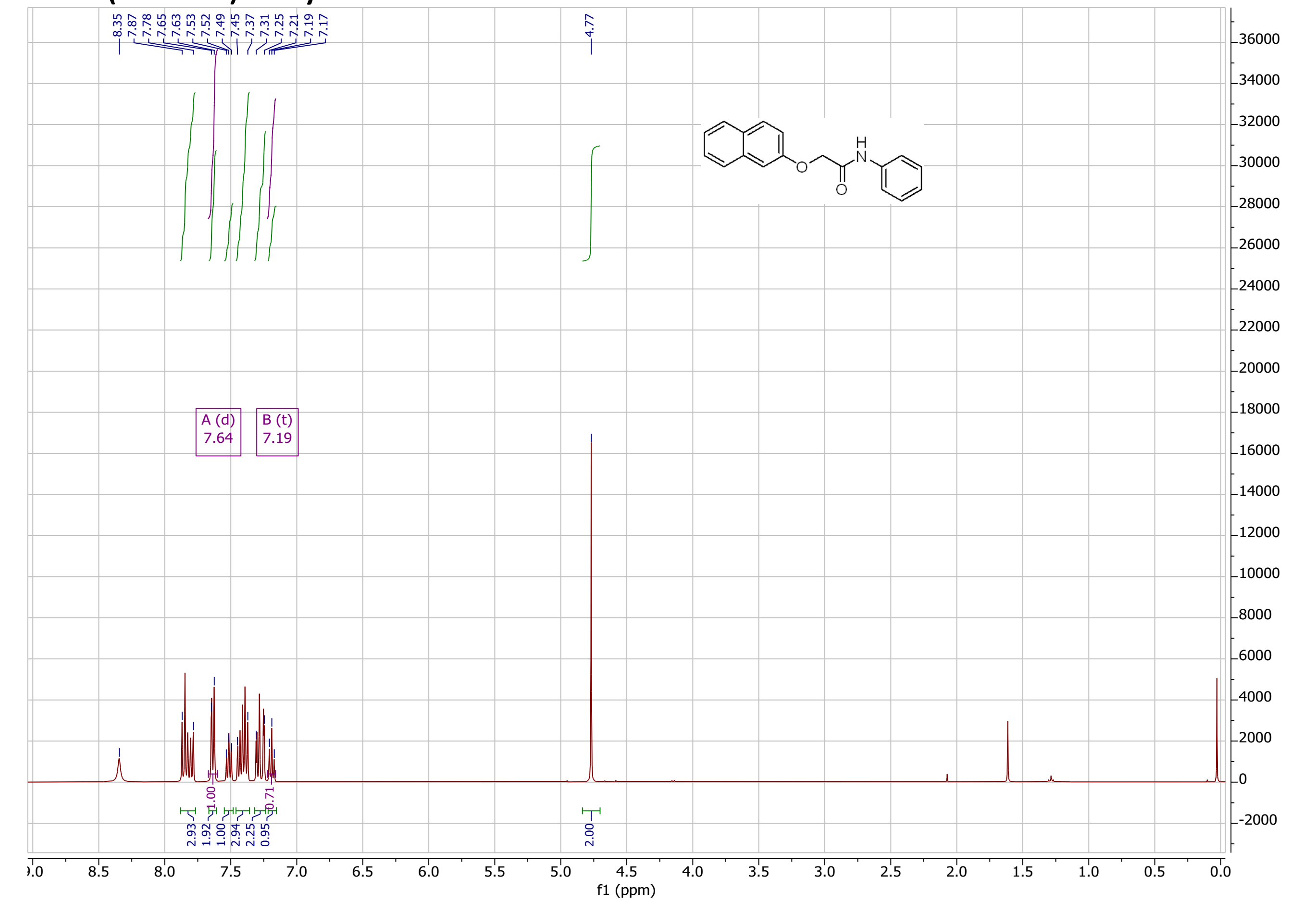

^{13}C NMR (101 MHz) Analysis of 4m.

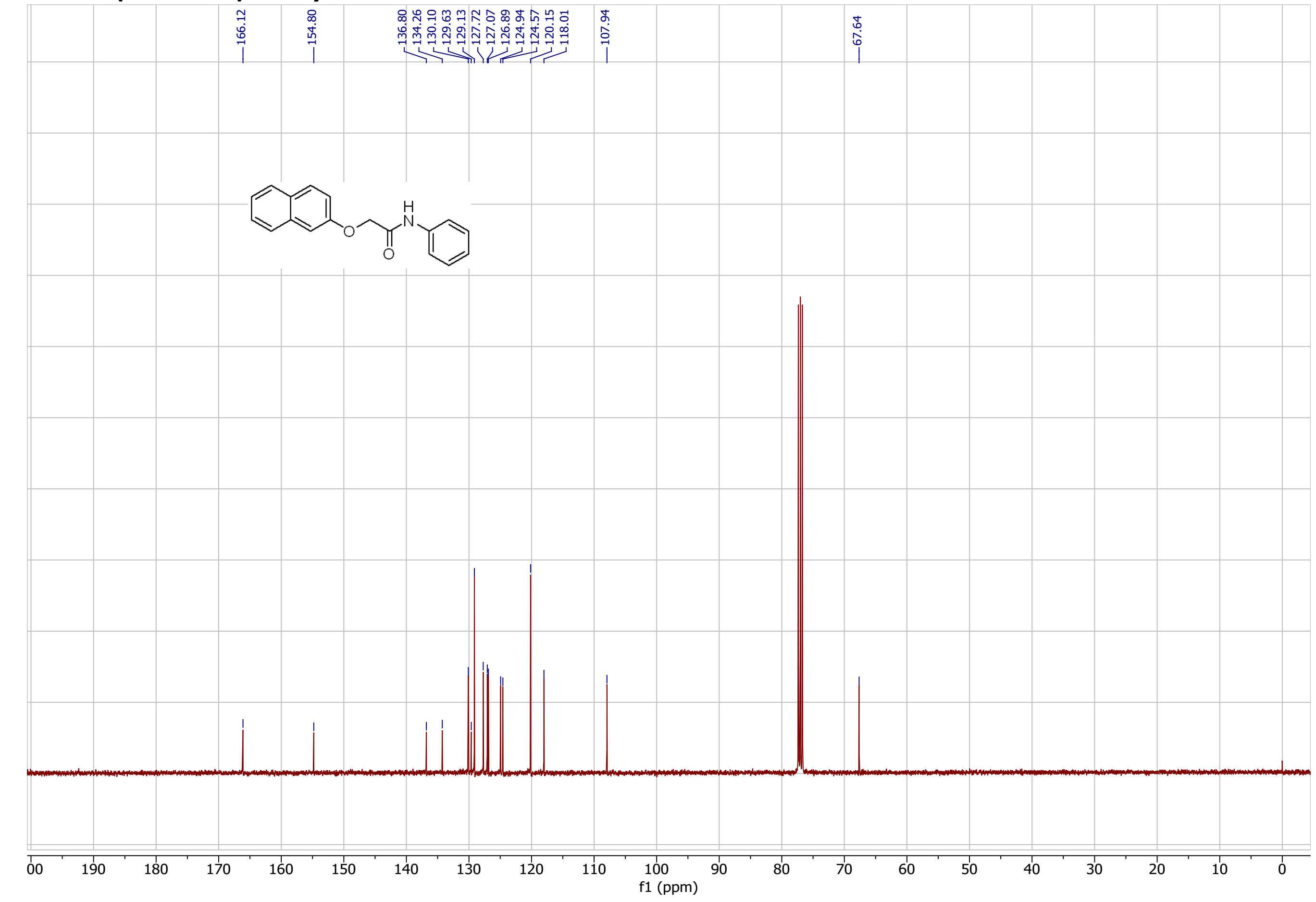

[1] H NMR (400 MHz) Analysis of 4n.

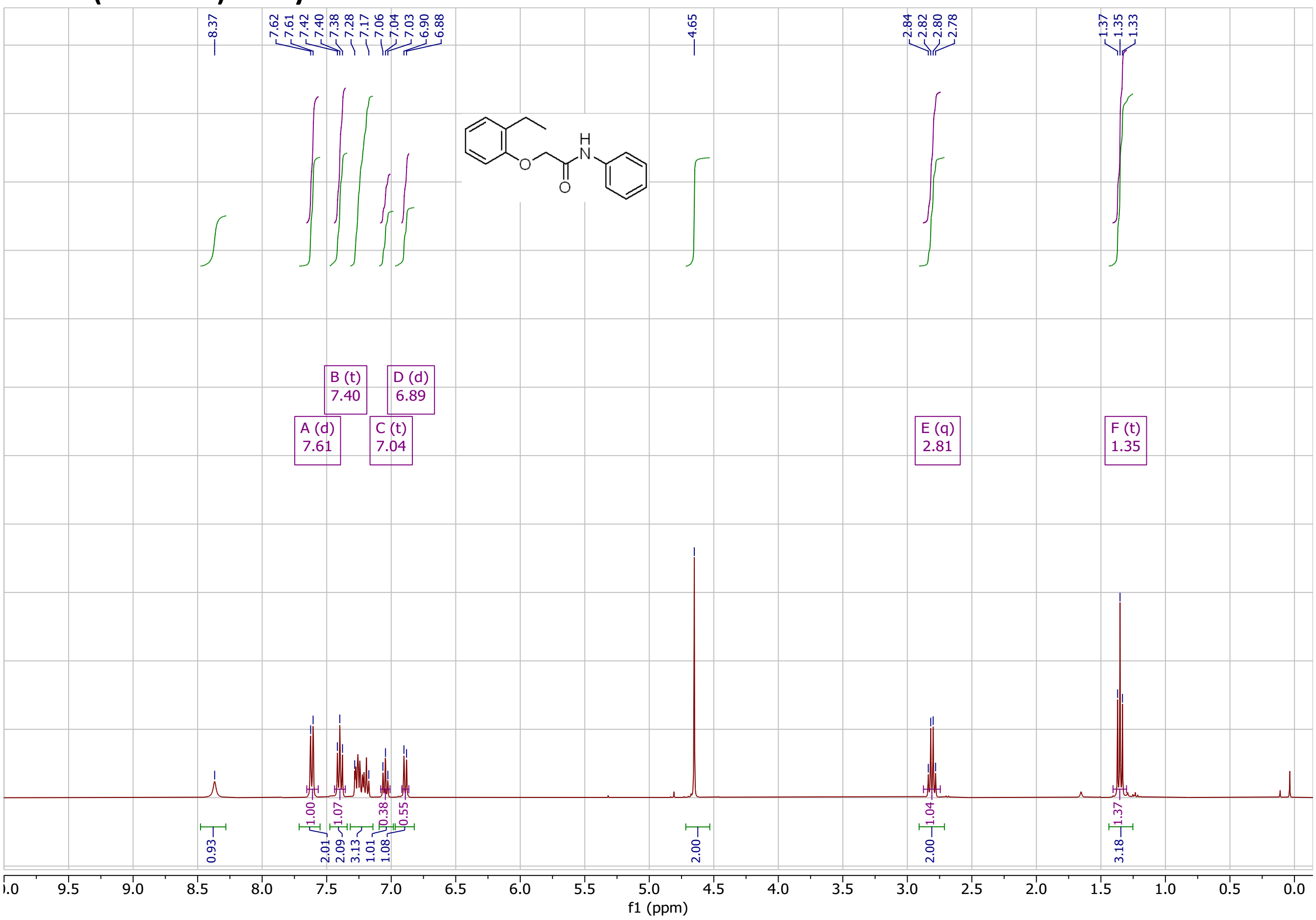

^{13}C NMR (101 MHz) Analysis of 4n.

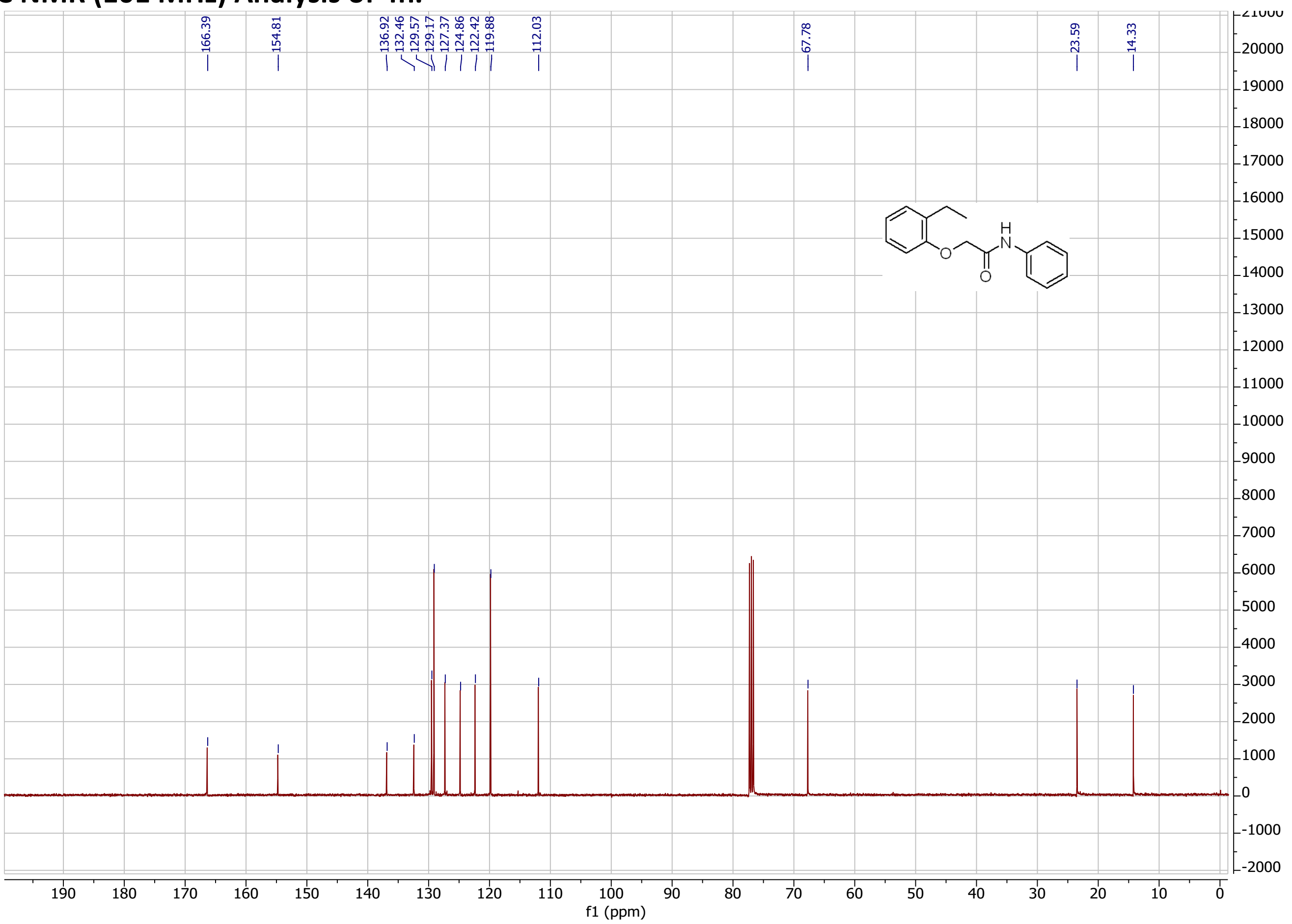

^{1}H NMR (400 MHz, $CDCl_3$) Analysis of 4o.

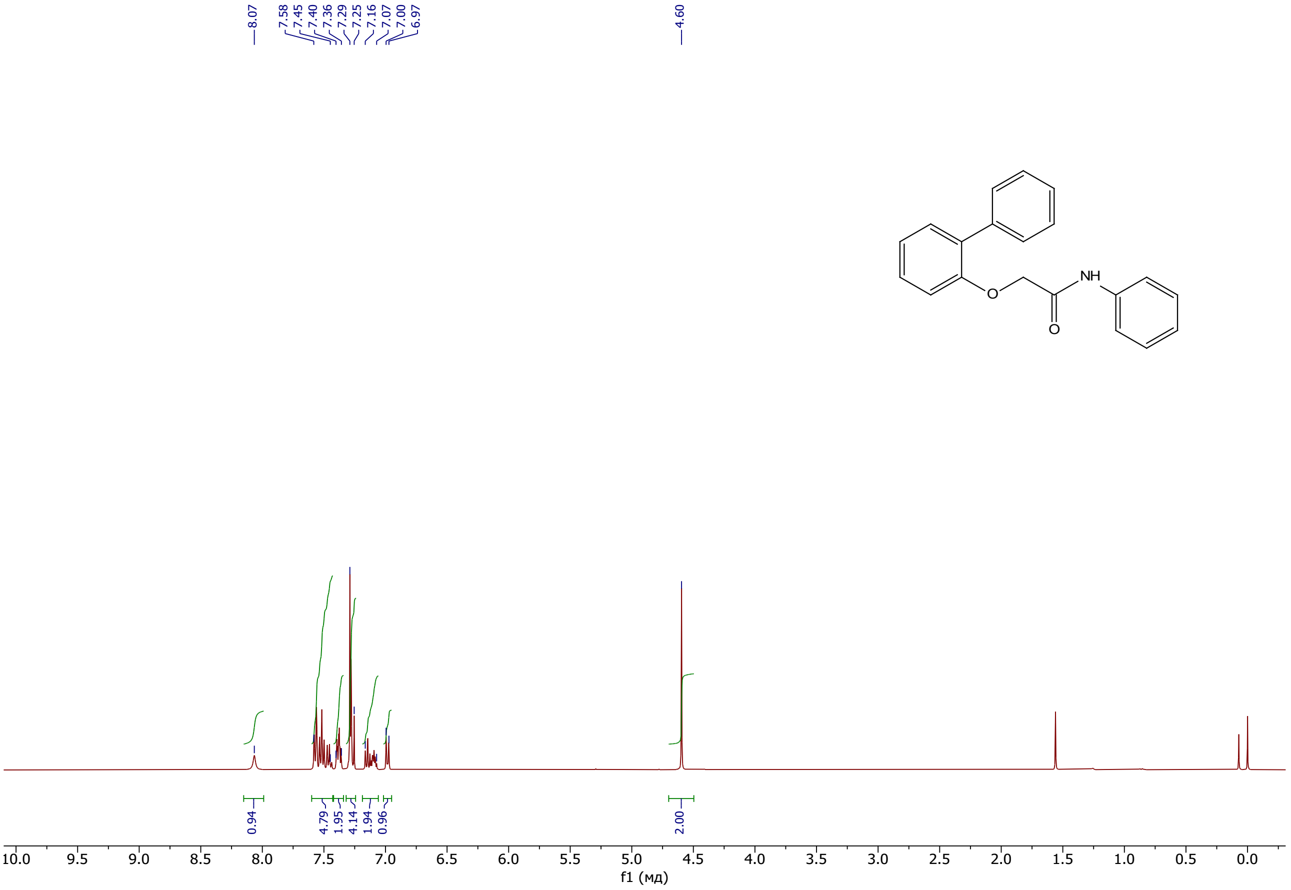

^{13}C NMR (101 MHz, $CDCl_3$) Analysis of 4o.

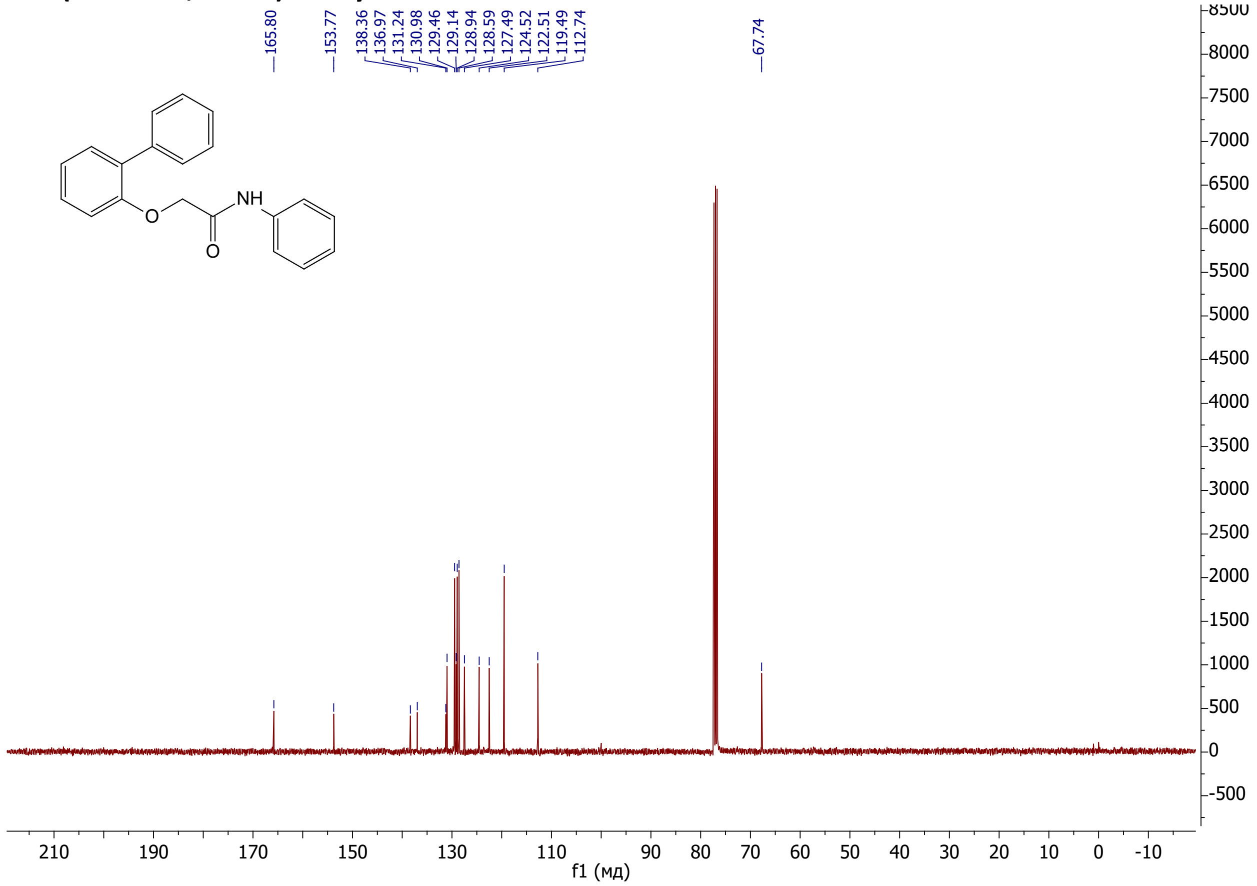

^{1}H NMR (400 MHz) Analysis of 4p.

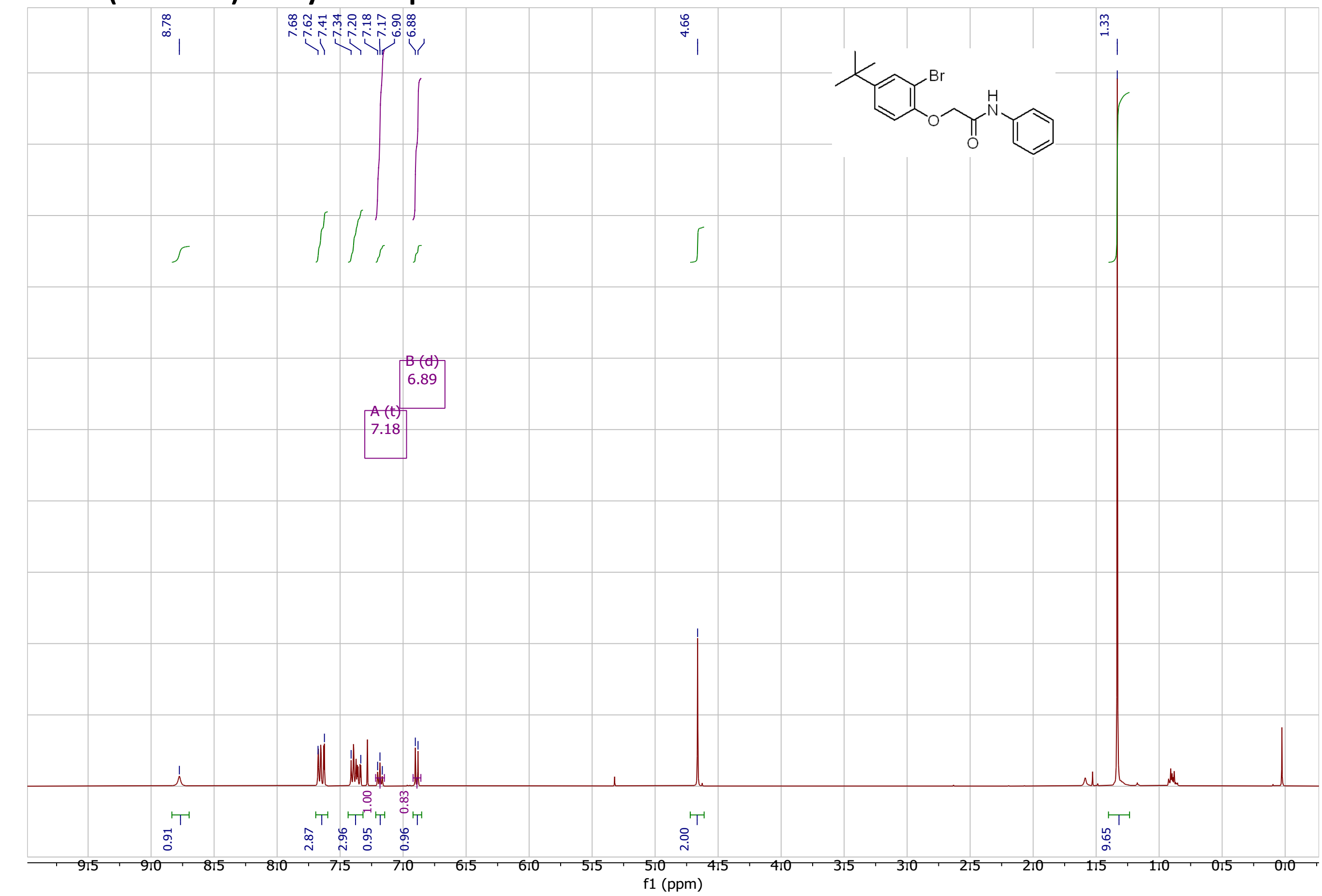

^{13}C NMR (101 MHz) Analysis of 4p.

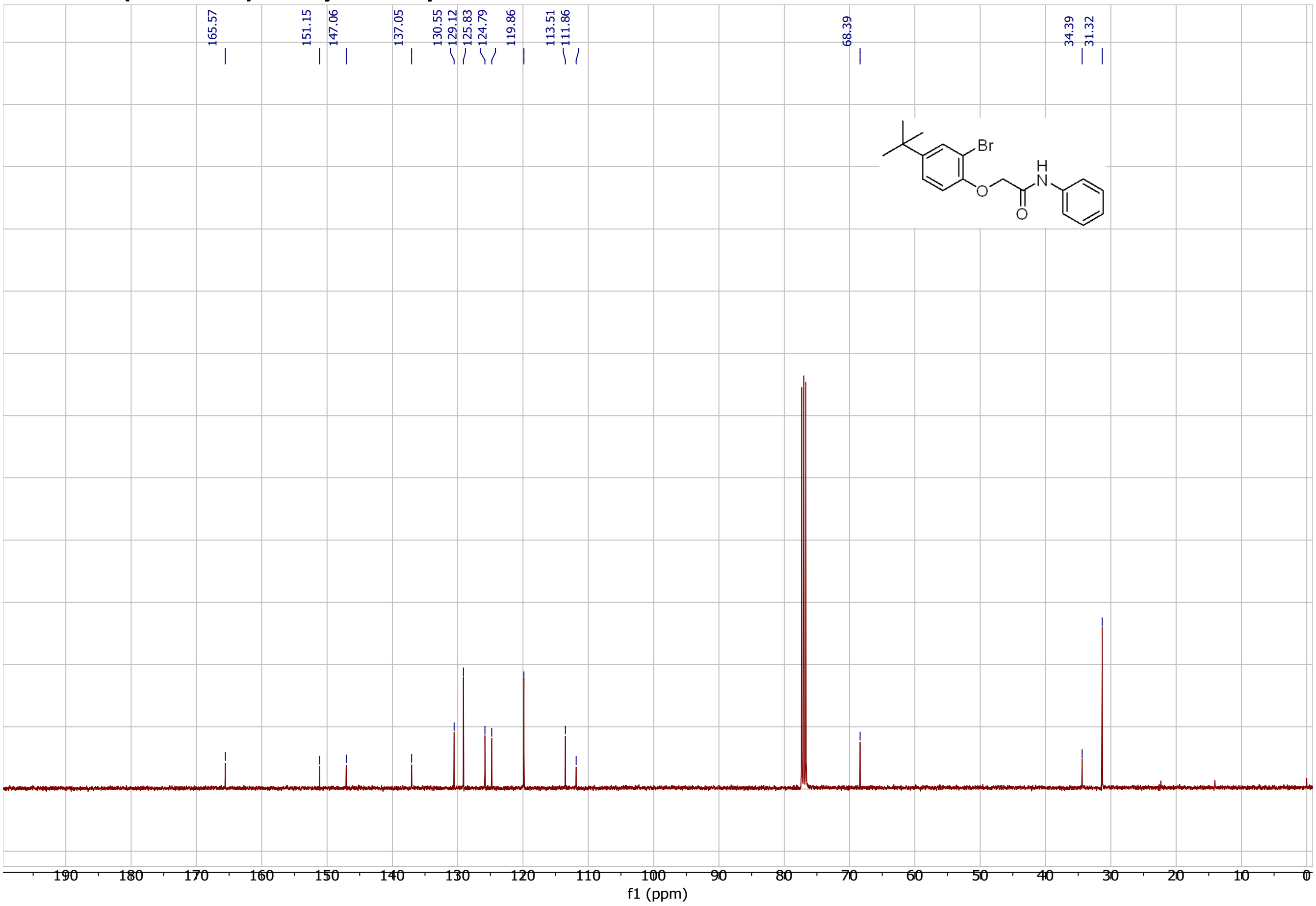

^{1}H NMR (400 MHz) Analysis of 5.

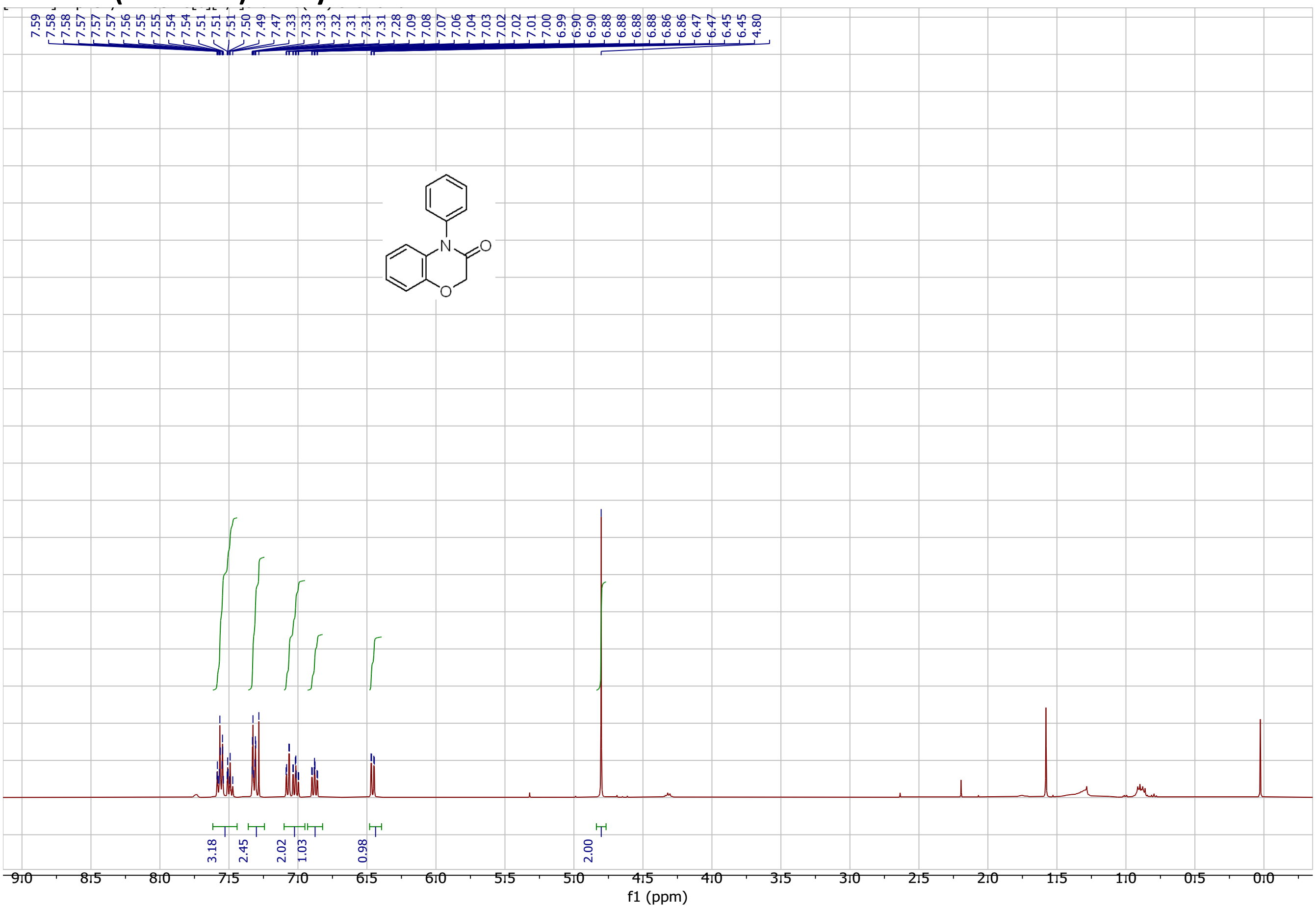

^{13}C NMR (101 MHz) Analysis of 5.

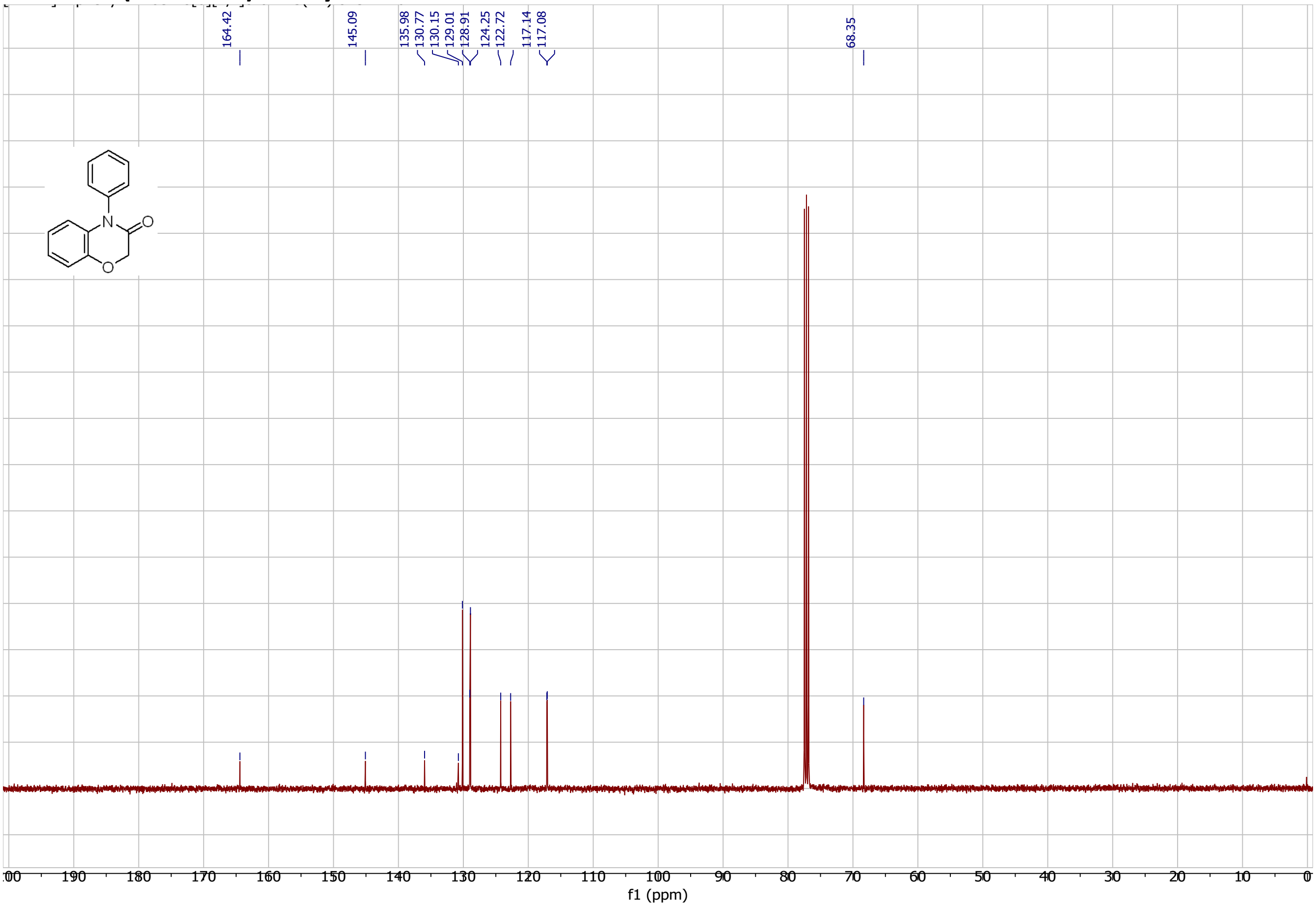

Supplement: RA-011-D1RA02305E-s001 [file RA-011-D1RA02305E-s001.pdf]
